# Coordination Chemistry of Potentially S,N,N_py_-Tridentate Thiosemicarbazones with the {Re(CO)_3_}^+^ Fragment and Formation of Hemiaminal Derivatives

**DOI:** 10.1021/acs.inorgchem.2c03259

**Published:** 2022-12-22

**Authors:** Saray Argibay-Otero, Rosa Carballo, Ezequiel M. Vázquez-López

**Affiliations:** † Departamento de Química Inorgánica, Facultade de Química, Instituto de Investigación Sanitaria Galicia Sur, Universidade de Vigo, Campus Universitario, E-36310 Vigo, Galicia, Spain; ‡ Metallosupramolecular Chemistry Group, Galicia South Health Research Institute (IIS Galicia Sur), SERGAS-UVIGO, E-36213 Vigo, Galicia, Spain

## Abstract

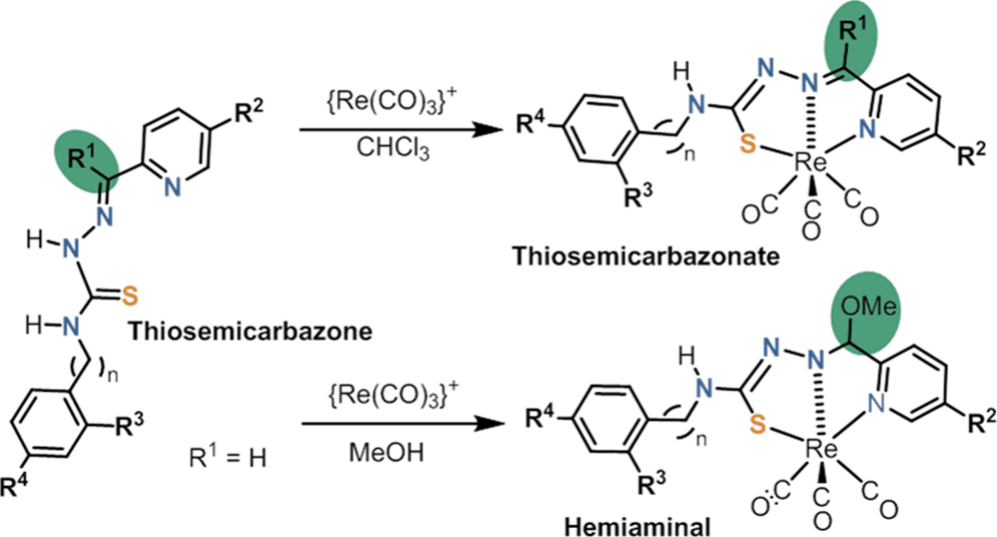

Nine potentially S,N,N_py_-tridentate thiosemicarbazones (HL) derived from pyridine-2-carbaldehyde or 1-(2-pyridyl)ethanone have been prepared and fully characterized. The X-ray crystal structures of six of them and two hydrochlorides were determined and analyzed. The reaction of the [ReX(CH_3_CN)_2_(CO)_3_]/[ReX(CO)_5_] (X = Cl and Br) precursors with these ligands yielded different kinds of compounds: the adducts [ReX(HL)(CO)_3_], in which the ligands were S,N-bidentate; the trinuclear species **[Re**_**3**_**Cl**_**2**_**(L**^**23**^**)(HL**^**23**^**)(CO)**_**9**_**];** and the thiosemicarbazonate compounds [Re(L)(CO)_3_], where the ligand is S,N,N_py_-tridentate. Besides, the reaction in methanol or ethanol of the thiosemicarbazones derived from aldehydes yielded S,N,N_py_-tridentate hemiaminal cationic [Re(HL^OR^)(CO)_3_]X and neutral [Re(L^OMe^)(CO)_3_] complexes after the coordinated ligand underwent addition of the alcohol group to the imine bond. The reactivity of the complex [ReX(HL)(CO)_3_] in MeOH and NEt_3_ led to the formation of dinuclear [Re_2_(L)_2_(CO)_6_], where the thiosemicarbazonate is again S,N-bidentate. The influence that the substituents on the thiosemicarbazone ligands have on the stability of the complexes and the effect of the reaction medium on the resulting compounds have been analyzed.

## Abstract 2

The reaction of the Re(I) precursors with TSC derived from 2-pyridine yielded different kinds of compounds depending on the nature of the substituents (mainly R^1^) and the solvent. In methanol or ethanol, the TSC derived from aldehydes (R^1^ = H) yielded tridentate hemiaminal cationic [Re(HL^OEt^) (CO)_3_]X and neutral [Re(L^OMe^)(CO)_3_] complexes.

## Introduction

**Scheme 1:**
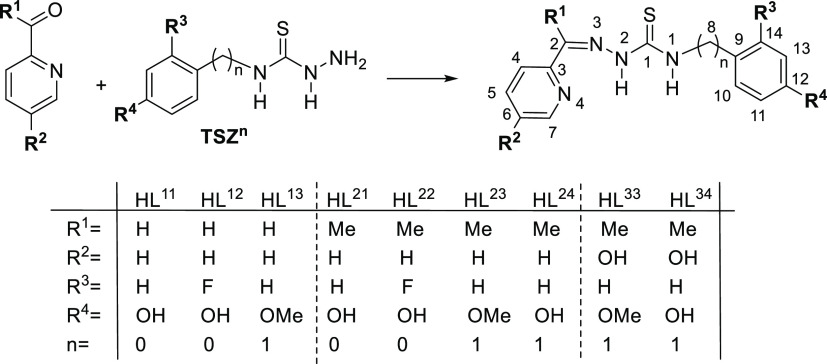
Synthesis and Numbering Scheme of the TSCs Studied in This Work

Thiosemicarbazones (TSCs) have attracted a great deal of interest from the point of view of their coordination capacity since they simultaneously allow the inclusion of a variety of different substituents and also show a wide range of possible coordination modes.^1−3^ In most complexes TSCs are coordinated by the azomethine nitrogen (N3) and sulfur atoms (Scheme 1) to form a five-membered chelate ring. In addition, deprotonation of the ligand and the formation of thiosemicarbazonate complexes are also commonly observed, and this allows the possibility of designing complexes with different charges. The introduction of other coordinatively active groups on the iminic C2 increases the denticity of the ligand. This possibility makes them especially interesting for the synthesis of radiopharmaceuticals based on the fragment {^99m^Tc(CO)_3_}^+^ and the more commonly synthesized and characterized surrogate {Re(CO)_3_}^+^, since the tridentate ligand may satisfy all of the coordinative demands of the metal center.

This tricarbonyl center has attractive advantages for the design of radiopharmaceuticals. The coordination number is always six, and the system has high geometric (with the three carbonyl ligands in facial positions) and chemical stabilities since replacement of one of the carbonyl ligands will rarely occur. However, in order to avoid the labilization of non-carbonyl ligands observed in biological media,^4^ which prevents suitable biodistribution, the use of tridentate ligands is considered necessary. In an ideal design, the ligand should also be monoanionic or neutral in order to, in turn, form neutral or cationic complexes, [M(L)(CO)_3_]^0/+^, to achieve adequate uptake by tissue.^5^

From this point of view, and taking into account the reported affinity of these acceptors for imines,^6^ TSCs with a 2-pyridine fragment on the C2 position (as shown in Scheme 1) should allow the desired S,N3,N_py_ coordination. This design was previously tested with Re^III/V^ cores and ligand derivatives of 2-pyridineformaldehyde (R^1^ = H)^7^ or pyridineformamide (R^1^ = NH_2_).^8^ However, under certain conditions, these compounds undergo reductive cleavage of the hydrazinic N–N bond, which results in the formation of the methyl(2-pyridyl)methyleneimine rhenium complex. The formation of complexes with the Re^I^/Tc^I^ fragment, despite being practically devoid of redox activity, has met with limited success. In fact, only complexes derived from ketones (R^1^ = CH_3_) or pyridineformamide (R^1^ = NH_2_) have been isolated^9,10^ and in these cases the tridentate ligand complex is sometimes not formed in biocompatible media.^10^ Despite the above limitations, when the rhenium complex is obtained, it has been shown that the corresponding ^99m^Tc complex can also be produced. This is the case for some TSC ligands with substituents that have affinity toward amyloid-β fibers, for which we were able to study their affinity toward the fibers in vitro and their biodistribution in mice.^10^

**Scheme 2:**
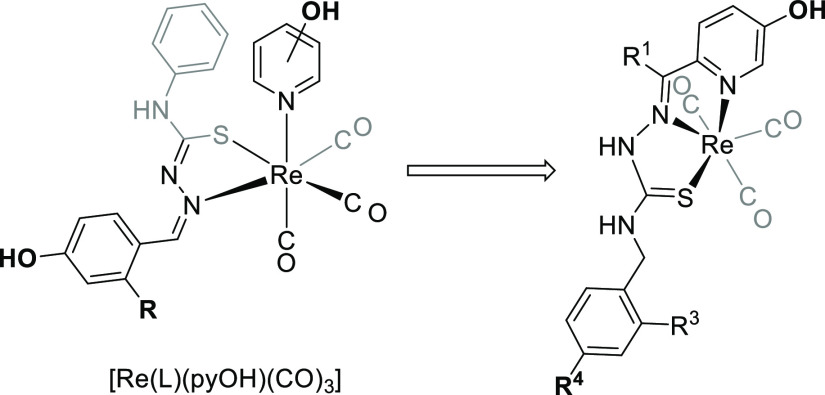
Representation of the Design of the Complexes Included in This Work

We have previously found that the complexes [ReX(HL)(CO)_3_], in which HL is a TSC with a phenol group on the C2^11^ or N1^11b^ positions, could undergo deprotonation of the ligand. This leads to the labilization of the halogen, and in the presence of 3- or 4-hydroxypyridine (pyOH), complexes of the type [Re(L)(pyOH)(CO)_3_] are obtained. These neutral thiosemicarbazonate complexes have a greater affinity for the estrogen receptor than any of the precursors^11^ but they do not have the necessary stability in aqueous media to obtain the corresponding ^99m^Tc derivative.^11b^ Given the information outlined above, we reasoned that the incorporation of a 2-pyridine group as an integral part of the TSC ligand (Scheme 2) would facilitate the formation and enhance the stability of the ^99m^Tc complex even in biological media.^10^ However, in preliminary experiments, it was found that the reaction with this type of ligand was problematic in terms of isolating the pure products.^9^

The work described here involved an investigation into the influence that the different substituents have on the stability of the complexes and the effect of different reaction media on the reaction product. In order to achieve these goals, attempts were made to obtain crystalline phases of some of the species formed with the aim of determining the path through which the reaction occurs. Whenever possible, sufficient quantities of the different compounds were isolated so that their spectroscopic characterization could be carried out.

## Results and Discussion

### Synthesis and Characterization of the TSC Ligands

The TSC ligands (Scheme 1) were synthesized by heating under reflux solutions of the previously obtained thiosemicarbazides (TSZ^n^)^12,13^ and 2-pyridinecarboxaldehyde (R^1^ = H) or the corresponding 2-acetylpyridine (R^1^ = CH_3_, R^2^ = H or OH). The products were obtained without the need for further purification after optimizing the solvent, the heating time, and the acidic medium (see Table S3 included in the Supporting Information for details).

The ligands were characterized by elemental analysis, MS-ESI^+^ spectrometry and IR spectroscopy, and also by X-ray diffraction for seven compounds. The hydrochloride compounds **[H**_**2**_**L**^**23**^**]Cl**, obtained from the reaction medium in the synthesis of the corresponding complex, and **[H**_**2**_**L**^**13**^**]Cl** resulting from the addition of HCl to a solution of the ligand, were also studied by X-ray diffraction.

The ^1^H NMR spectra of the ligands showed some differences, which suggests that the behavior in solution is more complicated than one would expect a priori. Thus, for example, the acetylpyridine derivatives (**HL**^**21–24**^, R^1^ = Me, R^2^ = H in Scheme 1) gave a single group of signals that is attributed to the presence of a single configuration around the azomethine bond C2=N3, which has the *E* configuration as characterized by the signal of the N2–H proton at around 10.50 ppm.^14^ For the sake of simplicity, the same notation, *Z* and *E*, will be used to identify both the type of isomer/configuration (e.g., on the C2–N3 link) and the rotamer/conformer (as derived from the substituents on the N2–C1 link) along the TSC/-ate arm C2–N3–N2–C1(S)–N1. In the aldehyde derivatives (R^1^ = H) the spectra obtained from DMSO-*d*_6_ solutions contain—in addition to signals similar to those discussed above—a set of signals with similar intensities that suggest the existence of other species in solution. This last group is characterized by a low-field signal (around 14 ppm although for **HL**^**12**^ they are significantly less intense). We attribute this group of signals to the *Z* configuration of the C2=N3 bond, which would allow the establishment of an N2–H···N4(py) hydrogen bond and, in turn, explain the strong shielding of this proton.^14^

It was found that modification of the synthesis conditions of **HL**^**13**^ allowed the isolation of materials in which either the *Z* or *E* configuration around the C2=N3 bond was predominant, with the other isomer occasionally appearing as an impurity. In fact, the X-ray diffraction study of the crystalline phase obtained from ethanol showed the presence of the *E*,*E*,*E*,*Z* isomer, whereas the isomer obtained from chloroform was *Z*,*E*,*E*,*Z* (vide infra).

**Figure 1:**
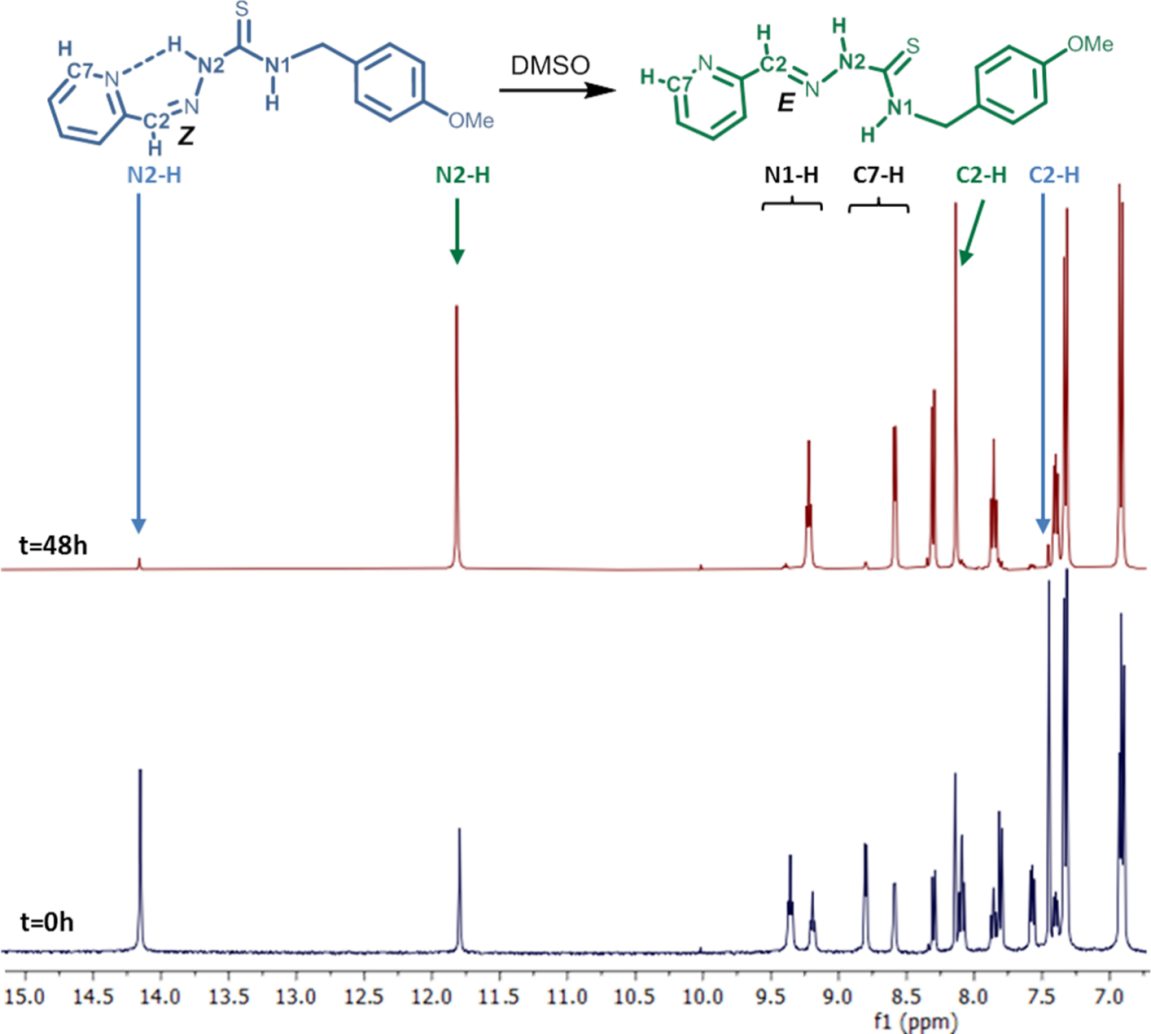
^1^H NMR spectra of a freshly prepared solution of **HL**^**13**^**(Z)** in DMSO-*d*_6_ and the same solution after 48 h.

The isolation of the pure phases of the two stereoisomers allowed the ^1^H NMR signals for the diastereoisomers to be assigned. Surprisingly, it was observed that the solutions of both diastereoisomers evolved at room temperature. The spectrum of the freshly prepared DMSO solutions of **HL**^**13**^**(Z)** contained signals corresponding to **HL**^**13**^**(E),** and the set of signals for the original compound disappeared almost completely after 48 h at room temperature (Figure 1). On the other hand, in the freshly prepared solution of **HL**^**13**^**(Z)** in MeOD-*d*_4_, it was observed that the predominant compound was already the **HL**^**13**^**(E)** isomer, although the signal of the original isomer was still present after several days at room temperature.

The opposite occurs when CHCl_3_ is used as a solvent, and **HL**^**13**^**(Z)** remains stable indefinitely while **HL**^**13**^**(E)** evolves toward the formation of the *Z* isomer. However, the addition of a small amount of MeOH-*d*_4_ to the solution promotes the conversion to the **HL**^**13**^**(E)** isomer, thus suggesting that the polarity and/or acidity of the solvent could play a role in the interconversion of isomers (see Supporting Information).

Most studies indicate that the isomerization barrier of the C2=N3 bond in TSCs requires an energy input that can only be overcome under certain conditions, such as metal coordination.^15^ There are several possible mechanisms for the conversion of this bond, although those involving tautomerization processes (uncatalyzed or acid-catalyzed) are considered to be more probable.^15,16^

In TSCs with aromatic heterocycles (substituted in C2), as is the case with 2-acetylpyridine TSC, the acid-catalyzed tautomerization mechanism has been proposed.^17^ More recently, Khalilian et al.^15^ identified the rate-limiting step in the conversion of this bond to 2-formylpyridine TSC in the zwitterionic form, which arises from the proton transfer of N2–H to the pyridine nitrogen N4. This process is likely to be significantly affected by the polarity/acidity of the solvent. We did not detect the presence of this species in the case of **HL**^**13**^ (although Nomiya et al.^14^ did detect this form in fully N1-substituted acetylpyridine TSC), but the effect produced by methanol on the chloroform solutions of **HL**^**13**^**(Z)** suggests that the degree of polarity of the medium (or hydrogen bond donors such as OH groups) in the mixture plays an important role. This hypothesis is reinforced by the observation of molecular association in the corresponding X-ray structures (see Supporting Information).

### Crystal and Molecular Structures of the Free Ligands

**Figure 2:**
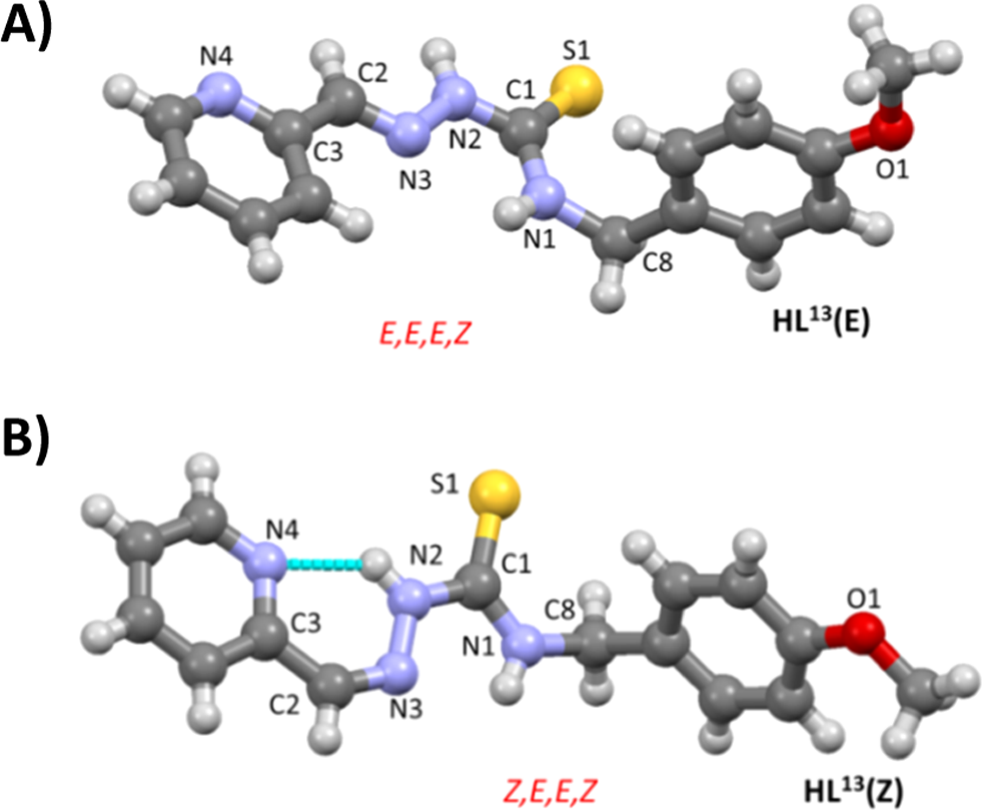
Representations of the molecular structures of the isomers *E* (A) and *Z* (B) of **HL**^**13**^.

The X-ray structures of the ligands **HL**^**11**^, **HL**^**12**^, **HL**^**22**^, **HL**^**23**^_**,**_ of the two isomers of **HL**^**13**^_**,**_ and the hydrochlorides of **HL**^**13**^ and **HL**^**23**^ were determined. For the sake of brevity, those of **HL**^**13**^ are discussed (Figure 2 and Table 1). A more complete discussion, including figures of the molecular structures and a selection of the main distances and angles for all of them, is included in the Supporting Information file.

**Table 1:** Selected Bond Lengths (Å) and Angles (°) for the Two Isomers of **HL**^**13**^ and Its Complexes

	**HL**^**13**^**(E)**	**HL**^**13**^**(Z)**	[ReCl(HL^13^)(CO)_3_]·(C_3_H_6_O)^a^	[ReCl(HL^13^)(CO)_3_]·1/2(CHCl_3_)^a^	[Re_2_(L^13^)_2_(CO)_6_]
X=			Cl(1)	Cl(1)	S(1)
S(1)–C(1)	1.676(1)	1.683(1)	1.699(3)	1.711(4)	1.791(4)
N(1)–C(1)	1.339(2)	1.331(2)	1.321(4)	1.327(4)	1.326(5)
N(2)–C(1)	1.354(2)	1.370(1)	1.356(4)	1.367(4)	1.306(5)
N(2)–N(3)	1.368(2)	1.368(1)	1.373(4)	1.371(4)	1.405(5)
N(3)–C(2)	1.276(2)	1.293(2)	1.305(4)	1.300(4)	1.304(5)
Re(1)–N(3)			2.187(2)	2.183(3)	2.180(3)
Re(1)–S(1)			2.462(0)	2.459(1)	2.482(1)
Re(1)–X			2.513(0)	2.518(1)	2.527(1)
N(1)–C(1)–N(2)	115.9(1)	115.9(1)	115.0(3)	114.9(4)	120.0(4)
N(1)–C(1)–S(1)	124.6(1)	125.39(9)	123.1(2)	122.1(3)	124.7(3)
N(2)–C(1)–S(1)	119.5(1)	118.70(9)	121.9(2)	122.8(3)	115.3(3)
N(3)–N(2)–C(1)	119.6(1)	118.8(1)	120.7(2)	118.0(4)	113.8(3)
N(3)–C(2)–C(3)	120.7(1)	130.5(1)	128.3(3)	128.7(4)	131.5(4)
C(2)–N(3)–N(2)	115.4(1)	118.3(1)	116.6(2)	117.1(4)	115.1(3)
N(3)–Re(1)–S(1)			79.1(8)	78.69(9)	76.66(9)
N(3)–Re(1)–X			83.5(8)	84.10(9)	81.9(1)
S(1)–Re(1)–X			87.3(3)	86.37(4)	81.48(3)

a Average values of the two molecules present in the asymmetric unit.

The bond distances and angles in the thiosemicarbazide fragment in both structures are consistent with some delocalization of the multiple bonds along the chain.^18,19^ However, the C1–S1, C2–N3, N2–N3, and N2–C1 distances suggest that, in spite of the delocalization, the canonical form depicted in Scheme 1 is predominant.

In the crystal obtained from methanol, **HL**^**13**^**(E)** (Figure 2A), the configuration of the C2=N3 bond is *E*. This is the predominant form observed in the rest of the TSC structures (except **HL**^**13**^**(Z)**), and it appears to be independent of the nature of substituents on the N1 and C2 atoms.

In the crystal obtained from chloroform, **HL**^**13**^**(Z)** shows the *Z* conformation for the formal double bond C2=N3. This conformation is probably favored by the presence of an intramolecular hydrogen bond involving the hydrazine group N2–H and the pyridine nitrogen N4 (Figure 2b). The values of the standard deviations do not allow differences to be discerned in the values of the bond distances in the TSC arm between the *E* and *Z* conformers.

### Synthesis of Rhenium(I) Complexes

The initial attempts for the reaction of TSC ligands with a pyridine fragment and [ReX(CO)_5_] or [ReX(CO)_5_(CH_3_CN)] yielded mixtures of several metalated species. The isolation of single crystals suitable for X-ray diffraction facilitated our understanding of the reactivity and allowed the design of synthetic routes that made it possible to obtain pure compounds.

**Scheme 3:**
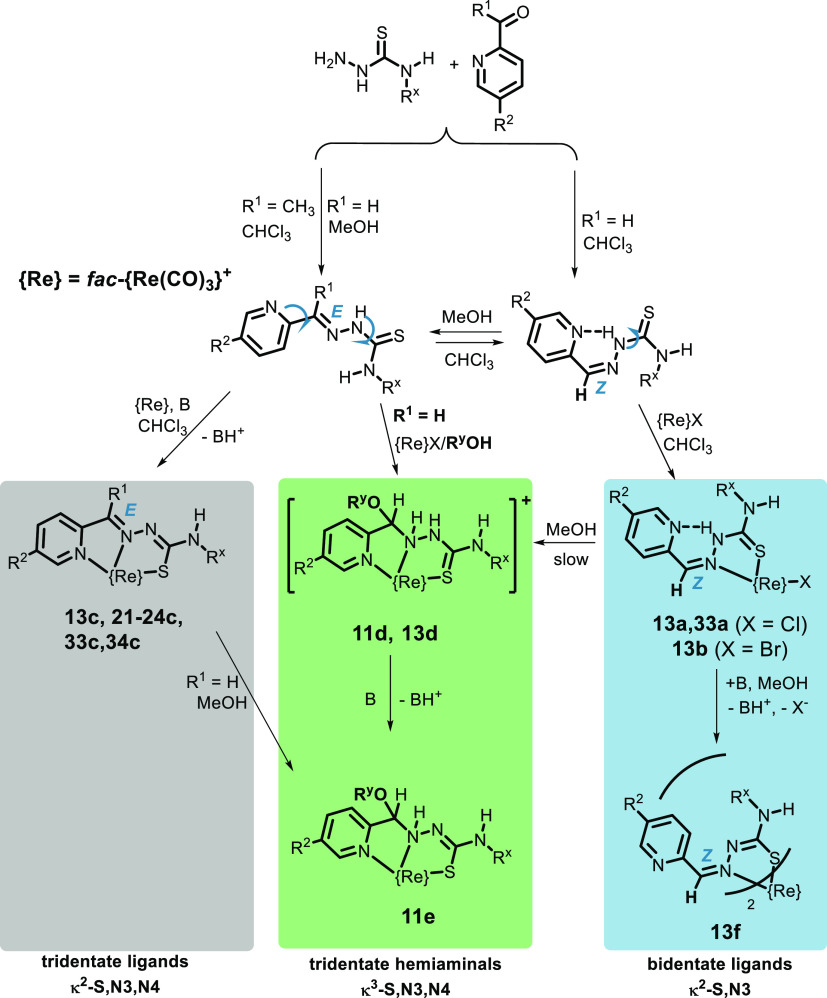
Summary of the Isolated Rhenium Complexes

These studies show that the nature of the resulting compounds strongly depends on the type of substituent on C2 (R^1^) and, to a significant extent, on the solvent used (Scheme 3).

### Reactions in the Absence of a Base. Formation of the Adducts [ReX(HL)(CO)_3_] (**13a,b**, **22a** and **33a**) and the Trinuclear Species **[Re**_**3**_**Cl**_**2**_**(L**^**23**^**)(HL**^**23**^**)(CO)**_**9**_**]**

The ligands reacted with [ReX(CH_3_CN)_2_(CO)_3_] in CHCl_3_ to form the adducts [ReX(HL)(CO)_3_] (Scheme 3), and some of these (derived from 2-formylpyridine, **HL**^**11–14**^ and **HL**^**33**^) could be isolated and characterized as pure compounds (**13a**,**b**, **22a,** and **33a**). In some of these compounds, where pyridine does not participate in coordination, a displacement of the signal corresponding to N2–H by more than 3 ppm was observed with respect to that in the free ligand. This change can be understood if we consider that this group, in addition to participating in the chelate ring of TSC, maintains the intramolecular hydrogen bond with the pyridine nitrogen. In fact, the X-ray structures of the chloroform and acetone solvates of **13a** show that the coordinated ligand retains the same conformation observed in **HL**^**13**^**(Z)** (vide infra). However, it should be noted that this kind of complex is obtained regardless of the ligand isomer used if the solvent is chloroform. A similar deshielding effect is observed for the N1–H and C2–H groups (bearing in mind the *Z* conformation of the C2=N3 link) upon coordination (around 1 ppm). The signals of the pyridine ring are also displaced to the lower field by 0.2–0.5 ppm, and the other aromatic ring signals remain relatively unchanged.

The reaction of the commonly used rhenium precursor [ReCl(CH_3_CN)_2_(CO)_3_] with a slight excess of the ligand **HL**^**23**^ (1:1.4) was investigated. The reaction mixture was heated under reflux in chloroform for 3 h, and this led to the formation of the first fraction of a crystalline material containing the hydrochloride **[H**_**2**_**L**^**23**^**]Cl**. After this material had been separated, a small second fraction of crystals of the trinuclear species **[Re**_**3**_**Cl**_**2**_**(L**^**23**^**)(HL**^**23**^**)(CO)**_**9**_**]** (vide infra) was formed. This complex contains deprotonated and neutral ligands, as evidenced by X-ray diffraction.

### Structures of the Acetone and Chloroform Solvates of [ReCl(HL^13^)(CO)_3_] (**13a**)

**Figure 3:**
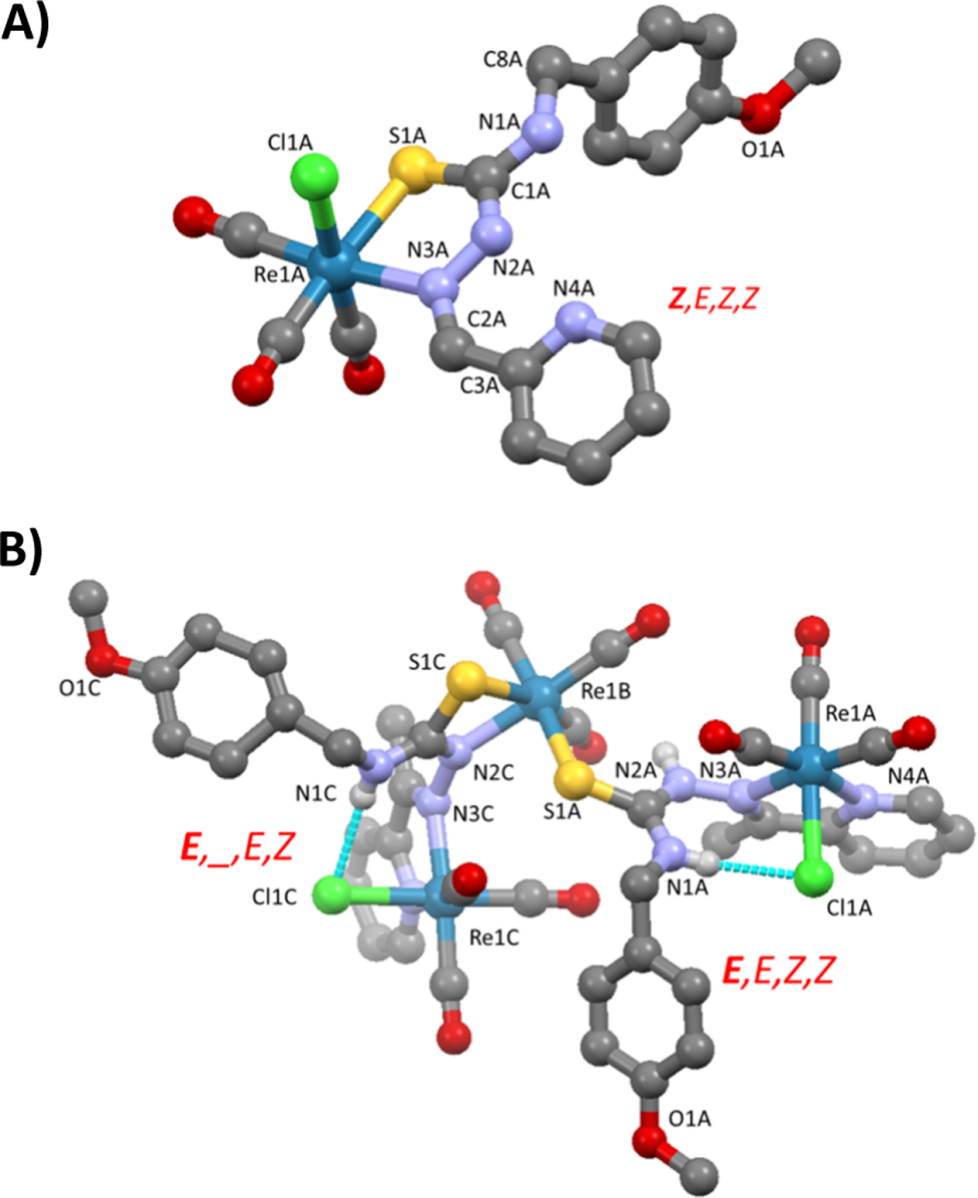
(A) One of the molecules of [ReCl(HL^13^)(CO)_3_] present in the asymmetric unit of the structure of **13a**. CHCl_3_ (**13a**) and (B) the structure of the trinuclear complex **[Re_3_Cl_2_(L^23^)(HL^23^)(CO)_9_]**·3(CHCl_3_) (solvent molecules and hydrogen atoms not involved in interactions are omitted for clarity).

The structures of the TSC complexes of **HL**^**13**^ were determined using single crystals obtained from aprotic solvents such as CHCl_3_ and acetone. Both structures contain two molecules in the asymmetric unit, and these molecules are associated by hydrogen bonds to form dimers (Figure S3). The average bond distances and angles for both molecules are included in Table 1. Nevertheless, the coordination mode of the ligand to rhenium is the same in both cases (Figure 3A), involving sulfur and azomethinic nitrogen N3 atoms to form a five-membered chelate ring. The coordination sphere around the metal is completed with the chloride and three carbonyl carbon atoms. These carbonyl ligands are in a facial configuration, and the geometry around the rhenium is octahedral, although the chelate bite imposes a slight distortion with respect to the ideal geometry. This bidentate coordination mode (κ^2^*S*,*N3*) is rather unusual in TSC ligands with additional coordinating groups^5^—taking more into account the known affinity of the fragment {Re/Tc(CO)_3_}^+^ for derivatives of pyridine.^8,20^ In fact, a bidentate κ^2^-*N3*,*N4* mode in a 2-pyridineformamide (R^1^ = NH_2_) TSC rhenium(I) complex has been reported previously.^9^ In the present case, the intramolecular H-bond N2–H···N4 probably plays a stabilizing role in the structure by blocking the coordinative capacity of the pyridine fragment and maintaining the planarity of the TSC arm in the structure. The Re–S and Re–N bond distances are within the range observed previously for TSC complexes with rhenium(I).^5,9,19,21−25^

### Structure of the Trinuclear Complex **[Re**_**3**_**Cl**_**2**_**(L**^**23**^**)(HL**^**23**^**)(CO)**_**9**_**]**

The main interatomic distances and angles are included in Table 2. The structure presents some interesting features: it consists of a neutral TSC complex {ReCl(HL^23^)(CO)_3_} (labeled with the letter A in Figure 3B) and a thiosemicarbazonate {ReCl(L^23^)(CO)_3_}^−^ (labeled with the letter C in Figure 3B), where the **L**^**23(−)**^ or **HL**^**23**^ ligands bind to {ReCl(CO)_3_} fragments to form chelate rings by coordination of the N4 and N3 nitrogen atoms. This situation is similar to the arrangement observed in the previously reported complex [ReBr(HL^NH_2_^)(CO)_3_] (HL^NH_2_^ = 2-pyridineformamide TSC).^9^ In both cases, it is also possible to observe an intramolecular interaction involving the N1–H group and the Cl^–^ ligand of the same unit, which probably plays an important role in the stability of the trinuclear complex. The thiosemicarbazonate ligand binds to a third {*fac*-Re(CO)_3_}^+^ (atoms labeled with the letter B in Figure 3B) fragment through the S1 and N2 atoms to form a four-membered ring, while this metal center is also coordinated to the TSC ligand but only by the sulfur atom. In the thiosemicarbazonate molecule (the one that forms the four-membered chelate ring, identified with the letter C in Figure 3B), the thiosemicarbazide arm loses planarity so that the two chelate rings are practically orthogonal (the angle between mean planes is 77.23°). As a result, we do not consider the use of conformation descriptors useful in this case. The two resulting polyhedra, ReS_2_NC_3_ and ReClN_2_C_3_, retain the facial configuration of the three carbonyl groups in a distorted octahedral geometry, mainly around Re1B, which contains the four-membered chelate ring.

**Table 2:** Selected Bond Lengths (Å) and Angles (°) for **[Re**_**3**_**Cl**_**2**_**(L**^**23**^**)(HL**^**23**^**)(CO)**_**9**_**]**·3(CHCl_3_)

Re(1A)–N(4A)	2.166(6)	Re(1B)–N(2B)	2.180(7)
Re(1A)–N(3A)	2.174(6)	Re(1C)–N(4B)	2.154(9)
Re(1A)–Cl(1A)	2.503(2)	Re(1C)–N(3B)	2.160(7)
Re(1B)–S(1A)	2.495(2)	Re(1C)–Cl(1C)	2.488(3)
Re(1B)–S(1B)	2.565(3)		
S(1A)–C(1A)	1.712(8)	S(1B)–C(1B)	1.75(1)
N(1A)–C(1A)	1.314(9)	N(1B)–C(1B)	1.33(1)
N(2A)–C(1A)	1.360(9)	N(2B)–C(1B)	1.32(1)
N(3A)–N(2A)	1.406(9)	N(3B)–N(2B)	1.42(1)
N(3A)–C(2A)	1.29(1)	N(3B)–C(2B)	1.30(1)
N(4A)–Re(1A)–N(3A)	73.9(2)	C(1A)–N(2A)–N(3A)	121.2(6)
N(4A)–Re(1A)–Cl(1A)	81.0(2)	N(1A)–C(1A)–N(2A)	117.8(7)
N(3A)–Re(1A)–Cl(1A)	85.0(2)	N(1A)–C(1A)–S(1A)	121.8(6)
N(2B)–Re(1B)–S(1A)	82.4(2)	N(2A)–C(1A)–S(1A)	120.4(6)
N(2B)–Re(1B)–S(1B)	63.6(2)	C(1B)–N(2B)–N(3B)	118.9(7)
S(1A)–Re(1B)–S(1B)	78.71(8)	N(3A)–C(2A)–C(3A)	114.6(7)
N(4B)–Re(1C)–N(3B)	73.6(3)	N(3A)–C(2A)–C(16A)	124.0(7)
N(4B)–Re(1C)–Cl(1C)	82.6(3)	N(2B)–C(1B)–N(1B)	126.1(9)
N(3B)–Re(1C)–Cl(1C)	85.1(2)	N(2B)–C(1B)–S(1B)	109.4(6)
C(2A)–N(3A)–N(2A)	116.7(6)	N(1B)–C(1B)–S(1B)	124.5(8)

### Reactions in the Presence of a Base. Formation of the Tridentate Thiosemicarbazonate Complexes [Re(L)(CO)_3_] (**13c**, **21c–24c**, **33c** and **34c**)

The addition of a base (usually NEt_3_) to the reaction medium used to obtain the adducts [ReCl(HL)(CO)_3_] led to the desired thiosemicarbazonate complexes [Re(L)(CO)_3_] in variable yields (35–70%) depending on the ligand (Scheme 3). The solvent also limits the reaction since, in the case of aldehyde derivatives (R^1^ = H), this metal compound was not obtained using MeOH or EtOH (vide infra). As described before,^9^ ketone derivatives (R^1^ = CH_3_) do form such complexes when alcohols are used in the synthesis, but the yield is usually low. The formation of this type of compound was confirmed by elemental analysis and mass spectra by the absence of the halide ligand and by the ^1^H NMR spectra due to the disappearance of the signal, corresponding to the N2–H proton, along with shielding of the signal, corresponding to C2–H in the derivatives with R^1^ = H.

Unfortunately, it was not possible to obtain single crystals of these compounds, but the data for the complex published previously (R^1^ = CH_3_, R^2^ = R^3^ = R^4^ = H, *n* = 0 according to Scheme 1) suggest that the adoption of this coordination imposes significant structural stress^9^ that probably explains the reactivity observed for this type of complex.

**Scheme 4:**
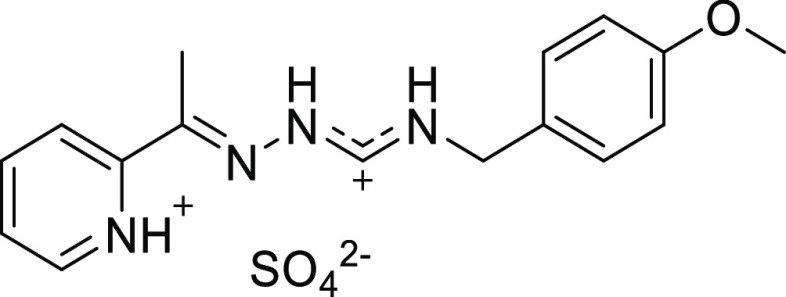
Formamidrazone Isolated from the Reaction of **HL**^**23**^ and [ReCl(CH_3_CN)_2_(CO)_3_]

The presence of excess ligand in the reaction also yielded the desired complex but was accompanied by the formation of by-products. This was the case in the reaction of [ReCl(CH_3_CN)_2_(CO)_3_] with excess of **HL**^**23**^ (1:4) in chloroform, where after isolation of complex **23c,** a small fraction of formamidrazone crystals was isolated as a consequence of desulfurization of the TSC (Scheme 4). This process has previously been observed in free^26^ and complexed^27^ TSCs. Structure determination data obtained by X-ray diffraction of the last compound are included in the Supporting Information but will not be discussed here.

### Influence of the Solvent in the Formation of Complexes: Reactions in MeOH and/or EtOH

Chloroform is not the best solvent for the design of reaction media compatible with biological systems, so other solvents with higher polarity, such as EtOH and MeOH, were used for the above reaction. In this case, the tridentate thiosemicarbazonate compounds were obtained, as mentioned before, when the ligand was a ketone derivative (R^1^ = CH_3_). However, a completely different type of compound was isolated when R^1^ = H, as shown by spectroscopic studies and X-ray diffraction of the crystalline samples. The new compounds result from the addition of the alcohol group to the C2=N3 bond to produce the formal reduction of that double bond (Scheme 3) and the formation of the corresponding hemiaminal (the term N,O-aminal has also been used).

The new (hemiaminal) ligands form cationic complexes [Re(HL^ORy^)(CO)_3_]X by tridentate coordination to the rhenium after displacement of the halide, which remains as a counteranion. This complex may undergo deprotonation if a base such as NEt_3_ is added to form the neutral complex [Re(L^ORy^)(CO)_3_] (only with methanol, Ry = Me, could this kind of compound be isolated).

The formation of both types of complexes can be detected by NMR spectroscopy based on the disappearance of the azomethine R–C(2)–H signal (around 8.4 ppm), which now becomes an R–(RO)C2(H)– aliphatic group with signals between 5.4 and 6.0 ppm. Furthermore, the N3–H proton signals appear in the range of 9–10 ppm.

The tridentate thiosemicarbazonate compound [ReCl(L^13^)(CO)_3_] (**13c**) also underwent the addition of MeOH when the solution was heated under reflux for 5 h. In this case, the yield was almost stoichiometric. However, the formation of the hemiaminal was not detected when the same experimental conditions were used with tridentate thiosemicarbazonate complexes of acetone derivatives (R^1^ = CH_3_), such as **23c**.

Hemiaminal is a functional group found in several natural products and pharmaceuticals,^28^ and it is used in synthesis to generate in situ reactive imines for specific reactions.^29^ These compounds are also considered intermediates in the formation of imines from amines and carbonyls, so it cannot be ruled out that their isolation can be considered as the intermediate step in the rupture of the C2=N3 bond observed in some TSC^9^ and hydrazone^30^ complexes.

**Figure 4:**
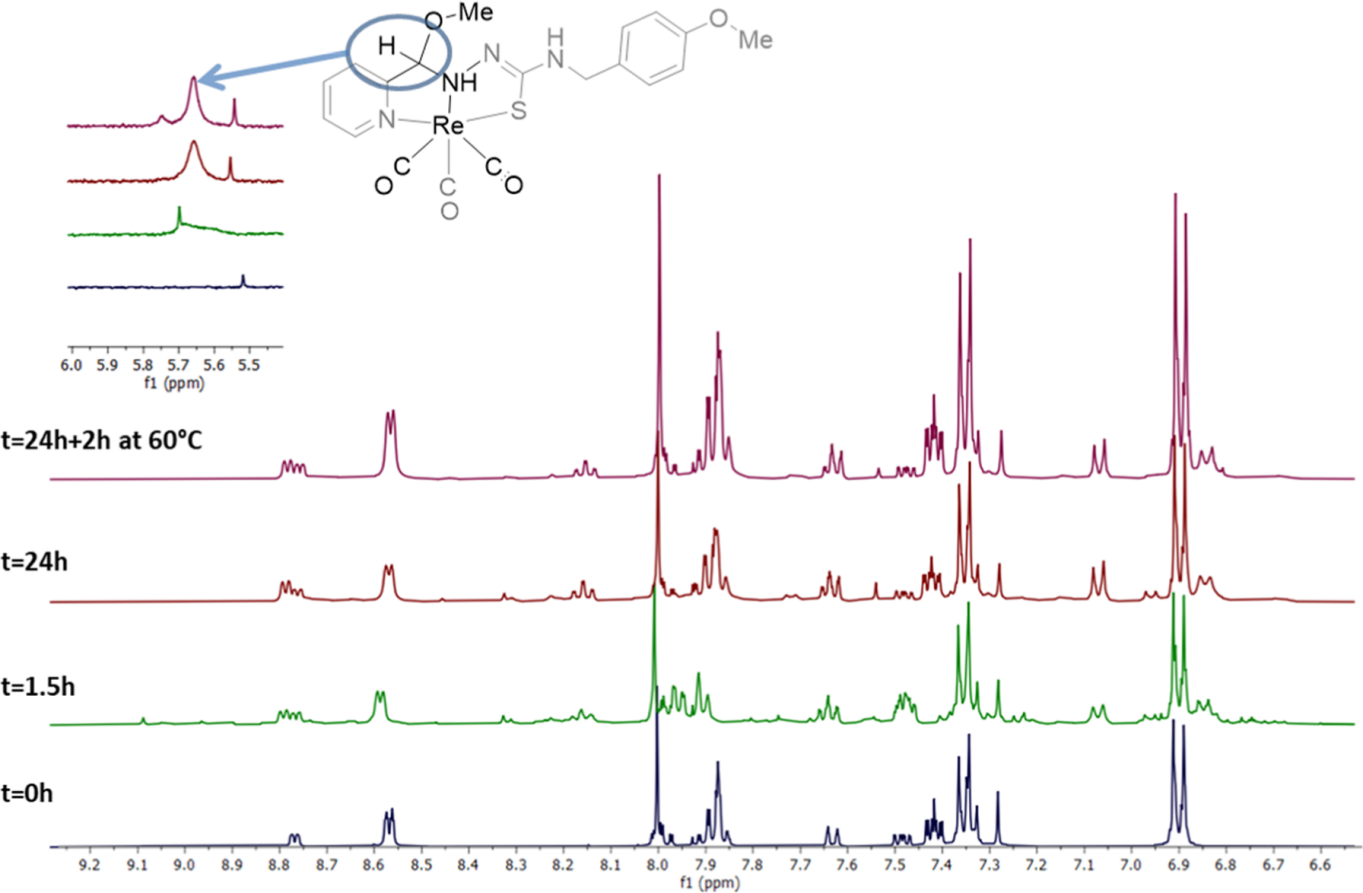
Monitoring of the reaction of **HL**^**13**^ and [ReCl(CH_3_CN)_2_(CO)_3_] in MeOH-*d*_4_ (see text for details).

In an effort to study the process in greater depth, the reaction in MeOH-*d*_4_ with an excess of **HL**^**13**^ and [ReCl(CH_3_CN)_2_(CO)_3_] was performed and monitored by ^1^H NMR spectroscopy. After the preparation and storage of the solution at room temperature for 90 min, it was observed that the reaction had already started because the characteristic signal of the azomethinic group R(H)C2=N3– in the ^1^H NMR spectrum at 8.2 ppm decreased in intensity and a new set of signals due to the R(RO)HC2–N3H–R group appeared at around 5.6 ppm (Figure 4). After 24 h at room temperature, the equilibrium condition appeared to have been reached. The sample was then heated to 60 °C for 2 h, but the spectrum did not change significantly.

### Structures of the Hemiaminal Complexes

**Figure 5:**
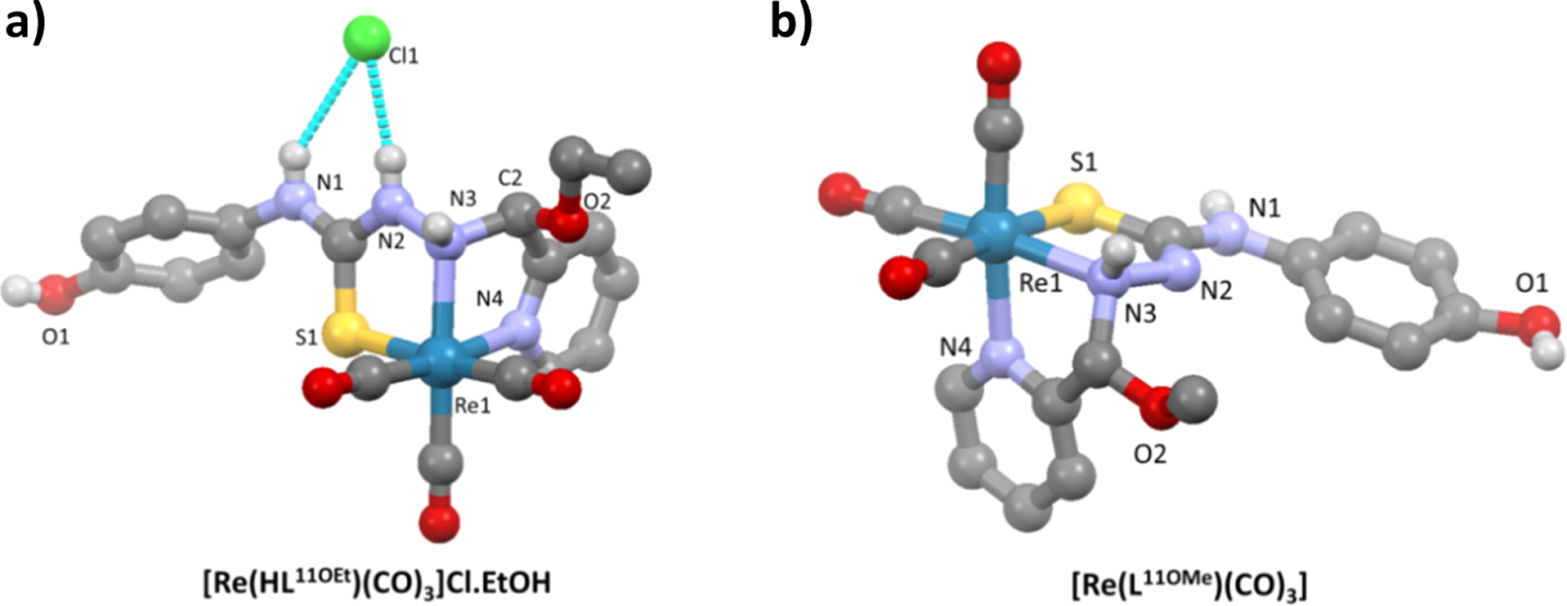
Structures of [Re(HL^11OEt^)(CO)_3_]Cl·(EtOH) (a) and [Re(L^11OMe^)(CO)_3_] (b) (solvent molecules and hydrogen atoms not involved in interactions are omitted for clarity).

The structures of the cationic complexes [Re(HL^11OEt^)(CO)_3_]Cl·(EtOH), **11d** (Figure 5a) and [Re(HL^13OEt^)(CO)_3_]Br·1/2H_2_O, **13d** (Figure S4) confirm the formation of the hemiaminal involving the addition of an ethanol molecule at the C2=N3 bond. In both complexes, the ligand acts in a tridentate manner by the coordination of the S,N3 and the pyridine nitrogen N4 to form two five-membered chelate rings. The five-membered chelate ring formed by the coordination of N4 (pyridine) and N3 (amine) atoms is not planar, and the configuration is *facial*.

In the complexes, there are two chiral centers in addition to the metal center itself, and these are the reduced double bond atoms C2 and N3. Although all crystals are centrosymmetric, consequently both enantiomers are present.

The formation of the hemiaminal involves the formal reduction of the C2–N3 bond, and, as can be seen in the three structures, this distance has values of around 1.50 Å (Table 3). Therefore, the ligand is no longer flat, and this imparts greater flexibility to the molecule for “*fac*” coordination. In addition, the ligand does not appear to require prior deprotonation to achieve tridentate denticity.

**Table 3:** Selected Bond Lengths (Å) and Angles (°) for the Hemiaminal Complexes

	[Re(HL^11OEt^)(CO)_3_]Cl·(EtOH)	[Re(HL^13OEt^)(CO)_3_]Br·1/2(H2O)	[Re(L^13OMe^)(CO)_3_]
S(1)–C(1)	1.702(2)	1.694(5)	1.763(4)
N(1)–C(1)	1.334(3)	1.329(6)	1.342(6)
N(2)–C(1)	1.336(3)	1.347(6)	1.297(5)
N(2)–N(3)	1.422(3)	1.432(5)	1.467(5)
N(3)–C(2)	1.512(3)	1.527(6)	1.510(6)
Re(1)–N(3)	2.209(2)	2.205(4)	2.219(4)
Re(1)–S(1)	2.4608(6)	2.446(1)	2.441(1)
Re(1)–N(4)	2.186(2)	2.194(3)	2.193(4)
N(1)–C(1)–N(2)	115.2(2)	114.9(4)	120.1(4)
N(1)–C(1)–S(1)	122.1(2)	121.9(4)	126.8(4)
N(2)–C(1)–S(1)	122.6(2)	123.1(3)	113.1(3)
N(3)–N(2)–C(1)	122.8(2)	121.3(4)	115.5(4)
N(3)–C(2)–C(3)	108.4(2)	105.2(3)	108.6(4)
C(2)–N(3)–N(2)	107.8(2)	107.2(3)	108.8(3)
N(3)–Re(1)–S(1)	80.94(5)	81.2(1)	79.5(1)
N(3)–Re(1)–N(4)	75.01(7)	73.3(1)	74.9(2)
S(1)–Re(1)–N(4)	86.89(5)	83.8(1)	84.2(1)

A comparison of the present structures with those of previously published tridentate 2-acetylpyridine thiosemicarbazonates^9^ shows that the Re–N3 distance is longer and the Re–S1 is shorter in the three complexes of the hemiaminal derivatives. Furthermore, while the idealized planes of the two chelate rings formed in the hemiaminal derivatives are orthogonal (the angles between the planes defined by the chelate rings are in the range 85–90°), in the thiosemicarbazonate derivative, this angle is almost 120°, probably due to the higher rigidity of the thiosemicarbazonate ligand associated with the presence of the C2=N3 double bond.

With respect to the coordinated ligand, there are not significant differences in the C1–S1 distances in neutral ligands (cationic complexes **11d** and **13d**). However, when the ligand is deprotonated, for example, in **13e** (Figure 5b), the C1–S1 distance is longer than in **13d**. In addition, the reduction of the C2=N3 bond of the TSC ligand limits the possibility for conjugation of the multiple bond in the S1–C1–N2–N3 chain of the hemiaminal ligand, so in these structures, the N2–N3 distance clearly falls within the expected range for a single bond.

### Influence of the Solvent on the Stability of the Complexes

In view of the ease with which tridentate thiosemicarbazonate complexes of aldehyde derivatives (R^1^ = H) form the corresponding hemiaminal, it was decided to determine whether this transformation is also possible in the case of bidentate TSC adducts [ReX(HL)(CO)_3_]. For this purpose, a solution of **13a** in methanol was heated under reflux for 5 h. Approximately 50% of the pure starting complex was recovered in the first precipitate. However, the filtrate residue contained only small amounts of hemiaminal, which shows that the transformation reaction in this case seems to be quite slow. Given the structure of bidentate TSC complexes, where the intramolecular hydrogen bond N2–H···N4 complicates the N4 pyridine nitrogen coordination capacity (vide supra), we reasoned that the addition of a base, such as NEt_3_, should break this hydrogen bond. This should in turn promote the formation of tridentate ligands and, consequently, accelerate the reaction of the TSC-coordinated ligand toward hemiaminal formation.

**Figure 6:**
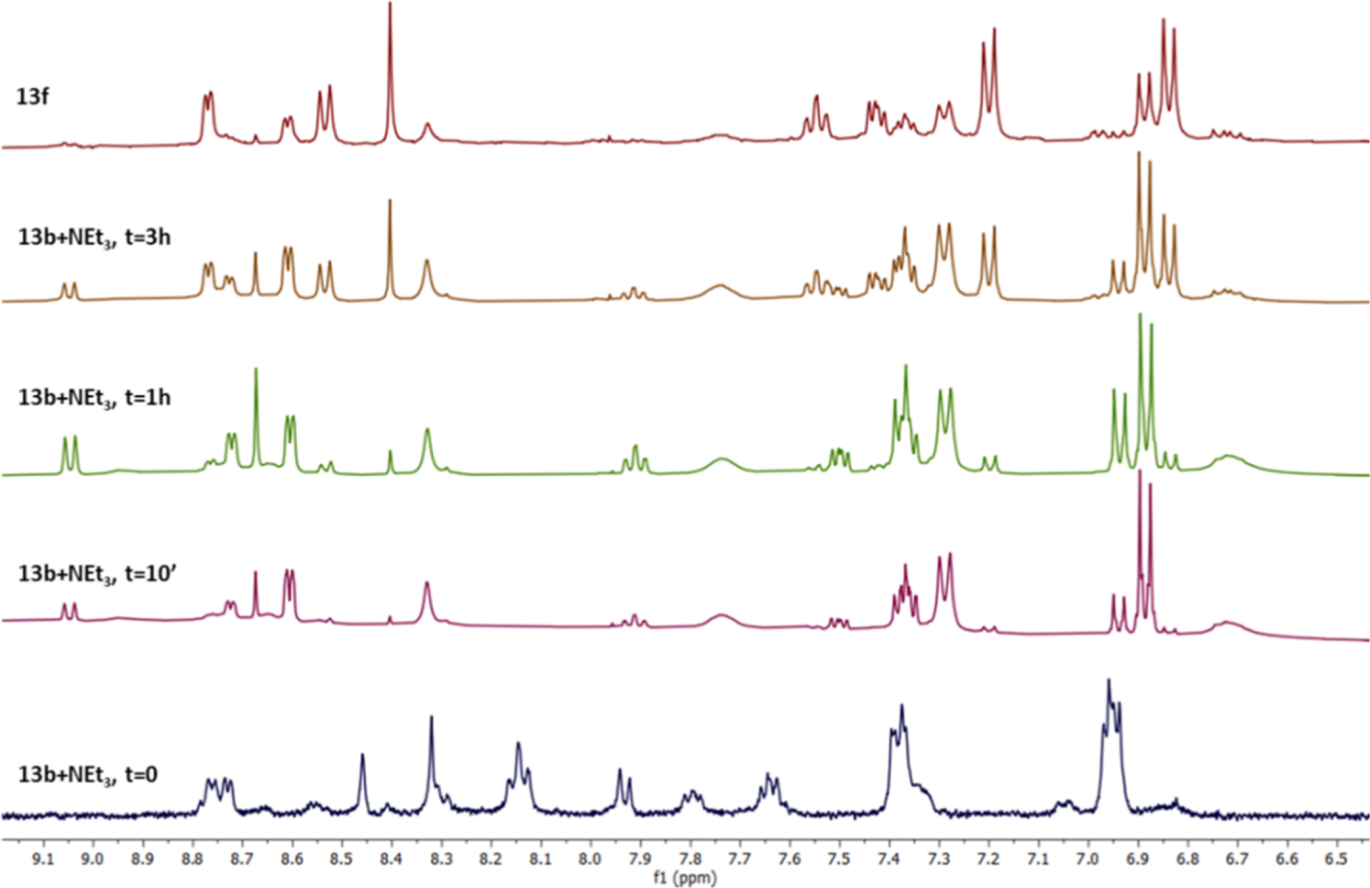
^1^H NMR monitoring of the addition of NEt_3_ to a MeOH-*d*_4_ solution of **13b**. The spectrum of the single crystal solutions of dimer **13f** is included for comparison (top).

It was decided to monitor this reaction in an NMR tube at room temperature (Figure 6) and, in clear contrast to the result of the reaction of the **HL**^**13**^ ligand and the acceptor [ReCl(CH_3_CN)_2_(CO)_3_] in methanol, after 3 h at room temperature, signals due to hemiaminal formation were not observed (Figure 6). After storing this sample overnight at room temperature, single crystals could be isolated, and X-ray diffraction showed the formation of the dimeric thiosemicarbazonate [Re_2_(L^13^)_2_(CO)_6_] (**13f**) (Scheme 3). It is worth noting that the reaction involving the addition of base to chloroform led to the isolation of the tridentate thiosemicarbazonate complexes (vide supra). However, the formation of this type of dimer is not surprising, and the spontaneous formation of such species has been observed numerous times for TSCs,^8,19,24,25^ either in the absence of the base or by forcing its formation by introducing NaOH into the medium. The tridentate capacity of the ligand is not an impediment to the formation of such dimers, as evidenced by TSCs derived from 4,6-diacetylresorcinol,^5^ and their formation is probably favored by the low solubility of the dimers, which facilitates the separation of the complex from the reaction medium, in this case, MeOH. Previous studies on these kinds of compounds in solution in DMSO suggest that dissociation occurs with the probable formation of the solvent complex [Re(solv)(L)(CO)_3_]. However, it is also probable that the reorganization of the thiosemicarbazonate ligand may be difficult, especially for the groups involved in coordination.^31^

### Structure of the Dinuclear Thiosemicarbazonate [Re_2_(L^13^)_2_(CO)_6_] (**13f**)

**Figure 7:**
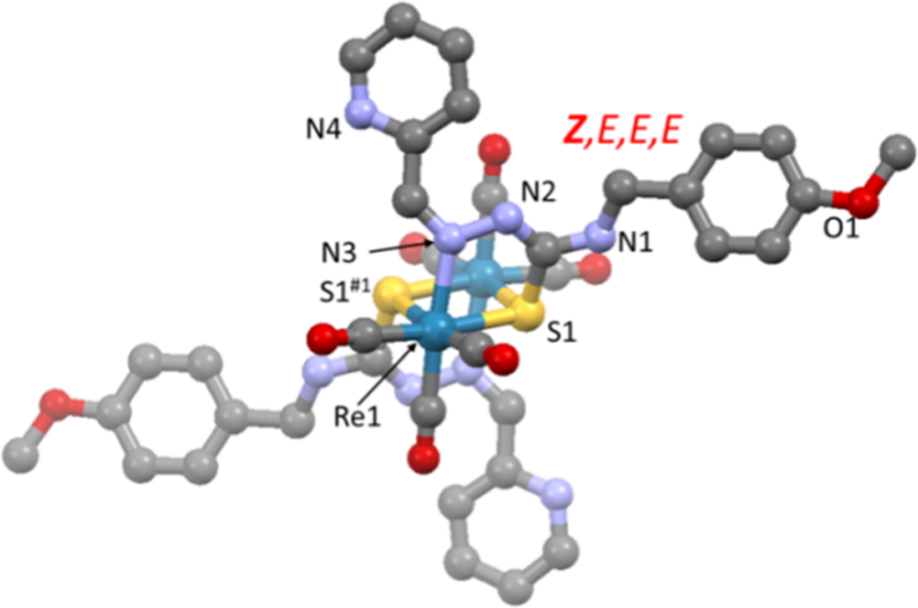
Molecular structure of the dinuclear [Re_2_(L^13^)_2_(CO)_6_]) (**13f**), where hydrogen atoms are omitted for clarity.

The structure of the complex **13f** is represented in Figure 7 and it consists of a dimer [Re_2_(L^13^)_2_(CO)_6_] in which the ligand, in addition to deprotonation, establishes a sulfur bridge between the rhenium centers. This bridge is not symmetrical, but the center of the Re_2_S_2_ diamond is located on an inversion center. The thiosemicarbazonate ligand maintains the *Z*,*E*,*E*,*E* conformation observed in the precursor as well as the κ^2^*S1*,*N3* coordination bidentate mode (vide supra). In fact, the structure can be considered as derived from the rotation around the C2–C3 bond (formally a single bond) of the pyridine ring so that N4 now avoids the deprotonated N2 group.

Deprotonation reinforces the effects normally observed upon metal coordination in these types of ligands, that is, the C1–S1 distance becomes longer, whereas the C1–N2 distance is shorter in TSCs with respect to both the free ligand and **13a**. Other, less marked, changes in the N2–N3 and C2–N3 distances are also consistent with the major thiolate character of the ligand due to its deprotonation (Table 1).

It is interesting to note that although the pyridine group does not coordinate with the metal, it does not appear to establish any other relevant secondary interaction.

## Conclusions

The aim of the present work was to identify the optimal conditions to obtain, in acceptable yields, complexes of tridentate thiosemicarbazonate ligands derived from 2-pyridine-*S*,*N3*,*N*_*py*_. In previous experiments, it was found that despite the apparent suitability of the ligand for this purpose, the products formed were very varied and usually difficult to identify. In this sense, it was planned to study a relatively broad group of ligands to investigate the role that different substituents have on reactivity. The effects of the reaction solvent and reaction temperature were also evaluated. In general, the predominance of one or other product has a strong dependence on the R^1^ substituent, that is, whether the TSC is derived from an aldehyde (R^1^ = H) or a ketone (R^2^ = CH_3_ in the examples included in this article). For aldehyde derivatives, the nature of the reaction solvent is also important because the target complexes are not stable in MeOH or EtOH. It was found that in these reactions, reduction of the C2=N3 bond occurs by the addition of a molecule of either solvent, followed by the formation of the hemiaminal group. This reaction was not observed for ketone derivatives (R^1^ = CH_3_).

In the absence of the base and in chloroform, the major product was the bidentate TSC-*S*,*N3* complex. In the same medium, the addition of base allowed the isolation of the tridentate thiosemicarbazonate complex (in fact, this was the only way in which these compounds could be isolated for aldehyde derivatives). On heating the mixture under reflux in methanol, only a small amount of the hemiaminal complex was detected, and the addition of a base led to the crystallization of the dimeric complex of the bidentate TSC ligand.

In a recent article, we showed that rhenium(I) is particularly suitable for stabilizing possible intermediates in the formation of TSC/ate ligand complexes. In most of the complexes of the metals of the main and transition groups, these intermediates are quickly transformed into the thermodynamic product. This is particularly prevalent for inert centers (and the systems in which a metal of the second and third transition series has a d^6^ configuration). However, the conversion between several of these products may be kinetically hindered. This finding is more relevant as the formation time of the complex becomes more limited, as is the case with the synthesis of radiopharmaceuticals in general and ^99m^Tc in particular.

## Experimental Section

The starting materials and solvents were obtained commercially and used as supplied. The acetonitrile complexes [ReX(CH_3_CN)_2_(CO)_3_] (X = Cl, Br) were synthesized^32^ from the corresponding [ReX(CO)_5_] (X = Cl and Br)^33^ obtained from commercial [Re_2_(CO)_10_]. Elemental analyses (carbon, hydrogen, nitrogen, and sulfur) were carried out on a FlashEA 1112 Series microanalyzer. IR spectra were recorded in the solid phase by attenuated total reflectance (ATR; 4000–400 cm^–1^) on a Jasco FT/IR-6100 spectrophotometer. ^1^H NMR spectra were obtained on a Bruker AMX 400 spectrometer. Mass spectrometry (MS) (positive-ion ESI) was carried out on a microTOF-Focus (Bruker Daltonics) mass spectrometer.

### Crystallography

The crystallographic data were collected at 100 K using a Bruker D8 Venture diffractometer with a Photon 100 CMOS detector and Mo Kα radiation (λ = 0.71073 Å) generated by an Incoatec high-brillance microfocus source equipped with Incoatec Helios multilayer optics. The software APEX3^34^ was used to collect frames of data, index reflections, and determine the lattice parameters. SAINT was used for the integration of the intensity of reflections and SADABS for scaling and empirical absorption correction.^35^ The structures were solved by direct methods by using the program SHELXT.^36^ All non-hydrogen atoms were refined on F^2^ with anisotropic thermal parameters by using SHELXL.^37^ Hydrogen atoms were inserted at calculated positions and refined as riding atoms. The graphics were produced with MERCURY.^38^ The crystallographic data collection and refinement parameters are listed in Table S1.

### Synthesis

Ligands included in Scheme 1 were synthesized by the reaction of the aldehyde or ketone as described before.^31^ The details and spectroscopic characterization are included in the Supporting Information.

### Synthesis of [ReX(HL^*n*^)(CO)_3_] (**13a**, *n* = 13, X = Cl; **13b**, *n* = 13, X = Br; **22a**, *n* = 22, X = Cl; **33a**, *n* = 33, X = Cl)

An ethanol or chloroform solution of equimolar amounts of the rhenium(I) precursor (*fac*-[ReCl(CH_3_CN)_2_(CO)_3_]/[ReBr(CO)_5_]) and **HL**^**13**^ was heated under reflux for 1–6 h. The resulting solution was concentrated in vacuum to half of its initial volume and stored at 4 °C after adding diethyl ether. The resulting solid was filtered off and vacuum dried over CaCl_2_/KOH. Reagent quantities and synthesis conditions are collected in the Supporting Information.

**13a**: Yield: 29 mg (29%). mp 208 °C. C_18_H_16_ClN_4_O_4_ReS (606.0): Calcd C, 35.6; H, 2.7; N, 9.2; S, 5.3. Found: C, 35.2; H, 2.6; N, 9.0; S, 5.0%. MS-ESI [*m*/*z* (%)]: 571 (80) |M – Cl|^+^, 612 (100) |M – Cl + CH_3_CN|^+^. IR data (ATR, ν/cm^–1^): 3171b ν(NH, OH); 2012s, 1908s, 1876vs ν(C=O_fac_); 1547m, 1514m ν(C=N); 1030s ν(O–CH_3_); 768m ν(C=S).

^1^H NMR (400 MHz, DMSO-*d*_6_, ppm): 10.50 (*t*, ^3^*J* = 5.7 Hz, 1H, N1H), 8.75 (*d*, ^3^*J* = 5.2 Hz, 1H, C7H), 8.35 (*s*, 1H, C2H), 8.30 (*td*, ^3^*J* = 7.7 Hz, ^3^*J* = 6.2 Hz, 1H, C5H), 8.25 (*d*, ^3^*J* = 7.9 Hz, 1H, C4H), 7.79 (*t*, ^3^*J* = 6.4 Hz, 1H, C6H), 7.36 (*d*, ^3^*J* = 8.2 Hz, 2H, C10H, C14H), 6.96 (*d*, ^3^*J* = 8.2 Hz, 2H, C11H, C13H), 4.71 (*dd*, ^2^*J* = 14.8 Hz, ^3^*J* = 5.5 Hz, 1H, C8aH), 4.66 (*dd*, ^2^*J* = 14.8 Hz, ^3^*J* = 5.5 Hz, 1H, C8bH), 3.76 (*s*, 3H, C16H).

**13b·(CH**_**3**_**CH**_**2**_**OH)**: Yield: 48 mg (34%). mp 194 °C. C_18_H_16_BrN_4_O_4_ReS·(CH_3_CH_2_OH) (696.0): Calcd C, 34.5; H, 3.2; N, 8.1; S, 4.6. Found: C, 34.0; H, 2.8; N, 8.3; S, 4.2%. MS-ESI [*m*/*z* (%)]: 571 (100) |M – Br|^+^, 612 (70) |M – Br + CH_3_CN|^+^. IR data (ATR, ν/cm^–1^): 3187b ν(NH, OH); 2017s, 1913s, 1883vs ν(C=O_fac_); 1551m, 1512m ν(C=N); 1026s ν(O–CH_3_); 759m ν(C=S).

^1^H NMR (400 MHz, DMSO-*d*_6_, ppm): 17.15 (*br*, 1H, N2H), 10.46 (*t*, ^3^*J* = 6.0 Hz, 1H, N1H), 8.75 (*d*, ^3^*J* = 4.9 Hz, 1H, C7H), 8.37 (*s*, 1H, C2H), 8.32 (*t*, ^3^*J* = 7.5 Hz, 1H, C5H), 8.25 (*d*, ^3^*J* = 7.6 Hz, 1H, C4H), 7.79 (*t*, ^3^*J* = 6.0 Hz, 1H, C6H), 7.37 (*d*, ^3^*J* = 8.4 Hz, 2H, C10H, C14H), 6.96 (*d*, ^3^*J* = 8.4 Hz, 2H, C11H, C13H), 4.72 (dd, ^2^*J* = 15.4 Hz, ^3^*J* = 6.0 Hz, 1H, C8aH), 4.67 (*d*, ^2^*J* = 15.4 Hz, ^3^*J* = 6.0 Hz, 1H, C8bH), 3.76 (*s*, 3H, C16H).

**22a·1/2CHCl**_**3**_: Yield: 70 mg (87%). *T*_dec_.: 177 °C. C_17_H_13_FClN_4_O_4_ReS·1/2CHCl_3_ (668.9): Calcd C, 31.4; H, 2.0; N, 8.4; S, 4.7. Found: C, 31.7; H, 2.3; N, 8.6; S, 4.3%. MS-ESI [*m*/*z* (%)]: 575 (99) |M – Cl|^+^, 616 (100) |M – Cl + CH_3_CN|^+^. IR data (ATR, ν/cm^–1^): 3141b ν(NH, OH); 2022s, 1886vs ν(C=O_fac_); 1542m, 1515s ν(C=N); 1299m ν(C–OH); 751s ν(C=S).

^1^H NMR (400 MHz, CD_3_OD-*d*_4_, ppm): 9.07 (*d*, ^3^*J* = 5.5 Hz, 1H, C7aH), 8.78 (dd, ^3^*J* = 6.2 Hz, ^4^*J* = 1.6 Hz, 1H, C7bH), 8.70 (*td*, ^3^*J* = 8.1 Hz, ^4^*J* = 1.6 Hz, 1H, C5aH), 8.45 (*d*, ^3^*J* = 8.0 Hz, 1H, C4aH), 8.40–8.33 (*m*, 1H, C4bH), 8.31–8.26 (*m*, 1H, C5bH), 8.05 (*t*, ^3^*J* = 7.8 Hz, ^3^*J* = 5.8 Hz, 1H, C6aH), 7.84–7.77 (*m*, 1H, C6bH), 7.37 (*t*, ^3^*J* = 8.9 Hz, 1H, C10bH), 7.27 (*t*, ^3^*J* = 9.0 Hz, 1H, C10aH), 6.67–6.53 (*m*, 2H, C11H, C13H), 2.79, 2.77, 2.75 (*s*, 3H, C16aH), 2.52 (*s*, 3H, C16bH).

**33a·1/3CHCl**_**3**_: Yield: 52 mg (55%). mp 190 °C. C_19_H_18_ClN_4_O_5_ReS·1/3CHCl_3_ (671.4): Calcd C, 34.5; H, 2.7; N, 8.3; S, 4.7. Found: C, 34.2; H, 2.9; N, 8.4; S, 5.1%. MS-ESI [*m*/*z* (%)]: 601 (100) |M – Cl|^+^, 642 (85) |M – Cl + CH_3_CN|^+^. IR data (ATR, ν/cm^–1^): 3195b ν(NH, OH); 2018s, 1879vs ν(C=O_fac_); 1552m, 1510m ν(C=N); 1244m ν(C–OH); 1028s ν(O–CH_3_); 748m ν(C=S).

^1^H NMR (400 MHz, DMSO-*d*_6_, ppm): 11.88 (*s*, 1H, N2H), 10.31 (*s*, 1H, N1H), 8.54 (*d*, ^3^*J* = 4.9 Hz, 1H, C7H), 8.30 (*d*, ^3^*J* = 9 Hz, 1H, C5H), 8.20 (*d*, ^3^*J* = 9 Hz, 1H, C4H), 7.22 (*d*, ^3^*J* = 8.8 Hz, 2H, C10H, C14H), 6.87 (*d*, ^3^*J* = 8.8 Hz, 2H, C11H, C13H), 4.80–4.63 (*m*, 1H, C8aH), 4.60–4.18 (*m*, 1H, C8bH), 3.71 (*s*, 3H, C16H), 2.59 (*s*, 3H, C15H).

### Synthesis of [Re(L^13^)(CO)_3_] (**13c**)

A suspension of *fac*-[ReCl(CH_3_CN)_2_(CO)_3_] (61 mg; 0.16 mmol) and **HL**^**13**^ (48 mg; 0.16 mmol) in CHCl_3_ (5 mL) was heated under reflux for 2 h NEt_3_ (44 μL; 0.32 mmol) was added, and the mixture was heated under reflux for a further 1 h. The resulting solution was concentrated under vacuum to half of its initial volume and stored at 4 °C after adding diethyl ether. The resulting solid was filtered off, washed with water, and vacuum dried over CaCl_2_/KOH.

**13c**: Yield: 50 mg (55%). mp 198 °C. C_18_H_15_N_4_O_4_ReS (570.0): Calcd C, 37.9; H, 2.7; N, 9.8; S, 5.6. Found: C, 38.0; H, 2.8; N, 9.7; S, 5.4%. MS-ESI [*m*/*z* (%)]: 571 (100) |M + H|^+^. IR data (ATR, ν/cm^–1^): 3327b ν(NH, OH); 2008s, 1915s, 1896vs ν(C=O_fac_); 1535m, 1505s, 1461m ν(C=N); 1028s ν(O–CH_3_); 813s ν(C=S).

^1^H NMR (400 MHz, DMSO-*d*_6_, ppm): 8.69 (*dt*, ^3^*J* = 3.8 Hz, ^4^*J* = 0.9 Hz, 1H, C7H), 8.65 (*br*, 1H, C2H), 8.40 (*br*, 1H, C5H), 8.19 (*br*, 1H, C4H), 7.82–7.78 (*m*, 1H, N1H), 7.41 (*ddd*, ^3^*J* = 7.5 Hz, ^3^*J* = 4.7 Hz, ^4^*J* = 0.9 Hz, 1H, C6H), 7.27 (*d*, ^3^*J* = 8.6 Hz, 2H, C10H, C14H), 6.90 (*d*, ^3^*J* = 8.6 Hz, 2H, C11H, C13H), 4.54 (*dd*, ^2^*J* = 14.9 Hz, ^3^*J* = 6.0 Hz, 1H, C8aH), 4.47 (*br*, 1H, C8bH), 3.70 (*s*, 3H21 C16H).

### Synthesis of the Complexes [Re(L^21–24^)(CO)_3_] (**21c–24c**)

A suspension of equimolar amounts of *fac*-[ReX(CH_3_CN)_2_(CO)_3_] (X = Cl for **21c**, **23c**; X = Br for **22c**, **24c**) and **HL**^**21–24**^ in CHCl_3_ was heated under reflux for 1 h. NEt_3_ (0.1 mL; 0.72 mmol) was added, and the mixture was heated under reflux for about 2 h (see Supporting Information for details). The resulting solution was concentrated under vacuum to half of its initial volume and stored at 4 °C after adding diethyl ether. The resulting solid was filtered off, washed with water, and vacuum dried over CaCl_2_/KOH. Reagent amounts and synthesis conditions are collected in the Supporting Information.

**21c·2/5(C**_**4**_**H**_**10**_**O)**: Yield: 66 mg (63%). mp > 300 °C. C_17_H_13_N_4_O_4_ReS 2/5(C_4_H_10_O) (585.7): Calcd C, 38.1; H, 2.9; N, 9.6; S, 5.5. Found: C, 38.1; H, 2.9; N, 10.0; S, 5.5%. MS-ESI [*m*/*z* (%)]: 557 (100) |M + H|^+^. IR data (ATR, ν/cm^–1^): 3251b ν(NH, OH); 2009s, 1875vs ν(C=O_fac_); 1504m, 1464m ν(C=N); 826m ν(C=S).

^1^H NMR (400 MHz, DMSO-*d*_6_, ppm): 9.42 (*s*, 1H, O2H), 9.10 (*d*, ^3^*J* = 5.3 Hz, 1H, C7H), 9.07 (*s*, 1H, N1H), 8.28–8.27 (*m*, 2H, C5H, C4H), 7.65 (*q*, ^3^*J* = 4.8 Hz, 1H, C6H), 7.50 (*d*, ^3^*J* = 8.9 Hz, 2H, C10H, C14H), 6.69 (*d*, ^3^*J* = 8.9 Hz, 2H, C11H, C13H), 2.45 (*s*, 3H, C15H).

**22c·2/3(C**_**4**_**H**_**10**_**O)**: Yield: 50 mg (61%). mp > 300 °C. C_17_H_12_FN_4_O_4_ReS 2/3(C_4_H_10_O) (623.4): Calcd C, 37.9; H, 3.0; N, 9.0; S, 5.1. Found: C, 38.2; H, 2.6; N, 9.2; S, 5.0%. MS-ESI [*m*/*z* (%)]: 575 (100) |M + H|^+^, 616 (96) |M + H + CH_3_CN|^+^. IR data (ATR, ν/cm^–1^): 3213b ν(NH, OH); 2011s, 1885vs ν(C=O_fac_); 1509s, 1469m ν(C=N); 1150s ν(C–OH); 843m ν(C=S).

^1^H NMR (400 MHz, DMSO-*d*_6_, ppm): 9.72 (*s*, 1H, O2H), 9.09 (*d*, ^3^*J* = 5.2 Hz, 1H, C7H), 8.83 (*s*, 1H, N1H), 8.27 (*td*, ^3^*J* = 8.1 Hz, ^4^*J* = 1.2 Hz, 1H, C5H), 8.23 (*d*, ^3^*J* = 7.6 Hz, 1H, C4H), 7.63 (*ddd*, ^3^*J* = 7.2 Hz, ^3^*J* = 5.2 Hz, ^4^*J* = 2.0 Hz, 1H, C6H), 7.29 (*d*, ^3^*J* = 8.8 Hz, ^4^*J* = 9.5 Hz, 1H, C10H), 6.59 (*dd*, ^3^*J* = 10.4 Hz, ^4^*J* = 2.6 Hz, 1H, C13H), 6.56 (*dd*, ^3^*J* = 6.3 Hz, ^4^*J* = 2.6 Hz, 1H, C11H), 2.41 (*s*, 3H, C15H).

**23c·1/5(C**_**4**_**H**_**10**_**O)**: Yield: 40 mg (33%). mp 139 °C. C_19_H_17_N_4_O_4_ReS 1/5(C_4_H_10_O) (598.9): Calcd C, 39.7; H, 3.2; N, 9.4; S, 5.3. Found: C, 39.8; H, 3.1; N, 9.6; S, 5.1%. MS-ESI [*m*/*z* (%)]: 585 (100) |M + H|^+^, 626 (24) |M + H + CH_3_CN|^+^. IR data (ATR, ν/cm^–1^): 3324b ν(NH, OH); 2009s, 1877vs ν(C=O_fac_); 1509m, 1465m, ν(C=N); 1032s ν(O–CH_3_); 823s ν(C=S).

^1^H NMR (400 MHz, DMSO-*d*_6_, ppm): 9.04 (*d*, ^3^*J* = 5.2 Hz, 1H, C7H), 8.23 (*td*, ^3^*J* = 7.9 Hz, ^4^*J* = 1.2 Hz, 1H, C5H), 8.17 (*d*, ^3^*J* = 7.9 Hz, 1H, C4H), 7.89 (*t*, ^3^*J* = 6.0 Hz, 1H, N1H), 7.60 (*td*, ^3^*J* = 7.1 Hz, ^3^*J* = 5.8 Hz, 1H, C6H), 7.24 (*d*, ^3^*J* = 8.6 Hz, 2H, C10H, C14H), 6.86 (*d*, ^3^*J* = 8.6 Hz, 2H, C11H, C13H), 4.38 (*dd*, ^2^*J* = 15.1 Hz, ^3^*J* = 6.0 Hz, 1H, C8aH), 4.32 (*dd*, ^2^*J* = 15.1 Hz, ^3^*J* = 6.0 Hz, 1H, C8bH), 3.72 (*s*, 3H, C16H), 2.30 (*s*, 3H, C15H).

**24c·4/7(CHCl**_**3**_**)**: Yield: 30 mg (39%). mp 247 °C. C_18_H_15_N_4_O_4_ReS 4/7(CHCl_3_) (637.4): Calcd C, 35.0; H, 2.5; N, 8.8; S, 5.0. Found: C, 35.4; H, 2.2; N, 8.4; S, 5.3%. MS-ESI [*m*/*z* (%)]: 571 (100) |M + H|^+^, 612 (43) |M + H + CH_3_CN|^+^. IR data (ATR, ν/cm^–1^): 3241b ν(NH, OH); 2011s, 1879vs ν(C=O_fac_); 1513s, 1470m, ν(C=N); 825m ν(C=S).

^1^H NMR (400 MHz, DMSO-*d*_6_, ppm): 9.20 (*s*, 1H, O2H), 9.04 (*d*, ^3^*J* = 5.1 Hz, 1H, C7H), 8.23 (*td*, ^3^*J* = 7.7 Hz, ^4^*J* = 1.2 Hz, 1H, C5H), 8.18 (*d*, ^3^*J* = 7.9 Hz, 1H, C4H), 7.83 (*t*, ^3^*J* = 5.9 Hz, 1H, N1H), 7.60 (*ddd*, ^3^*J* = 7.2 Hz, ^3^*J* = 5.8 Hz, ^4^*J* = 1.6 Hz, 1H, C6H), 7.11 (*d*, ^3^*J* = 8.5 Hz, 2H, C10H, C14H), 6.68 (*d*, ^3^*J* = 8.5 Hz, 2H, C11H, C13H), 4.33 (*dd*, ^2^*J* = 15.1 Hz, ^3^*J* = 5.9 Hz, 1H, C8aH), 4.27 (*dd*, ^2^*J* = 15.1 Hz, ^3^*J* = 5.9 Hz, 1H, C8bH), 2.31 (*s*, 3H, C15H).

### Synthesis of [Re(L^33–34^(CO)_3_] (**33c** and **34c**)

A suspension of *fac*-[ReCl(CH_3_CN)_2_(CO)_3_] (60 mg; 0.15 mmol) and **HL**^**33**^ (50 mg; 0.15 mmol) or **HL**^**34**^ (50 mg; 0.16 mmol) in CHCl_3_ (10 mL) was heated under reflux for 2 h. Then, NEt_3_ (22 μL; 0.16 mmol) was added, and the mixture was heated for a further 4 h. The resulting solution was concentrated under vacuum to half of its initial volume and stored at 4 °C after adding diethyl ether or tetrahydrofuran. The resulting solid was filtered off, washed with water, and vacuum dried over CaCl_2_/KOH.

**33c·2/5(C**_**4**_**H**_**10**_**O)**: Yield: 38 mg (42%). mp 157 °C. C_19_H_17_N_4_O_5_ReS 2/5(C_4_H_10_O) (598.9): Calcd C, 39.3, H, 3.4; N, 8.9; S, 5.0. Found: C, 39.3; H, 3.6; N, 9.2; S, 4.5%. MS-ESI [*m*/*z* (%)]: 601 (100) |M + H|^+^. IR data (ATR, ν/cm^–1^): 2969b ν(NH, OH); 2004s, 1867vs ν(C=O_fac_); 1569m, 1509s, 1456m, ν(C=N); 1028s ν(O–CH_3_); 830s ν(C=S).

^1^H NMR (400 MHz, DMSO-*d*_6_, ppm): 8.41 (*d*, ^4^*J* = 2.5 Hz, 1H, C7H), 7.90 (*d*, ^3^*J* = 9.0 Hz, 1H, C4H), 7.62 (*t*, ^3^*J* = 6.0 Hz, 1H, N1H), 7.35 (*dd*, ^3^*J* = 9.0 Hz, ^4^*J* = 2.1 Hz, 1H, C5H), 7.22 (*d*, ^3^*J* = 8.5 Hz, 2H, C10H, C14H), 6.85 (*d*, ^3^*J* = 8.5 Hz, 2H, C11H, C13H), 4.33 (*dd*, ^2^*J* = 15.2 Hz, ^3^*J* = 6.0 Hz, 1H, C8aH), 4.28 (*dd*, ^2^*J* = 15.2 Hz, ^3^*J* = 6.0 Hz, 1H, C8bH), 3.71 (*s*, 3H, C16H), 2.15 (*s*, 3H, C15H).

**34c·1/3(C**_**4**_**H**_**8**_**O)**: Yield: 15 mg (17%). mp 198 °C. C_18_H_15_N_4_O_5_ReS·1/3(C_4_H_8_O) (610.1): Calcd C, 38.0; H, 2.9; N, 9.2; S, 5.2, Found: C, 37.7; H, 3.2; N, 9.2; S, 4.7%. MS-ESI [*m*/*z* (%)]: 587 (100) |M + H|^+^. IR data (ATR, ν/cm^–1^): 2979b ν(NH, OH); 2009s, 1874vs ν(C=O_fac_); 1569m, 1515s, 14673m, ν(C=N); 833m ν(C=S).

^1^H NMR (400 MHz, DMSO-*d*_6_, ppm): 9.23 (*br*, 2H, O1H, O2H), 8.27 (*d*, ^4^*J* = 2.6 Hz, 1H, C7H), 7.80 (*d*, ^3^*J* = 9.0 Hz, 1H, C4H), 7.46 (*t*, ^3^*J* = 6.1 Hz, 1H, N1H), 7.16 (*dt*, ^3^*J* = 9.0 Hz, ^4^*J* = 2.0 Hz, 1H, C5H), 7.09 (*d*, ^3^*J* = 8.5 Hz, 2H, C10H, C14H), 6.66 (*d*, ^3^*J* = 8.5 Hz, 2H, C11H, C13H), 4.27 (*dd*, ^2^*J* = 15.1 Hz, ^3^*J* = 6.1 Hz, 1H, C8aH), 4.23 (*dd*, ^2^*J* = 15.1 Hz, ^3^*J* = 6.1 Hz, 1H, C8bH), 2.13 (*s*, 3H, C15H).

### Synthesis of the Complexes [Re(HL^11-OEt^)(CO)_3_]Br (**11d**) and [Re(HL^13-OEt^)(CO)_3_]Br (**13d**)

A solution of (*fac*-[ReBr(CH_3_CN)_2_(CO)_3_]/[ReBr(CO)_5_]) and **HL**^**11,13**^ was heated under reflux for 6 h. The resulting solution was concentrated in vacuum to half of its initial volume and stored at 4 °C after adding diethyl ether. The resulting solid (crystalline phase of **11d**) was filtered off and vacuum dried over CaCl_2_/KOH. Reagent amounts and synthesis conditions are collected in the Supporting Information.

**11d**: Yield: 30 mg (46%). mp 222 °C. C_17_H_16_BrN_4_O_5_ReS (654.0): Calcd C, 31.2; H, 2.5; N, 8.6; S, 4.9. Found: C, 31.3; H, 2.2; N, 8.5; S, 5.0%. MS-ESI [*m*/*z* (%)]: 543 (80) |M – Br – OMe|^+^, 575 (100) |M – Br|^+^. IR data (ATR, ν/cm^–1^): 3231b ν(NH, OH); 2028s, 1916s, 1894vs ν(C=O_fac_); 1579w, 1535m, 1508m ν(C=N); 835m ν(C=S).

^1^H NMR (400 MHz, DMSO-*d*_6_, ppm): 11.39 (*br*, 1H, N2H), 10.27 (*br*, 1H, N1H), 9.77 (*br*, 1H, N3H), 8.73 (*d*, ^3^*J* = 4.7 Hz, 1H, C7H), 8.22 (*t*, ^3^*J* = 7.3 Hz, 1H, C5H), 8.10 (*br*, 1H, C4H), 7.67 (*t*, ^3^*J* = 6.0 Hz, 1H, C6H), 6.93 (*br*, 2H, C10H, C14H), 6.79 (*br*, 2H, C11H, C13H), 5.76 (*s*, 1H, C2H), 3.73 (*s*, 3H, CH_3_–O).

**13d (H**_**2**_**O)**: Yield: 10 mg (7%). *T*_dec_: 176 °C. C_20_H_22_BrN_4_O_5_ReS·(H_2_O) (714.0): Calcd C, 33.6; H, 3.4; N, 7.8; S 4.5. Found: C, 33.8; H, 3.0; N, 7.6; S; 4.6%. MS-ESI [*m*/*z* (%)]: 617 (100) |M – Br|^+^. IR data (ATR, ν/cm^–1^): 3199b ν(NH, OH); 2024s, 1906vs ν(C=O_fac_); 1613w, 1585m, 1513m ν(C=N); 1027s ν(O–CH_3_); 817m ν(C=S).

^1^H NMR (400 MHz, DMSO-*d*_6_, ppm): 11.55 (*br*, 1H, N2H), 10.86–10.29 (*m*, 1H, N1H), 9.73–8.97 (*m*, 1H, N3H), 8.72–8.66 (*m*, 1H, C7H), 8.21 (*br*, 1H, C5H), 8.06–7.92 (*m*, 1H, C4H), 7.65 (*br*, 1H, C6H), 6.99 (*d*, ^3^*J* = 7.5 Hz, 2H, C10H, C14H), 6.83 (*d*, ^3^*J* = 7.5 Hz, 2H, C11H, C13H), 5.88–5.82 (*m*, 1H, C2H), 4.40 (*br*, 2H, C8H), 4.15 (*q*, ^3^*J* = 7.0 Hz, 1H, CH_3_–CH_2_–O), 3.96 (*q*, ^3^*J* = 7.0 Hz, 1H, CH_3_–CH_2_–O), 3.73 (*s*, 3H, C16H), 1.39 (*t*, ^3^*J* = 7.0 Hz, 1H, CH_3_–CH_2_–O), 1.23 (*t*, ^3^*J* = 7.0 Hz, 2H, CH_3_–CH_2_–O).

### Synthesis of [Re(L^11-OMe^)(CO)_3_] (**11e**)

*fac*-[ReCl(CH_3_CN)_2_(CO)_3_] (89 mg, 0.15 mmol) and **HL**^**11**^ (42 mg, 0.15 mmol) were mixed in MeOH (10 mL), and the solution was heated under reflux for 2 h. A suspension of NaOH (9 mg, 0.23 mmol) in 3 mL of the same solvent was added, and the mixture was heated under reflux for 1 h. The resulting solution was concentrated to half of its initial volume and stored at 4 °C after adding chloroform. The resulting crystals were filtered off, washed with water, and vacuum dried over CaCl_2_/KOH.

**11e·3/4(CHCl**_**3**_**)**: Yield: 6 mg (7%). C_17_H_15_N_4_O_5_ReS·3/4 (CHCl_3_) (662.5): Calcd C, 32.2; H 2.4; N, 8.5; S, 4.8. Found: C, 32.3; H, 2.2; N, 8.4; S, 4.5%. MS-ESI [*m*/*z* (%)]: 543 (52) |M – OCH_3_|^+^, 575 (100) |M + H|^+^. IR data (ATR, ν/cm^–1^): 3321b ν (NH, OH); 2012s, 1868vs ν(C=O_fac_); 1507s ν (C=N); 767m ν (C=S).

^1^H NMR (400 MHz, DMSO-*d*_6_, ppm): 10.00 (*d*, *J* = 3.4 Hz, 1H, N3H), 8.80 (*s*, 1H, O2H), 8.53 (*d*, ^3^*J* = 5.1 Hz, 1H, C7H), 8.35 (*s*, 1H, N1H), 8.03 (*td*, ^3^*J* = 7.8 Hz, ^4^*J* = 1.5 Hz, 1H, C5H), 7.57 (*d*, ^3^*J* = 7.8 Hz, 1H, C4H), 7.53 (*t*, ^3^*J* = 6.5 Hz, 1H, C6H), 7.35 (*d*, ^3^*J* = 8.9 Hz, 2H, C10H, C14H), 6.53 (*d*, ^3^*J* = 8.9 Hz, 2H, C11H, C13H), 5.66 (*d*, ^3^*J* = 3.4 Hz, 1H, C2H), 3.83 (*s*, 3H, CH_3_–O).

### Formation of the Trinuclear Complex **[Re**_**3**_**Cl**_**2**_**(L**^**23**^**)(HL**^**23**^**)(CO)**_**9**_**]**

*fac*-[ReCl(CH_3_CN)_2_(CO)_3_] (47 mg, 0.12 mmol) and **HL**^**23**^ (52 mg; 0.17 mmol) were dissolved in CHCl_3_ (10 mL), and the solution was heated under reflux for 3 h. The resulting solution was concentrated to half of its initial volume and stored at 4 °C. The first solid formed was the chlorohydrate salt of the ligand. The second crop of crystals was filtered off and vacuum dried over CaCl_2_/KOH.

MS-ESI [*m*/*z* (%)]: 315 (100) |HL + H|^+^, 585 (90) |Re(CO)_3_(HL)|^+^, 899 (10) |Re(CO)_3_(HL)_2_|^+^, 1205 (4) |Re_2_(CO)_6_(HL)_2_Cl|^+^, 1437 (3) |M – Cl_2_|^+^. IR data (ATR, ν/cm^–1^): 3169b ν(NH, OH); 2011s, 1881vs ν(C=O_fac_); 1565m, 1509s, 1439m, ν(C=N); 1029s ν(O–CH_3_); 763s ν(C=S).

### Formation of the Dinuclear Complex [Re_2_(L^13^)_2_(CO)_6_ (**13f**)

In an NMR tube complex, **13b** was dissolved in MeOD and NEt_3_ was added (about 15 equiv). The signals of compound **13f** appeared, while the signals due to **13b** disappeared from the spectrum. Crystals formed in the tube and X-ray diffraction confirmed the dimeric structure.

IR data (ATR, ν/cm^–1^): 3308b ν(NH, OH); 2009s, 1925vs, 1892s ν(C=O_fac_); 1532s, 1512vs ν(C=N); 1031s ν(O–CH_3_); 769m ν(C=S).

^1^H NMR (400 MHz, CD_3_OD, ppm): 8.77 (*d*, ^3^*J* = 4.5 Hz, 1H, C7H), 8.54 (*d*, ^3^*J* = 8.0 Hz, 1H, C4H), 8.40 (*s*, 1H, C2H), 7.55 (*td*, ^3^*J* = 7.9 Hz, ^4^*J* = 1.8 Hz, 1H, C5H), 7.43 (*dd*, ^3^*J* = 8.0 Hz, ^3^*J* = 4.5 Hz, 1H, C6H), 7.20 (*d*, ^3^*J* = 8.7 Hz, 2H, C10H, C14H), 6.84 (*d*, ^3^*J* = 8.7 Hz, 2H, C11H, C13H), 4.20 (*q*, ^2^*J* = 14.5 Hz, 2H, C8H), 3.75 (*s*, 3H, C16H).

## Supporting Information Available

Synthesis of the ligands and complexes, NMR and IR spectra, and single-crystal X-ray diffraction studies (PDF)
