# Supplementary material for: Coordination Chemistry of Potentially S,N,Npy-Tridentate Thiosemicarbazones with the {Re(CO)3}+ Fragment and Formation of Hemiaminal Derivatives
Source: Inorg Chem. 2022 Dec 22;62(1):224–37. doi: 10.1021/acs.inorgchem.2c03259 (PMC9832478; doi:10.1021/acs.inorgchem.2c03259)
Supplement: Supplementary file 1 — ic2c03259_si_001.pdf [file ic2c03259_si_001.pdf]

The coordination chemistry of potentially S,N,N_{py}-tridentate thiosemicarbazones with the $\{Re(CO)_3\}^+$ fragment and formation of hemiaminal derivatives

Supporting Information

Saray Argibay-Otero, Rosa Carballo, Ezequiel M. Vázquez-López*

***E-mail: ezequiel@uvigo.es**

Departamento de Química Inorgánica, Facultade de Química, Instituto de Investigación Sanitaria Galicia Sur, Universidade de Vigo, Campus Universitario, E-36310 Vigo, Galicia, Spain;

Metallosupramolecular Chemistry Group, Galicia South Health Research Institute (IIS Galicia Sur), SERGAS-UVIGO, E-36213 Vigo, Galicia, Spain;

Table S1. Crystal and structure refinement 3
Crystal and molecular structures of the free ligands 7
Figure S1. Representations of the molecular structures of the thiosemicarbazone ligands and the hydrochlorides 8
Table S2. Selected bond lengths (Å) and angles (°) for the ligands 10
Table S3. Hydrogen bonds and secondary interactions 11
Figure S2. The structure of the formamidrazonium cation 13
Figure S3. The asymmetric unit in the structures of the acetone (a) and chloroform (b) solvates of $[ReCl(HL^{13})(CO)_3]$ (13a) emphasizing the different association by H-bonding 14
Figure S4. Molecular structures of $[Re(HL^{13OEt})(CO)_3]Br.½H_2O$ 15
(where only one of the two molecules of the asymmetric unit is depicted 15
Synthesis of pyridine thiosemicarbazone ligands 16
Table S3. Details of synthetic conditions used for the synthesis of the ligands 16
HL^{11} 17
HL^{12} 18
HL^{13} 19
HL^{21} 22
HL^{22} 23
HL^{23} 24
HL^{24} 25
HL^{33} 26
HL^{34} 27
Synthesis of $[ReX(HL^n)(CO)_3]$ (X=Cl: 13a, 22a, 33a; X=Br: 13b) 28
Table S4. Details of synthetic conditions used for the synthesis of 13a, 22a, 33a and 13b ..28
13a: 29
$13b·4/5(CH_3CH_2OH)$: 30

$22a\cdot 1/2CHCl_3$ 31
$33a\cdot 1/3CHCl_3$ 32
Synthesis of [Re(L^{13})($CO)_3$] (13c) 33
Synthesis of the complexes [Re(L^n)($CO)_3$] (21c-24c) 34
Table S5. Details of synthetic conditions used for the synthesis of 21c-24c 34
$21c\cdot 2/5(C_4H_{10}O)$ 35
$22c\cdot 2/3(C_4H_{10}O)$ 36
$23c\cdot 1/5(C_4H_{10}O)$ 37
$24c\cdot 4/7(CHCl_3)$ 38
Synthesis of [Re(L^n)($CO)_3$] (33c and 34c) 39
$33c\cdot 2/5(C_4H_{10}O)$ 39
$34c\cdot 1/3(C_4H_8O)$ 40
Synthesis of the complexes [Re($HL^{11\text{-}OMe}$)($CO)_3$]Br (11d) and [Re($HL^{13\text{-}OEt}$)($CO)_3$]Br (13d) 41
Table S6. Details of synthetic conditions used for the synthesis of 11d and 13d 41
11d: 42
13d $4/5(H_2O)$: 43
Synthesis of [Re($L^{11\text{-}OMe}$)($CO)_3$] (11e) 44
$11e\cdot 3/4(CHCl_3)$: 44
Formation of the trinuclear complex [Re_3Cl_2(L)(HL)($CO)_9$] 45
[Re_3Cl_2(L^{23})(HL)($CO)_9$].3($CHCl_3$) 45
Formation of the dinuclear complex [Re_2(L^{13})$_2$($CO)_6$ (13f) 46
References: 48

Table S1. Crystal and structure refinement

Compound	$HL^{11}.(C_3H_6O)$	$HL^{12}.(H_2O)$	HL^{13}(E)	HL^{13}(Z)	$[H_2L^{13}]Cl$
Deposition code:	2206748	2206749	2206750	2206751	2206752
Empirical formula	$C_{16}H_{18}N_4O_2S$	$C_{13}H_{15}FN_4O_3S$	$C_{15}H_{16}N_4OS$	$C_{15}H_{16}N_4OS$	$C_{15}H_{17}ClN_4OS$
Formula weight	330.40	326.35	300.38	300.38	336.83
Temperature (K)	115(2)	100(2)	100(2)	100(2)	100(2)
λ(Å)	0.71073	1.54178	0.71073	0.71073	0.71073
Crystal system	Monoclinic	Triclinic	Monoclinic	Monoclinic	Triclinic
Space group	*Cc*	*P -1*	*P 2₁/n*	*P 2₁/c*	*P -1*
Unit cell dimensions					
a(Å)	11.1042(5)	6.6915(6)	9.5825(6)	18.4554(16)	7.4472(5)
b(Å)	25.7156(11)	9.7518(11)	5.5064(3)	5.8851(5)	8.0376(4)
c(Å)	11.7163(5)	13.9932(11)	28.8548(18)	13.5523(11)	14.0423(9)
α(°)	90	102.616(7)	90		86.913(2)
βα(°)	102.483(2)	102.809(6)	98.710(2)	101.529(3)	89.449(2)
γ(°)	90	105.029(7)	90		66.983(2)
Volume($Å^3$)	3266.5(2)	822.20(14)	1504.97(16)	1442.2(2)	772.44(8)
Z	8	2	4	4	2
ρ_c(Mg/m^3)	1.344	1.318	1.326	1.383	1.448
μ(mm^{-1})	0.213	2.007	0.219	0.229	0.389
θ(°) range	2.38-28.34	3.38-65.36	2.79-28.34	2.25-28.31	2.75-28.30
(hkl) ranges	-14,14; -34,4; -15,15	-7,7; -11,11, -16,	-12,12; -7,7, -29,38	-24,24; -7,7; -16,18	-9,9; -10,10; -18,18
Reflections collected	28552	19817	24075	22042	33963
Independent reflections (R_{int})	8060(0.0465)	2770(0.1388)	3757(0.0356)	3546(0.0360)	3832(0.0338)
Data / restraints / parameters	8060 / 2 / 439	2770 / 7 / 233	3757 / 1 / 267	3546 / 0 / 198	3832/0/211
S on F^2	1.021	1.036	1.053	1.054	1.113
R1/wR1 [I>2sigma(I)]	0.0439/0.1030	0.0634/0.1623	0.0388/0.0843	0.0342/0.0843	0.0339/0.0765
R1/wR2 (all data)	0.0573/0.1082	0.0999/0.1914	0.0525/0.0900	0.0377/0.0861	0.0376/0.0786

Table S1 (cont.)

Compound	**HL^{22}**	**HL^{23}**	**$[H_2L^{23}]Cl.H_2O$**	**HL^{24}**	**$HL^{34}.H_2O.CHCl_3$**
Deposition code:	2206753	2206754	2206755	2206756	2206757
Empirical formula	$C_{14}H_{13}FN_4OS$	$C_{16}H_{18}N_4OS$	$C_{16}H_{21}ClN_4O_2S$	$C_{15}H_{16}N_4OS$	$C_{16}H_{19}Cl_3N_4O_3S$
Formula weight	304.34	314.40	368.88	300.38	453.76
Temperature (K)	100(2)	100(2)	100(2) K	100(2) K	100(2)
λ(Å)	0.71073	0.71073	0.71073 Å	0.71073 Å	0.71073
Crystal system	Monoclinic	Monoclinic	Triclinic	Triclinic	Triclinic
Space group	*P* 2_1/*n*	*P* 2_1/*c*	*P -1*	*P -1*	*P -1*
Unit cell dimensions					
a(Å)	12.4104(6)	13.3725(12)	6.7421(12)	5.9036(4)	5.6729(8)
b(Å)	19.8215(9)	13.1769(11)	8.5071(14)	11.5434(9)	12.2269(18)
c(Å)	12.5086(6)	9.3672(9)	15.741(3)	23.4289(17)	15.422(2)
α(°)		90	93.973(7)	102.334	69.745(4)
βα(°)	114.0580(10)	106.683(3)	101.168(6)	93.847(2)	87.631(5)
γ(°)		90	101.028(7)	102.507(2)	78.750(5)
Volume($Å^3$)	2809.7(2)	1581.1(2)	864.1(3)	1511.96(19)	983.9(2)
Z	8	4	2	4	2
ρ_c(Mg/m^3)	1.439	1.321	1.418	1.320	1.532
μ(mm^{-1})	0.246	0.212	0.359	0.218	0.597
θ(°) range	2.20-26.40	2.21-28.34	2.45-28.44	3.56-28.35	2.64-28.48
(hkl) ranges	-15,15; -24,24; -15,15	-17,16; -17,17; -12,12	-9,8; -10,10; 0,20	-7,7; -15,15; -31,31	-7,7; -16,16; -2020
Reflections collected	43838	32415	3982	51071	44842
Independent reflections (R_{int})	5752(0.0692)	3951(0.0508)	3982	7499(0.0405)	4922(0.0639)
Data / restraints / parameters	5752 / 6 / 381	3951 / 0 / 209	3982 / 1 / 229	7499 / 6 / 384	4922 / 0 / 254
S on F^2	1.055	1.065	1.095	1.030	1.103
R1/wR1 [I>2sigma(I)]	0.0486/0.1069	0.0391/0.0819	0.0876/0.1172	0.0400/ 0.0869	0.0639/0.1568
R1/wR2 (all data)	0.0741/0.1169	0.0538/0.0877	0.1570/0.1345	0.0544/0.0923	0.0818/0.1658

Table S1 (cont.)

Compound	**FMH**	**$[ReCl(HL^{13})(CO)_3]. (C_3H_6O)$**	**$[ReCl(HL^{13})(CO)_3]. ½(CHCl_3)$**	**$[Re_2(L^{13})_2(CO)_6]$**	**$[Re_3(L^{23})(HL^{23})(CO)_9].3(CHCl_3)$**
Deposition code:	2206765	2206758	2206759	2206760	2206764
Empirical formula	$C_{32}H_{48}N_8O_{17}S_3$	$C_{21}H_{22}ClN_4O_5ReS$	$C_{18.5}H_{16.5}Cl_{2.5}N_4O_4ReS$	$C_{36}H_{30}N_8O_8Re_2S_2$	$C_{44}H_{38}Cl_{11}N_8O_{11}Re_3S_2$
Formula weight	912.96	664.13	665.74	1139.20	1867.49
Temperature (K)	100(2)	100(2)	100(2)	100(2)	100(2)
λ(Å)	0.71073	0.71073	0.71073	0.71073	0.71073
Crystal system	Triclinic	Monoclinic	Monoclinic	Orthorhombic	Triclinic
Space group	*P -1*	*P 2_1/c*	*P 2_1/n*	*P b c n*	*P -1*
Unit cell dimensions					
a(Å)	8.7331(10)	24.7694(14)	11.5108(8)	19.5759(9)	12.0121(9)
b(Å)	9.9951(13)	14.9950(7)	24.1105(14)	10.7219(5)	12.0727(10)
c(Å)	24.071(3)	13.2486(7)	16.8045(11)	17.9949(8)	22.8367(19)
α(°)	81.391(4)	90	90	90	102.836(3)
βα(°)	87.636(4)	103.793(3)	98.874(2)°.	90	101.206(3)
γ(°)	75.698(4)	90	90	90	95.388(3)
Volume($Å^3$)	2013.0(4)	4778.9(4)	4607.9(5)	3777.0(3)	3134.7(4)
Z	2	8	8	4	2
ρ_c(Mg/m^3)	1.506	1.846	1.919	2.003	1.979
μ(mm^{-1})	0.269	5.324	5.687	6.578	6.372
θ(°) range	2.17-26.45	2.38-28.39	2.32-28.35	2.26-28.34	2.22-26.37
(hkl) ranges	-10,10;-12,12;-30,29	-33,33; -20,19; -17,17	-15,15; -32,32; -22,22	-26,26; -14,14; -23,23	-15,15; -15,15; -28,28
Reflections collected	71091	94194	90580	74011	106352
Independent reflections (R_{int})	8273(0.0752)	11917(0.1011)	11490(0.1102)	4699(0.0515)	12800(0.0505)
Data / restraints / parameters	8273 / 14 / 557	11917 / 4 / 595	11490 / 0 / 579	4699 / 0 / 257	12800 / 8 / 714
S on F^2	1.021	1.045	1.041	1.255	0.987
R1/wR1 [I>2sigma(I)]	0.0479/ 0.1114	0.0429/ 0.0714	0.0458/0.0674	0.0280/0.0682	0.0464/0.1112
R1/wR2 (all data)	0.0718/ 0.1216	0.0708/ 0.0777	0.0805/0.0751	0.0320/0.0698	0.0612/0.1208

Table S1 (cont.)

Compound	**[Re(HL^{11OEt})($CO)_3$]Cl. (EtOH)**	**[Re(HL^{13OEt})($CO)_3$]Br. ½(H_2O)**	**[Re(L^{13OMe})($CO)_3$]**
Deposition code:	2206761	2206762	2206763
Empirical formula	$C_{20}H_{24}ClN_4O_6ReS$	$C_{20}H_{23}BrN_4O_{5.50}ReS$	$C_{17}H_{15}N_4O_5ReS$
Formula weight	670.14	705.59	573,58
Temperature (K)	100(2)	100(2)	100(2)
λ(Å)	0.71073	0.71073	0.71073
Crystal system	Triclinic	Triclinic	Triclinic
Space group	*P -1*	*P -1*	*P -1*
Unit cell dimensions			
a(Å)	9.1076(4)	10.6734(5)	9.4162(7)
b(Å)	9.7146(5)	15.2315(8)	11.0179(8)
c(Å)	14.4230(8)	15.4506(8)	15.7159(11)
α(°)	79.197(2)	88.037(2)	76.663(3)
βα(°)	73.068(2)	84.146(2)	77.201(3)
γ(°)	80.324(2)	78.618(2)	69.933(3)
Volume(Å^3)	1190.37(11)	2449.3(2)	1471.99(19)
Z	2	4	2
ρ_c(Mg/m^3)	1.870	1.913	1.294
μ(mm^{-1})	5.348	6.717	4.223
θ(°) range	2.42-28.33	2.25-26.47	2.33-28.32
(hkl) ranges	-12,12; -12,12; -19,19	-13,13; -19,19; -19,19	-12,12; -14,14; -20,20
Reflections collected	41194	81947	62357
Independent reflections (R_{int})	5924(0.0337)	10090(0.1173)	7315(0.0635)
Data / restraints / parameters	5924 / 0 / 320	10090 / 6 / 642	7315 / 1 / 266
S on F^2	1.145	1.038	1.069
R1/wR1 [I>2sigma(I)]	0.0186/0.0368	0.0466/0.0683	0.0390/0.0971
R1/wR2 (all data)	0.0210/0.0373	0.0805/0.0755	0.0473/0.0998

Crystal and molecular structures of the free ligands

The X-ray structures of the ligands **HL^{11}**, **HL^{12}**, **HL^{22}**, **HL^{23}** and of the two isomers of **HL^{13}** and the hydrochlorides of **HL^{13}** and **HL^{23}** were determined and the molecular structures are shown in Figure S1. A selection of the main distances and angles are listed in Table S2.

The system related to **HL^{13}** will be discussed in greater detail due to the availability of more structural information, but it must be taken into account that, in principle, the structures of the rest of the ligands follow patterns common to all of them. The X-ray structure of **HL^{34}** will be discussed separately (*vide infra*) since the crystal contains the zwitterionic form.

In the first instance, and setting aside aside **HL^{13}** derivatives, all of the compounds including those that crystallize with different solvent molecules (such as **HL^{12}**.$2H_2O$ and **HL^{11}**.(C_3H_6O)) show a predominance in the thiosemicarbazone chain of the *E,E,E,Z* conformation, which appears to be independent of the nature of the substituents on the N1 and C2 atoms.

More variation seems to exist in the relative position of the pyridine nitrogen (N4), which is normally dependent on the presence or absence of hydrogen bond donor groups. Typically, when groups with hydrogen bond donor/acceptor capacity are present in the crystal, either water molecules, as in **HL^{12}**.$2H_2O$, or the phenyl OH group of another neighboring thiosemicarbazone molecule, as in **HL^{11}**, lead the pyridine ring to orient its N4 group in a converging direction for the formation of three-center hydrogen bonds (Figures S1a and S1b). Even in the absence of this interaction, the pyridine ring is practically coplanar with the thiosemicarbazone arm. The bond distances and angles in the thiosemicarbazide fragment are, as one would expect, consistent with some delocalization of the multiple bond along the chain.[1,2] However, the C1–S1, C2–N3, N2–N3 and N2–C1 distances suggest that, in spite of the delocalization, the canonical form depicted in Scheme 1 (see manuscript) is predominant.

The isolation of crystals of the two configurational isomers of HL^{13} and the hydrochloride allows a comparative study of the effect of the relative distribution of the different groups on the most widely used structural parameters in the study of metalated compounds.

In the crystal obtained from chloroform, **HL^{13}(Z)** has the *Z* conformation for the formal double bond C2=N3. This conformation is probably favored by the presence of an intramolecular hydrogen bond (*S(6)*) involving the hydrazine group N2–H and the pyridine nitrogen N4 (Figure S1d). These two groups are involved in the intramolecular bond and the molecules can only associate through the interaction of the thioamidic N1–H group and sulfur (Table S3). It is interesting to note that, despite this, the molecules manage to pack more efficiently than in the **HL^{13}(E)** isomer obtained from solutions in MeOH (as suggested by the higher density of the crystal in the *Z* isomer, 1.383 Mg/m^3, compared to that of the *E* isomer, 1.326 Mg/m^3, Table S1). The existence of disorder in **HL^{13}(E)** in the ring attached to the

N1 nitrogen is also consistent with this observation. In the latter structure, the pyridine nitrogen is oriented in a divergent/parallel disposition with respect to the thiosemicarbazone chain (Figure S1c) and the molecules associate in the crystal through N2–H…N4 interactions.

Figure S1. Representations of the molecular structures of the thiosemicarbazone ligands and the hydrochlorides:
(a) $HL^{11}.(C_3H_6O)$, (b) $HL^{12}.2H_2O$, (c) $HL^{13}(E)$, (d) $HL^{13}(Z)$, (e) $[H_2L^{13}]Cl$, (f) HL^{22}, (g) HL^{23}, (h) $[H_2L^{23}]Cl$, (i) HL^{24}, (j) $HL^{34}.CHCl_3.H_2O$

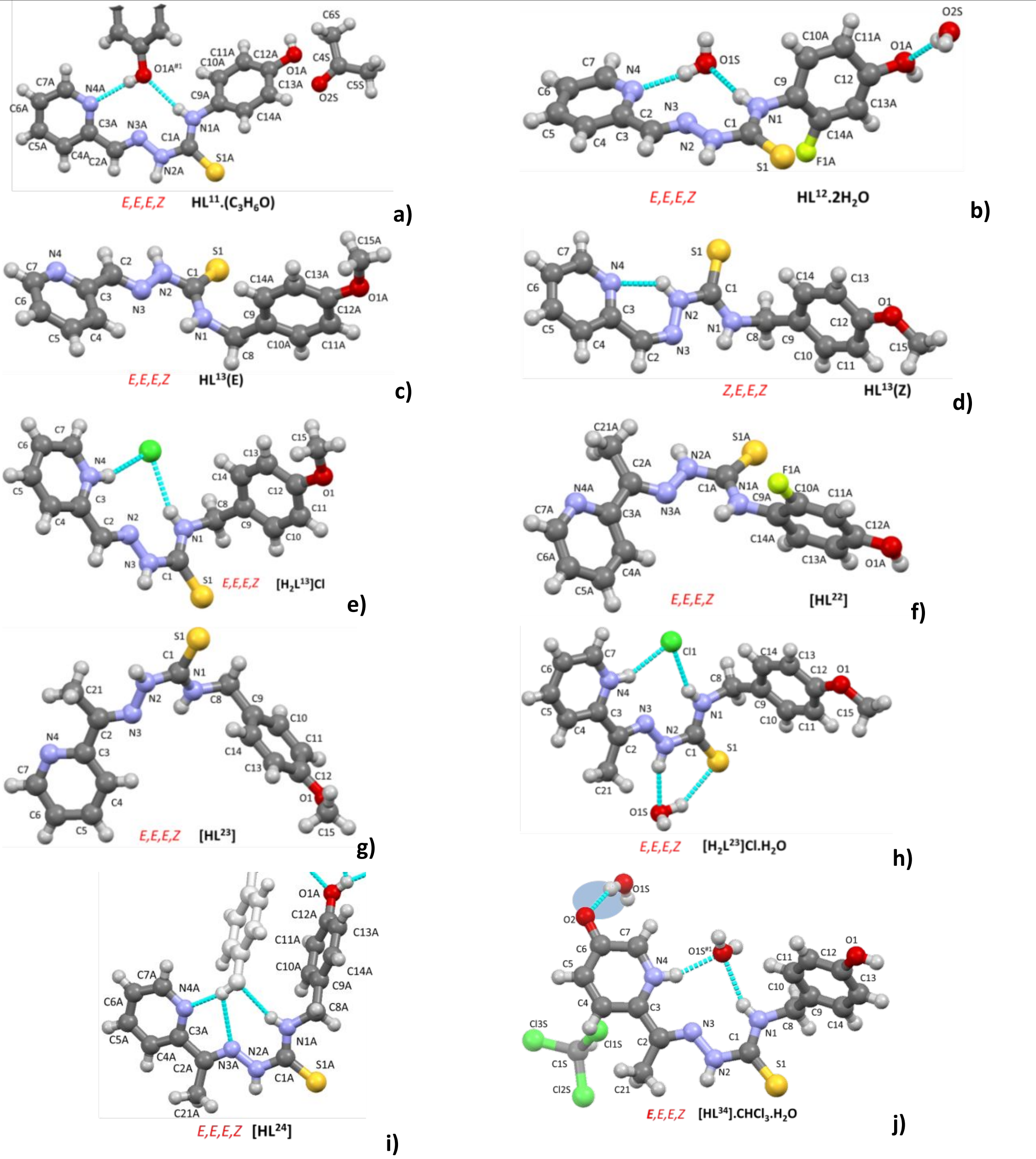

In the hydrochloride **[H$_2$L^{13}]Cl** the observed conformation is again *EEEZ* (Figure S1e), with the nitrogen N4 being protonated and the chloride anion associating with the thiosemicarbazinium cation via a double 'forked' hydrogen bond N4–H...Cl and N1–H...Cl. The molecular structure is very reminiscent of that already described for the cases of the hydrate **HL^{12}** and acetone solvate **HL^{11}**. It is important to note that although the values of the standard deviations do not allow differences to be discerned in the values of the bond distances in the TSC arm between the *E* and *Z* conformers, there are significant differences between the bonding distances in **[H$_2$L^{13}]$^+$** and **HL^{13}(E)**. For example, the N2–N3 distance is significantly shorter in the hydrochloride *versus* the two unprotonated isomers. Furthermore, S1–C1 and C2–N3 are longer and N1–C1 and N2–C2 are shorter (Table S2). It should be mentioned that although the standard deviation values in **[H$_2$L^{23}]Cl** are relatively high, comparison of the results with those for **HL^{23}** also suggests similar behavior in this hydrochloride (Figure S1h).

The structure of the ligand **HL^{34}** warrants separate consideration as it is based on the zwitterionic tautomer in which the phenolic hydrogen of the hydroxypyridine group is transferred to N4 (see Scheme, Figure S1j).

Once again, a water molecule establishes two interactions as an acceptor with the N4–H and N1–H groups in a distribution commonly called chelating (Figure S1j), an arrangement that surely plays a role in the stabilization of the zwitterion. On the other hand, the values of some of the bond distances (including the shortening of the phenoxide distance C6–O2, 1. 294(3) Å) suggest that the proton transfer also affects the thiosemicarbazone chain. For example, while the C1–S1 distance presents the expected value, the N1–C1 distance is one of the longest observed and the C1–N2 distance is much shorter than that observed in the rest of the structures of free ligands and their hydrochlorides.

Scheme. Solvent mediated proton transfer in HL^{34}.

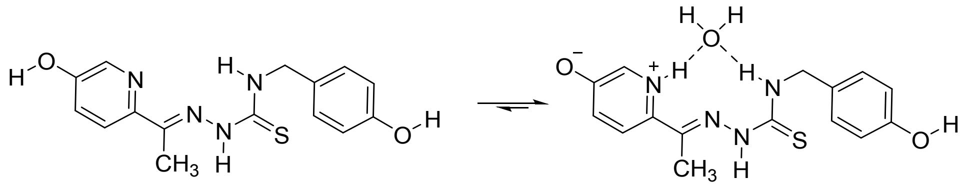

Table S2. Selected bond lengths (Å) and angles (°) for the ligands

	$HL^{11}.(C_3H_6O)^a$	$HL^{12}.(H_2O)$	HL^{13} (E)	HL^{13} (Z)	$[H_2L^{13}]Cl$
S1-C1	1.686(2)	1.665(4)	1.6759(13)	1.6831(12)	1.6854(14)
N1-C1	1.341(2)	1.337(5)	1.3390(17)	1.3309(15)	1.3259(19)
C1-N2	1.359(2)	1.362(4)	1.3541(18)	1.3704(14)	1.3779(18)
N2-N3	1.369(2)	1.363(4)	1.3683(15)	1.3676(14)	1.3491(16)
C2-N3	1.284(2)	1.279(4)	1.2764(17)	1.2931(15)	1.2846(19)
N1-C1-N2	116.0(2)	115.9(4)	115.87(12)	115.91(10)	116.09(12)
N1-C1-S1	125.9(1)	124.8(3)	124.62(11)	125.39(9)	126.14(11)
N2-C1-S1	118.1(1)	119.3(3)	119.50(10)	118.70(9)	117.77(11)
C1-N2-N3	120.4(2)	119.8(3)	119.61(11)	118.78(10)	118.82(12)
N3-C2-C3	120.5(2)	120.2(3)	120.74(11)	130.54(11)	117.13(13)
C2-N3-N2	115.7(2)	117.3(3)	115.38(11)	118.31(10)	118.27(12)

	$HL^{22\,a}$	HL^{23}	$[H_2L^{23}]Cl.H_2O$	HL^{24}	HL^{34}
S1-C1	1.679(1)	1.6925(14)	1.682(5)	1.6871(11)	1.687(3)
N1-C1	1.336(2)	1.3280(18)	1.323(6)	1.3272(13)	1.376(3)
C1-N2	1.362(2)	1.3572(18)	1.383(6)	1.3655(13)	1.325(4)
N2-N3	1.375(2)	1.3834(16)	1.361(5)	1.3669(12)	1.367(3)
C2-N3	1.289(2)	1.2866(18)	1.283(6)	1.2851(13)	1.293(3)
N1-C1-N2	115.2(1)	116.72(13)	116.4(4)	116.74(9)	116.3(2)
N1-C1-S1	123.95(12)	124.45(11)	125.2(4)	124.73(8)	125.0(2)
N2-C1-S1	120.67(13)	118.82(10)	118.4(4)	118.51(7)	118.7(2)
C1-N2-N3	117.1(1)	118.69(11)	120.0(4)	119.13(8)	118.7(2)
N3-C2-C3	113.7(1)	115.89(12)	114.5(4)	115.48(9)	114.3(2)
C2-N3-N2	119.8(1)	116.80(12)	116.7(4)	118.61(8)	118.9(2)

[a] Average values of the two molecules present in the asymmetric unit

Table S3. Hydrogen bonds and secondary interactions

**HL11.(C_3H_6O)**				
D-H...A	d(D-H)	d(H...A)	d(D...A)	<(DHA)
O(1A)-H(1A)...N(4A)#1	0.73(5)	2.12(5)	2.854(4)	175(4)
C(2A)-H(2AA)...S(1B)#2	0.95	2.88	3.680(3)	142.4
N(2A)-H(2A)...S(1B)#2	0.98(6)	2.37(6)	3.346(3)	170(4)
N(1A)-H(1AA)...O(1A)#3	0.80(4)	2.28(4)	3.050(3)	162(4)
N(1A)-H(1AA)...N(3A)	0.80(4)	2.28(4)	2.659(4)	110(3)
N(1B)-H(1BA)...O(1B)#1	0.88	2.12	2.975(3)	162.2
O(1B)-H(1B)...N(4B)#3	0.82(5)	2.01(5)	2.820(4)	170(5)
N(2B)-H(2B)...S(1A)#5	0.78(3)	2.60(4)	3.361(3)	165(3)
Symmetry transformations: #1 x,-y+1,z-1/2 #2 x+1/2,-y+1/2,z-1/2 #3 x,-y+1,z+1/2 #4 x-1/2,-y+1/2,z+1/2				
**HL12.(H_2O)**				
D-H...A	d(D-H)	d(H...A)	d(D...A)	<(DHA)
O(1)-H(1)...O(1S)#1	0.85	1.79	2.610(4)	161.4
N(2)-H(2)...S(1)#2	0.84	2.55	3.369(3)	166.5
N(1)-H(1A)...O(1S)	0.83	2.18	2.994(5)	165.3
O(1S)-H(1SA)...N(4)	0.83	2.00	2.827(4)	173.2
O(1S)-H(1SA)...N(3)	0.83	2.66	3.080(4)	112.6
O(1S)-H(1SB)...O(2S)#3	0.85	1.90	2.750(4)	174.5
O(2S)-H(2SB)...O(1)	0.84	2.01	2.842(4)	170.5
Symmetry transformations: #1 -x+1,-y+1,-z+1 #2 -x,-y,-z #3 -x,-y+1,-z+1				
**HL13 (E)**				
D-H...A	d(D-H)	d(H...A)	d(D...A)	<(DHA)
N(1)-H(1)...N(3)	0.845(19)	2.203(19)	2.6153(17)	110.0(15)
N(2)-H(2)...N(4)#1	0.885(16)	2.143(17)	3.0168(16)	168.7(14)
Symmetry transformations: #1 -x+1/2,y+1/2,-z+1/2				
**HL13 (Z)**				
D-H...A	d(D-H)	d(H...A)	d(D...A)	<(DHA)
N(1)-H(3)...S(1)#1	0.844(17)	2.836(16)	3.4043(11)	126.3(13)
N(1)-H(3)...N(3)	0.844(17)	2.196(16)	2.6107(14)	110.2(13)
N(2)-H(2N)...N(4)	0.862(17)	2.045(16)	2.7062(14)	132.8(14)
Symmetry transformations: #1 x,y+1,z				
**[H2L13]Cl**				
D-H...A	d(D-H)	d(H...A)	d(D...A)	<(DHA)
N(2)-H(2A)...Cl(1)#1	0.88(2)	2.46(2)	3.2955(13)	160.4(17)
N(1)-H(1)...Cl(1)	0.83(2)	2.48(2)	3.2500(13)	155.7(17)
N(4)-H(4B)...Cl(1)	0.85(2)	2.25(2)	3.0523(13)	156.8(19)
**HL22**				
D-H...A	d(D-H)	d(H...A)	d(D...A)	<(DHA)
O(1A)-H(1A)...N(4A)#1	0.85	1.92	2.755(3)	167.2
O(1B)-H(1B)...N(4B)#2	0.85	1.90	2.738(3)	169.7
N(2B)-H(2B)...S(1A)#3	0.85	2.56	3.396(2)	168.4
N(2A)-H(2A)...S(1B)#4	0.84	2.65	3.490(2)	174.6
N(1A)-H(1AA)...O(1B)#3	0.84	2.50	3.285(3)	155.1
Symmetry transformations: #1 x,y,z+1 #2 x,y,z-1 #3 x+1/2,-y+1/2,z+1/2 #4 x-1/2,-y+1/2,z-1/2				
**HL23**				
D-H...A	d(D-H)	d(H...A)	d(D...A)	<(DHA)
N(1)-H(1)...S(1)#1	0.832(17)	2.903(17)	3.4936(13)	129.8(13)
N(2)-H(2)...S(1)#2	0.883(19)	2.573(19)	3.4388(13)	166.6(15)
Symmetry transformations: #1 x,-y+1/2,z-1/2; #2 -x+1,-y+1,-z+2				

Table S3 (cont.)

**[H_2L^{23}]Cl.H_2O**				
D-H...A	d(D-H)	d(H...A)	d(D...A)	<(DHA)
N(2)-H(2)...O(1S)	0.79(5)	2.12(5)	2.892(6)	166(5)
O(1S)-H(1SA)...Cl(1)#1	0.87(2)	2.29(2)	3.135(5)	163(5)
O(1S)-H(1SB)...S(1)	0.93	2.34	3.188(4)	150.0
N(4)-H(4)...Cl(1)	0.88	2.25	3.057(4)	151.8
N(1)-H(1)...Cl(1)	0.88	2.35	3.163(4)	153.6
Symmetry transformations: #1 x,y+1,z				
**HL^{24}**				
D-H...A	d(D-H)	d(H...A)	d(D...A)	<(DHA)
O(1A)-H(1A)...N(3A)#1	0.85	2.65	3.1779(16)	121.9
O(1A)-H(1A)...N(4A)#1	0.85	1.91	2.7385(16)	164.7
N(1B)-H(1BA)...O(1B)#2	0.85	2.18	2.9983(16)	162.7
N(2B)-H(2B)...S(1A)#3	0.84	2.75	3.5914(12)	175.6
N(2A)-H(2A)...S(1B)#4	0.85	2.66	3.5054(12)	174.4
N(1A)-H(1AA)...O(1A)#1	0.82	2.25	3.0345(17)	161.2
O(1B)-H(1B)...N(3B)#2	0.836(16)	2.65(2)	3.1529(16)	120.4(19)
O(1B)-H(1B)...N(4B)#2	0.836(16)	1.917(17)	2.7358(18)	166(2)
Symmetry transformations: #1 x+1,-y+1,-z+1; #2 -x,-y+1,-z+2; #3 x+1,y,z; #4 x-1,y,z				
**HL^{34}**				
D-H...A	d(D-H)	d(H...A)	d(D...A)	<(DHA)
O(1)-H(1)...O(2)#1	0.82	1.76	2.577(3)	171.0
N(1)-H(1)...O(1S)#2	0.83	2.23	3.004(3)	155.3
N(4)-H(4)...O(1S)#2	0.84	2.02	2.817(3)	158.2
N(2)-H(2A)...S(1)#3	0.84	2.93	3.751(2)	166.8
Symmetry transformations: #1 x+1,y+1,z; #2 -x+1,-y+1,-z+1; #3 -x+2,-y+1,-z+2				
**[Re(HL^{13})$(CO)_3$].(C_3H_6O)**				
D-H...A	d(D-H)	d(H...A)	d(D...A)	<(DHA)
N(1A)-H(1A)...Cl(1B)	0.84	2.32	3.154(5)	173.6
N(2A)-H(2A)...N(4A)	0.84	1.86	2.580(6)	142.1
N(1B)-H(1B)...Cl(1A)	0.84	2.38	3.216(4)	172.2
N(2B)-H(2B)...N(4B)	0.85	1.90	2.597(6)	139.1
**[Re(HL^{13})$(CO)_3$].½($CHCl_3$)**				
D-H...A	d(D-H)	d(H...A)	d(D...A)	<(DHA)
N(1A)-H(1A)...Cl(1B)	0.83(8)	2.30(8)	3.124(5)	176(8)
N(2A)-H(2A)...N(4A)	0.87(8)	1.93(8)	2.625(7)	136(7)
N(4B)-H(4B)...Cl(1A)	0.77(7)	2.85(7)	3.337(5)	123(6)
N(4B)-H(4B)...N(2B)	0.77(7)	1.98(7)	2.605(7)	138(7)
N(1B)-H(1B)...Cl(1A)	0.86(7)	2.48(8)	3.331(5)	173(7)
C(1S)-H(1S)...O(32A)	1.00	2.31	3.087(10)	133.9
**[$Re_2(L^{13})_2(CO)_6$]**				
D-H...A	d(D-H)	d(H...A)	d(D...A)	<(DHA)
C(4)-H(4)...N(2)	0.95	2.31	2.880(6)	118.0
N(1)-H(1)...O(1)#1	0.85(5)	2.13(5)	2.949(5)	163(5)
Symmetry transformations: #1 -x+1/2,y-1/2,z				

Table S3 (cont.)

**[Re(HL^{11OEt})(CO)$_3$]Cl. (EtOH)**				
D-H...A	d(D-H)	d(H...A)	d(D...A)	<(DHA)
N(3)-H(3)...O(1S)#2	0.91(3)	1.91(3)	2.777(3)	159(3)
N(1)-H(1)...Cl(1)	0.82(3)	2.67(3)	3.396(2)	149(3)
N(1)-H(1)...O(1S)	0.82(3)	2.58(3)	3.244(3)	139(3)
N(2)-H(2)...Cl(1)	0.84(3)	2.32(3)	3.121(2)	160(3)
O(1)-H(1A)...Cl(1)#1	0.80(3)	2.32(3)	3.115(2)	175(3)
O(1S)-H(1S)...Cl(1)	0.75(3)	2.36(4)	3.061(2)	155(3)
C(2S)-H(2SA)...O(33)#3	0.98	2.57	3.396(3)	142.1
Symmetry transformations: #1 x,y-1,z #2 -x+1,-y+1,-z+2 #3 x-1,y,z+1				
**[Re(HL^{13OEt})(CO)$_3$]Br. ½(H$_2$O)**				
D-H...A	d(D-H)	d(H...A)	d(D...A)	<(DHA)
N(2A)-H(2A)...Br(1A^c)	0.88	3.01	3.582(10)	124.4
N(2A)-H(2A)...O(1S)	0.88	2.24	2.980(10)	141.9
N(1A)-H(1A)...Br(1A^c)	0.88	2.64	3.314(11)	133.8
N(2B)-H(2B)...Br(1B^c)	0.84(7)	2.75(8)	3.464(7)	144(6)
N(1B)-H(1B)...Br(1B^c)	0.70(7)	2.60(7)	3.293(8)	172(8)
**[Re(L^{13OMe})(CO)$_3$]**				
D-H...A	d(D-H)	d(H...A)	d(D...A)	<(DHA)
N(1)-H(1)...S(1)#1	0.88	2.74	3.482(4)	143.0
N(3)-H(3)...N(2)#2	1.00	2.18	3.067(6)	147.0
O(1A^b)-H(1A^b)...O(1A^b)#3	0.84	1.92	2.63(3)	141.5
Symmetry transformations: #1 -x+1,-y,-z+1 #2 -x+2,-y,-z+1 #3 -x+1,-y,-z+2				
[Re$_3$(L^{23}) (HL^{23})(CO)$_9$].3(CHCl$_3$)				
D-H...A	d(D-H)	d(H...A)	d(D...A)	<(DHA)
N(1A)-H(1A)...Cl(1A)	0.84	2.37	3.187(7)	164.2
N(1B)-H(1B)...Cl(1C)	0.84	2.43	3.264(9)	168.6

Figure S2. The structure of the formamidrazonium cation

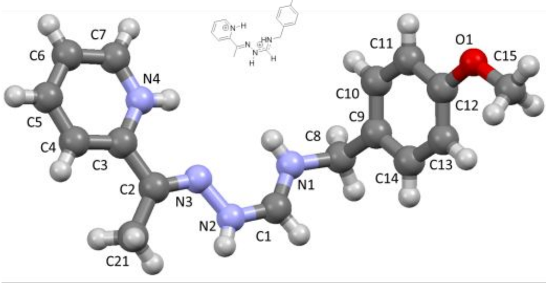

E,E,Z,E **[FMH]$_2$(SO$_4$)(HSO$_4$)$_2$.3H$_2$O**

Figure S3. The asymmetric unit in the structures of the acetone (a) and chloroform (b) solvates of [ReCl(HL^{13})(CO)$_3$] (13a) emphasizing the different association by H-bonding. Detail of the molecular structure of the acetone (c, only one of the two molecules of the asymmetric unit is shown) and chloroform (d, both molecules are included) solvate complexes**.**

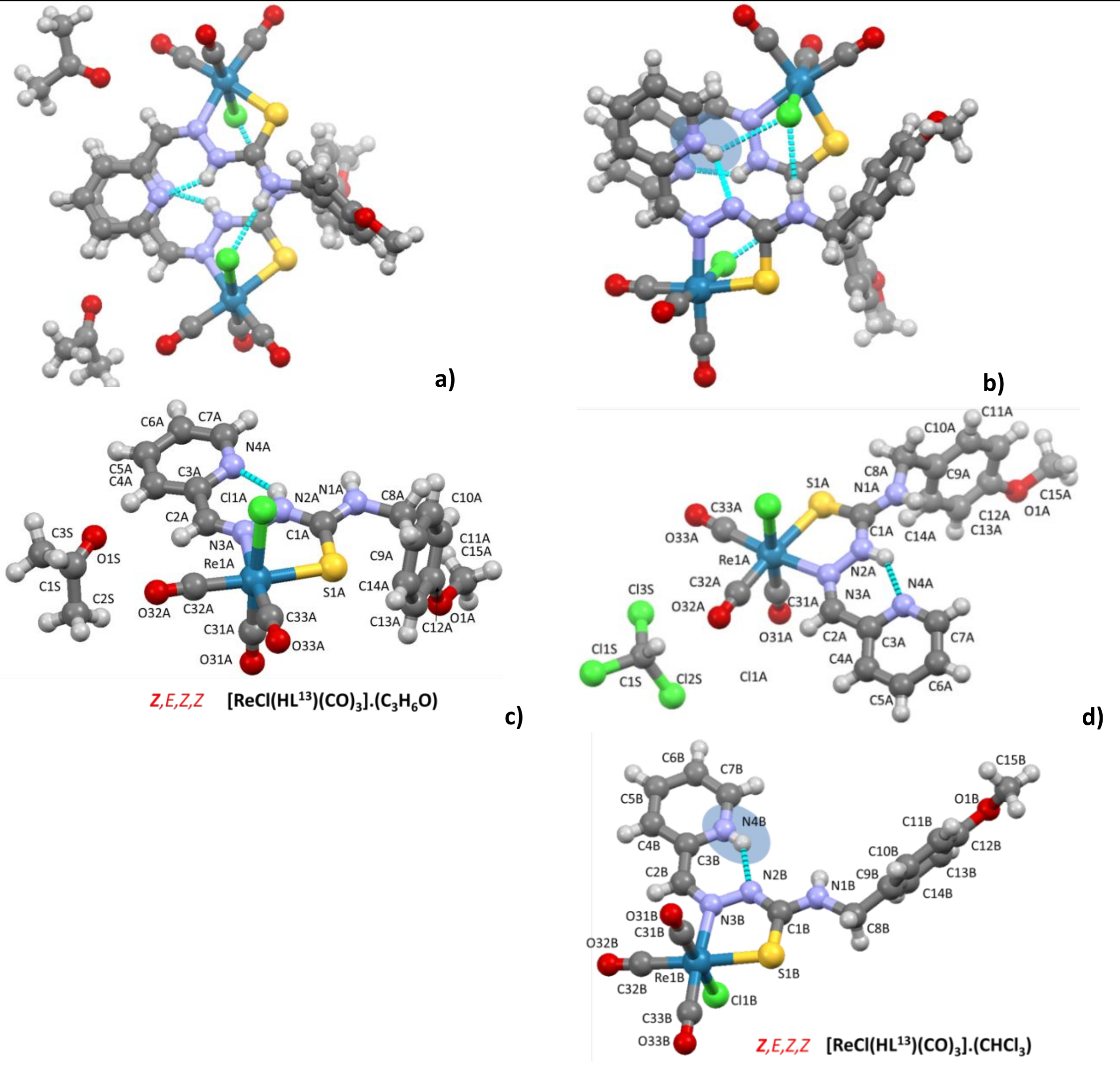

Figure S4. Molecular structures of [Re(HL^{13OEt})(CO)$_3$]Br.½H_2O (where only one of the two molecules of the asymmetric unit is depicted

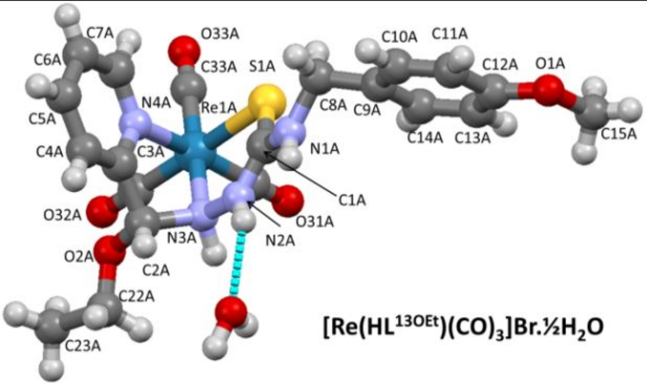

Synthesis of pyridine thiosemicarbazone ligands

The corresponding thiosemicarbazide TSZ^n and pyridine derivative xPy, were dissolved and the mixture refluxed, occasionally after added some drops of acetic acid. Then the solution was concentrated under vacuum to almost dryness and it was added diethyl ether to recover its initial volume before storing at 4 °C. Solid formed was filtered off, washed with the corresponding solvent and vacuum dried on $CaCl_2$/KOH. Reagents amounts and synthetic conditions are collected in Table S3.

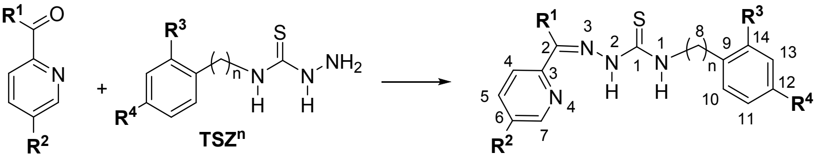

	HL^{11}	HL^{12}	HL^{13}	HL^{21}	HL^{22}	HL^{23}	HL^{24}	HL^{33}	HL^{34}
R^1=	H	H	H	Me	Me	Me	Me	Me	Me
R^2=	H	H	H	H	H	H	H	OH	OH
R^3=	H	F	H	H	F	H	H	H	H
R^4=	OH	OH	OMe	OH	OH	OMe	OH	OMe	OH
n=	0	0	1	0	0	1	1	1	1

Table S3. Details of synthetic conditions used for the synthesis of the ligands

	TSZ^n (mg)	*xPy (mL)*	*Solvent (mL)*	*Reflux*	*HAc*
HL^{11}	445 (2.4)	0.3 (3.2)	MeOH (5)	6 h	50 µL
HL^{12}	202 (1.0)	0.2 (2.1)	EtOH (20)	3 h	150 µL
HL^{13}(Z)	314 (1.5)	0.2 (2.1)	MeOH (10)	8 h	100 µL
HL^{13}(E)	150 (0.7)	0.1 (1.1)	$CHCl_3$ (10)	5 h	---
HL^{21}	330 (1.8)	0.8 (7.1)	$CHCl_3$ (20)	15 h	150 µL
HL^{22}	163 (0.8)	0.15 (1.4)	MeOH (10)	22 h	—
HL^{24}	162 (0.8)	0.15 (1.4)	MeOH (10)	22 h	—
HL^{23}	205 (1.0)	0.2 (1.8)	$CHCl_3$ (20)	5 mL	100 µL
HL^{33}	152 (0.7)	102 mg (0.7)	MeOH (15)	24 h	—
HL^{34}	77 (0.4)	54 mg (0.4)	MeOH (10)	22 h	—

HL^{11}

$HL^{11}\cdot 7/6(CH_3OH)$: Yield: 0.6 g (92 %). M.p.: 208 °C. $C_{13}H_{12}N_4OS\cdot 7/6(CH_3OH)$ (309.4): calcd. C 54.9, H 5.4, N 18.1, S 10.3; found. C 54.6, H 5.1, N 18.5, S 10.1 %. MS-ESI [m/z (%)]: 273 (100) |M+H|+. IR data (ATR, ν/cm^{-1}): 3125b ν (NH, OH); 1536m, 1509s, 1468m ν (C=N); 833m ν (C=S).

^{1}H NMR (400 MHz, DMSO-d$_6$, ppm): 11.89 (*s*, 1H, N2H), 10.07 (*s*, 1H, O2H), 9.40 (*s*, 1H, N1H), 8.57 (*d*, ^{3}J=4.9 Hz, 1H, C7H), 8.44 (*d*, ^{3}J=8.0 Hz, 1H, C4H), 8.16 (*s*, 1H, C2H), 7.83 (*td*, ^{3}J=7.5 Hz, ^{4}J=1.4 Hz, 1H, C5H), 7.38 (*ddd*, ^{3}J=7.5 Hz, ^{3}J=4.9 Hz, ^{4}J=1.1 Hz, 1H, C6H), 7.24 (*d*, ^{3}J=8.7 Hz, 2H, C10H, C14H), 6.75 (*d*, ^{3}J=8.7 Hz, 2H, C11H, C13H), 4.08 (*q*, ^{3}J=5.2 Hz, 1H, CH_3-OH), 3.17 (*d*, ^{3}J=5.2 Hz, 3H, CH_3-OH).

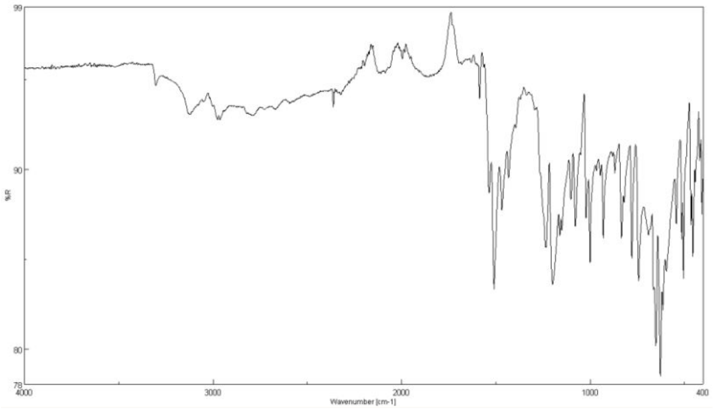

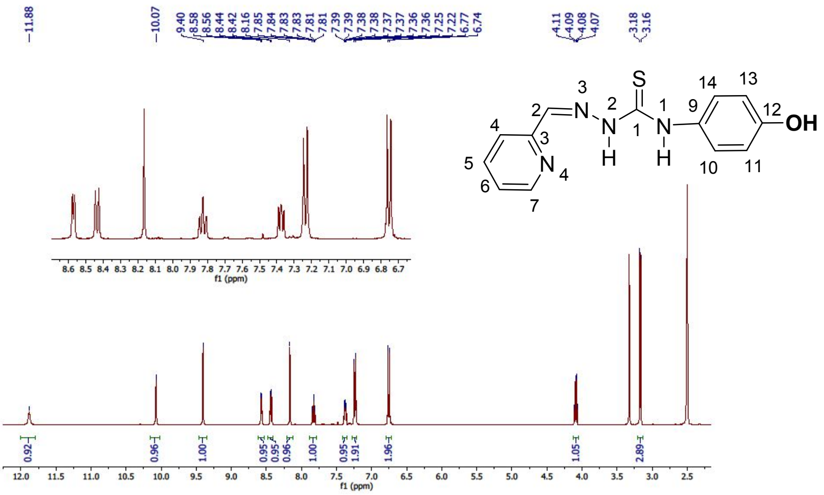

IR (top) and ^{1}H-NMR spectra in DMSO-d^{6} (bottom).

HL^{12}

$HL^{12}\cdot CH_3OH$: Yield: 0.25 g (86 %). M.p.: 213 °C. $C_{13}H_{11}FN_4OS\cdot CH_3OH$ (322.1): calcd. C 52.1, H 4.7, N 17.4, S 9.9; found. C 51.7, H 4.6, N 17.2, S 9.6 %. MS-ESI [m/z (%)]: 291 (100) |M+H|$^+$. IR data (ATR, ν/cm^{-1}): 3126b ν(NH, OH); 1542m, 1525s, 1501s, 1469m ν(C=N); 816m ν(C=S).

^{1}H NMR (400 MHz, DMSO-d$_6$, ppm): 12.04 (*s*, 1H, N2H), 9.91 (*s*, 1H, O2H), 9.90 (*s*, 1H, N1H), 8.58 (*ddd*, ^3J=4.9 Hz, ^4J=1.4 Hz, ^4J=0.9 Hz, 1H, C7H), 8.41 (*d*, ^3J=8.0 Hz, 1H, C4H), 8.16 (*s*, 1H, C2H), 7.83 (*td*, ^3J=7.5 Hz, ^4J=1.4 Hz, 1H, C5H), 7.38 (*ddd*, ^3J=7.5 Hz, ^3J=4.9 Hz, ^4J=1.1 Hz, 1H, C6H), 7.17 (*t*, ^3J=8.6 Hz, ^4J=9.5 Hz, 1H, C10H), 6.63 (*dd*, ^3J=14.5 Hz, ^4J=2.6 Hz, 1H, C13H), 6.63-6.60 (*m*, 1H, C11H), 4.08 (*q*, ^3J=5.2 Hz, 1H, CH_3-O<u>H</u>), 3.17 (*d*, ^3J=5.2 Hz, 3H, C<u>H</u>$_3$-OH).

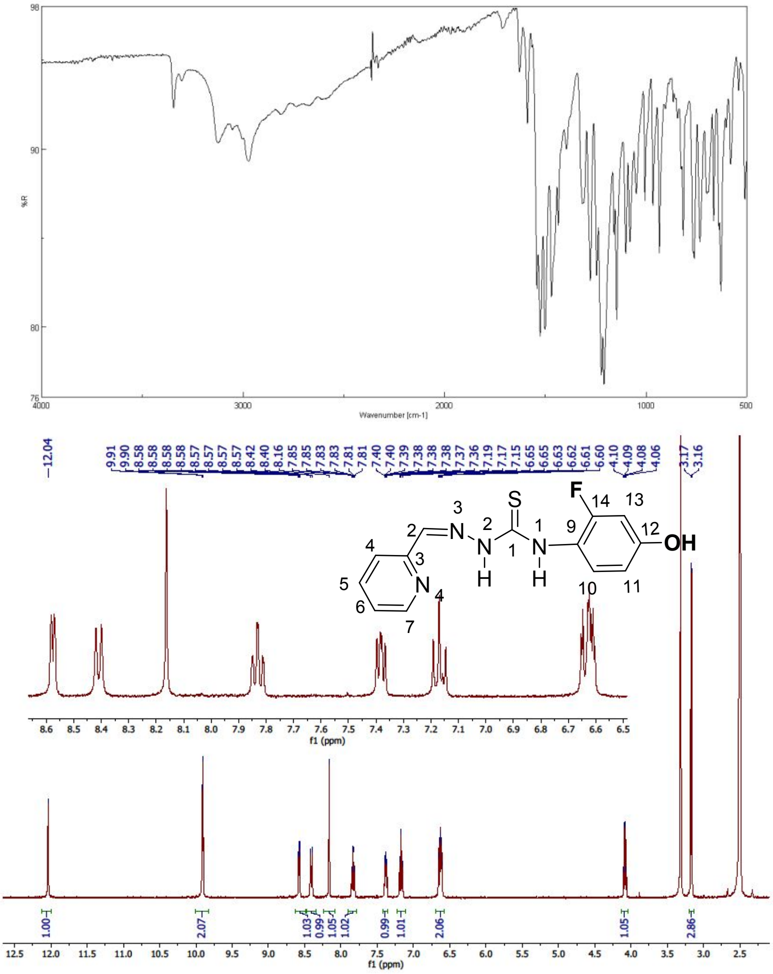

IR (top) and ^{1}H-NMR spectra in DMSO-d^6 (bottom).

HL^{13}

HL^{13}(E): Yield: 0.38 g (86 %). M.p.: 166 °C. $C_{15}H_{16}N_4OS$ (300.1): calcd. C 60.0, H 5.4, N 18.7, S 10.6; found. C 59.7, H 5.5, N 18.7, S 10.5 %. MS-ESI [m/z (%)]: 301 (100) |M+H|$^+$. IR data (ATR, ν/cm^{-1}): 3380d, 3132b ν(NH, OH); 1523vs, 1508s, 1464m, 1434m ν(C=N); 1031m ν(O-CH_3); 778m ν(C=S).

^{1}H NMR (400 MHz, DMSO-d_6, ppm): 11.76 (*s*, 1H, N2H), 9.16 (*t*, ^3J=6.2 Hz, 1H, N1H), 8.56 (*d*, ^3J=4.9 Hz, 1H, C7H), 8.27 (*d*, ^3J=8.0 Hz, 1H, C4H), 8.12 (*s*, 1H, C2H), 7.82 (*td*, ^3J=7.5 Hz, ^4J=1.4 Hz, 1H, C5H), 7.37 (*ddd*, ^3J=7.5 Hz, ^3J=4.9 Hz, ^4J=1.1 Hz, 1H, C6H), 7.30 (*d*, ^3J=8.7 Hz, 2H, C10H, C14H), 6.89 (*d*, ^3J=8.7 Hz, 2H, C11H, C13H), 4.78 (*d*, ^3J=6.2 Hz, 2H, C8H), 3.72 (*s*, 3H, C16H).

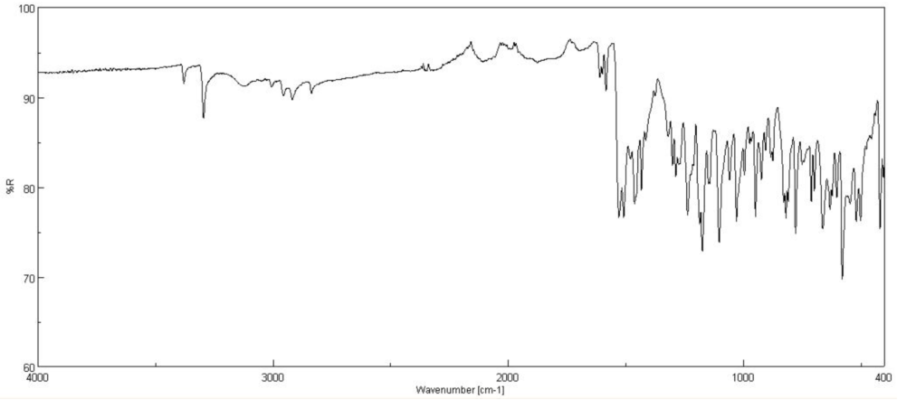

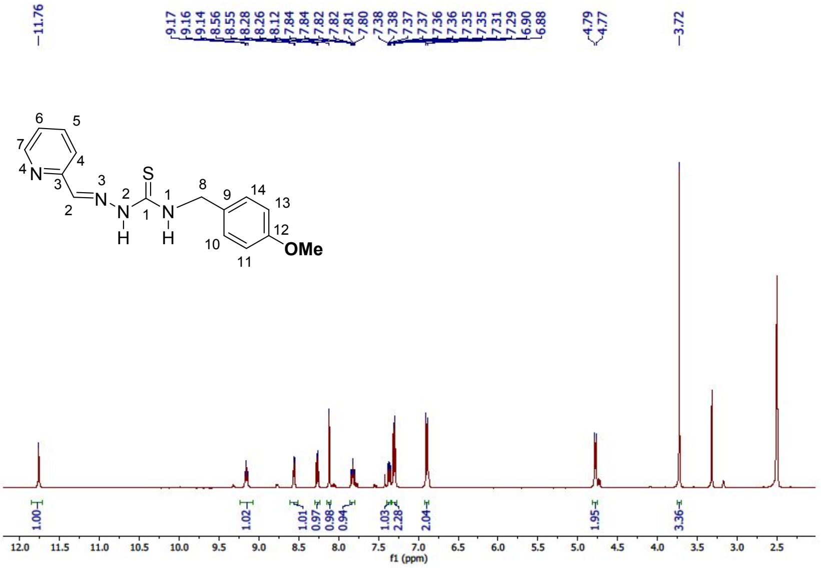

IR (top) and ^{1}H-NMR spectra in DMSO-d^6 (bottom).

HL^{13}(Z): Yield: 0.2 g (94 %). M.p.: 166 °C. $C_{15}H_{16}N_4OS$ (300.1): calcd. C 60.0, H 5.4, N 18.7, S 10.6; found. C 59.7, H 5.5, N 18.6, S 10.0 %. MS-ESI [m/z (%)]: 301 (100) $|M+H|^+$. IR data (ATR, ν/cm^{-1}): 3296m, 3117b ν(NH, OH); 1531m, 1509m, 1456m ν(C=N); 1030m ν($O-CH_3$); 820m ν(C=S).

^1H NMR (400 MHz, DMSO-d_6, ppm): 14.13 (*s*, 1H, N2H), 9.33 (*t*, ^3J=6.2 Hz, 1H, N1H), 8.78 (*d*, ^3J=4.1 Hz, 1H, C7H), 8.07 (*td*, ^3J=7.8 Hz, ^4J=1.8 Hz, 1H, C5H), 7.78 (*d*, ^3J=7.9 Hz, 1H, C4H), 7.55 (*dd*, ^3J=7.5 Hz, ^3J=4.9 Hz, 1H, C6H), 7.42 (*s*, 1H, C2H), 7.30 (*d*, ^3J=8.6 Hz, 2H, C10H, C14H), 6.88 (*d*, ^3J=8.5 Hz, 2H, C11H, C13H), 4.73 (*d*, ^3J=6.2 Hz, 2H, C8H), 3.72 (*s*, 3H, C16H).

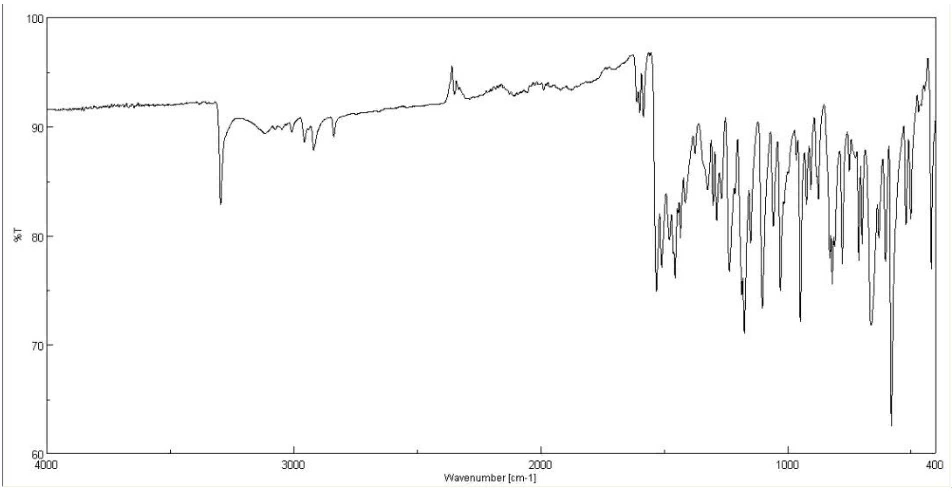

A)

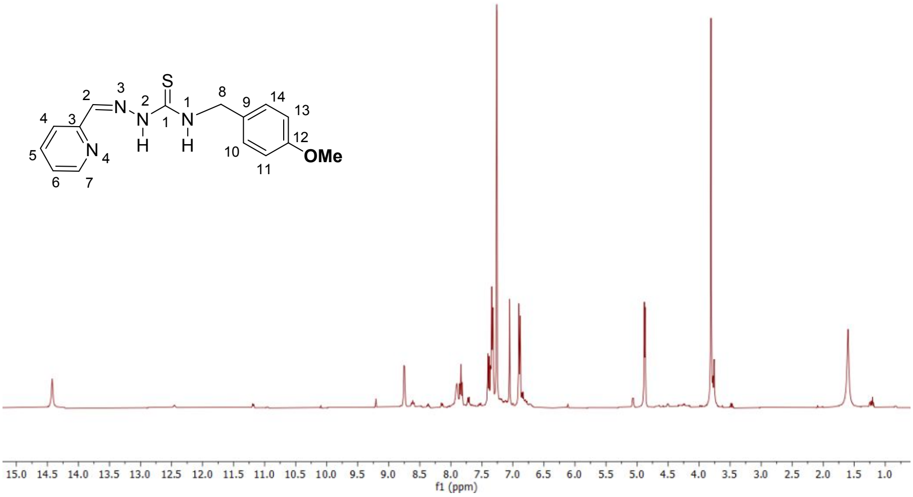

B)

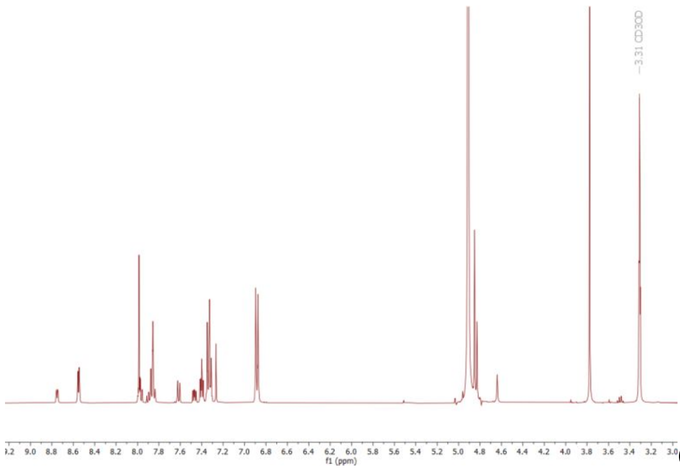

C)

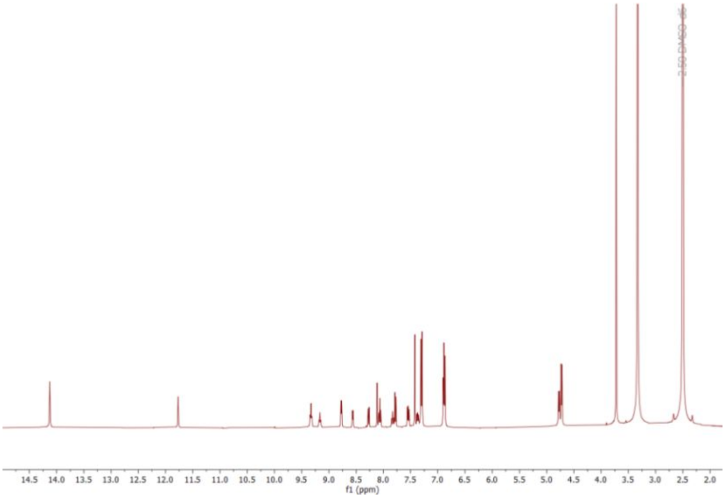

D)

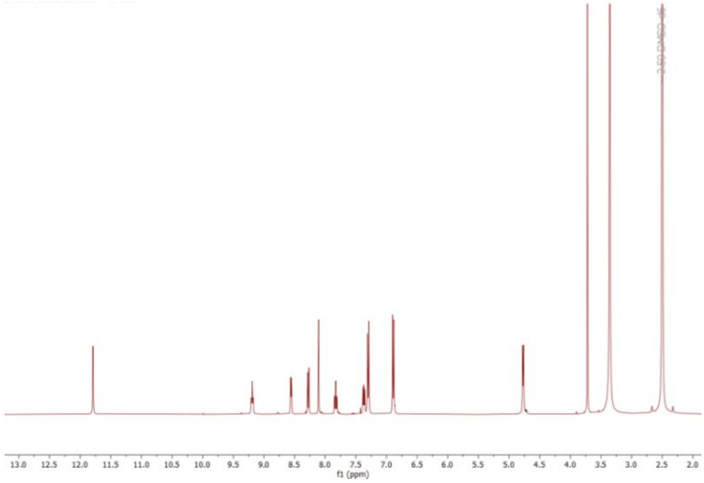

E)

IR (A) and ^{1}H-NMR in $CDCl_3$ (B), MeOH-d_4 (C), fresh solution in DMSO-d_6 (D) and the same solvent after 2 days (E)

HL^{21}

HL^{21}: Yield: 0.34 g (66 %). M.p.: 223 °C. $C_{14}H_{14}N_4OS$ (286.1): calcd. C 58.7, H 4.9, N 19.6, S 11.1; found. C 58.6, H 5.0, N 19.4, S 10.7 %. MS-ESI [m/z (%)]: 287 (100) $|M+H|^+$. IR data (ATR, ν/cm^{-1}): 3130b ν(NH, OH); 1517s, 1496s, 1468m, 1427m ν(C=N); 817m ν(C=S).

^{1}H NMR (400 MHz, DMSO-d_6, ppm): 10.50 (*s*, 1H, N2H), 10.01 (*s*, 1H, O2H), 9.40 (*s*, 1H, N1H), 8.59 (*d*, ^3J=4.8 Hz, 1H, C7H), 8.55 (*d*, ^3J=8.1 Hz, 1H, C4H), 7.79 (*td*, ^3J=7.5 Hz, ^4J=1.7 Hz, 1H, C5H), 7.39 (*dd*, ^3J=6.4 Hz, ^3J=4.8 Hz, 1H, C6H), 7.24 (*d*, ^3J=8.7 Hz, 2H, C10H, C14H), 6.76 (*d*, ^3J=8.7 Hz, 2H, C11H, C13H), 2.44 (*s*, 3H, C15H).

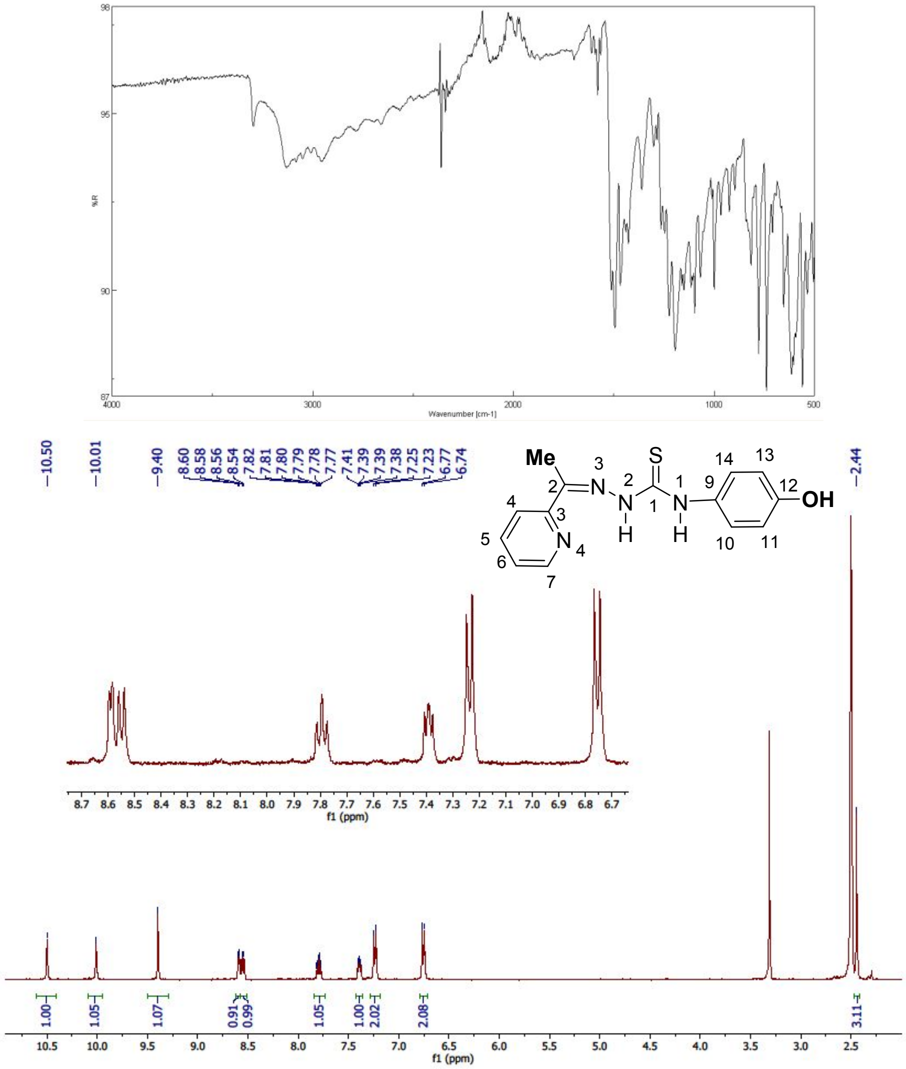

IR (top) and ^{1}H-NMR spectra in DMSO-d_6 (bottom).

HL^{22}

HL^{22}: Yield: 0.14 g (57 %). M.p.: 211 °C. $C_{14}H_{13}FN_4OS$ (304.1): calcd. C 55.3, H 4.3, N 18.4, S 10.5; found. C 55.0, H 4.5, N 18.4, S 10.4 %. MS-ESI [m/z (%)]: 305 (100) $|M+H|^+$. IR data (ATR, ν/cm^{-1}): 3147b ν(NH, OH); 1523vs, 1496s, 1470s, 1433m ν(C=N); 775m ν(C=S).

^{1}H NMR (400 MHz, DMSO-d_6, ppm): 11.71 (*s*, 1H, N2H), 9.89 (*s*, 1H, O2H), 9.85 (*s*, 1H, N1H), 8.59 (*ddd*, ^{3}J=4.9 Hz, ^{4}J=1.7 Hz, ^{4}J=0.9 Hz, 1H, C7H), 8.56 (*d*, ^{3}J=8.1 Hz, 1H, C4H), 7.79 (*td*, ^{3}J=7.8 Hz, ^{4}J=1.7 Hz, 1H, C5H), 7.39 (*ddd*, ^{3}J=7.4 Hz, ^{3}J=4.9 Hz, ^{4}J=1.1 Hz, 1H, C6H), 7.20 (*t*, ^{3}J=8.7 Hz, ^{4}J=9.4 Hz, 1H, C10H), 6.63 (*dd*, ^{3}J=14.4 Hz, ^{4}J=2.5 Hz, 1H, C13H), 6.65-6.61 (*m*, 1H, C11H), 2.45 (*s*, 3H, C15H).

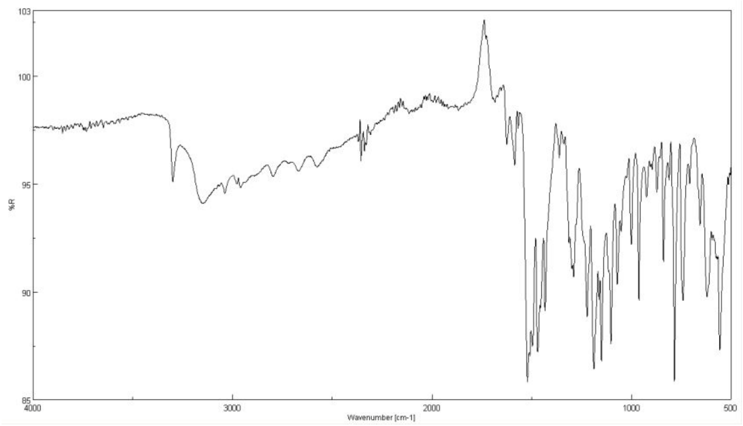

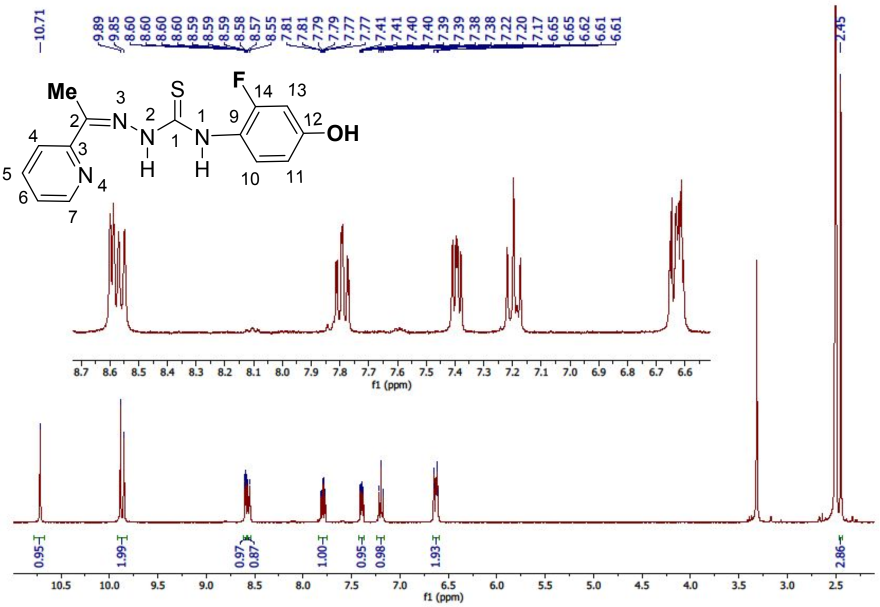

IR (top) and ^{1}H-NMR spectra in DMSO-d_6 (bottom).

HL^{23}

HL^{23}: Yield: 0.20 g (66 %). M.p.: 121 °C. $C_{16}H_{18}N_4OS$ (314.1): calcd. C 61.1, H 5.8, N 17.8, S 10.2; found. C 61.3, H 5.7, N 17.7, S 10.0 %. MS-ESI [m/z (%)]: 315 (100) $|M+H|^+$. IR data (ATR, ν/cm^{-1}): 3187b ν(NH, OH); 1539s, 1508s, 1467m, 1428 ν(C=N); 1033s ν(O-CH_3); 815m ν(C=S).

^{1}H NMR (400 MHz, DMSO-d$_6$, ppm): 11.39 (*s*, 1H, N2H), 9.12 (*t*, ^3J=6.2 Hz, 1H, N1H), 8.58 (*ddd*, ^3J=4.8 Hz, ^4J=1.7 Hz, ^4J=1.0 Hz, 1H, C7H), 8.41 (*d*, ^3J=8.1 Hz, 1H, C4H), 7.80 (*td*, ^3J=7.8 Hz, ^4J=1.7 Hz, 1H, C5H), 7.38 (*ddd*, ^3J=7.4 Hz, ^3J=4.8 Hz, ^4J=1.1 Hz, 1H, C6H), 7.30 (*d*, ^3J=8.7 Hz, 2H, C10H, C14H), 6.89 (*d*, ^3J=8.7 Hz, 2H, C11H, C13H), 4.81 (*d*, ^3J=6.2 Hz, 2H, C8H), 3.72 (*s*, 3H, C16H), 2.40 (*s*, 3H, C15H).

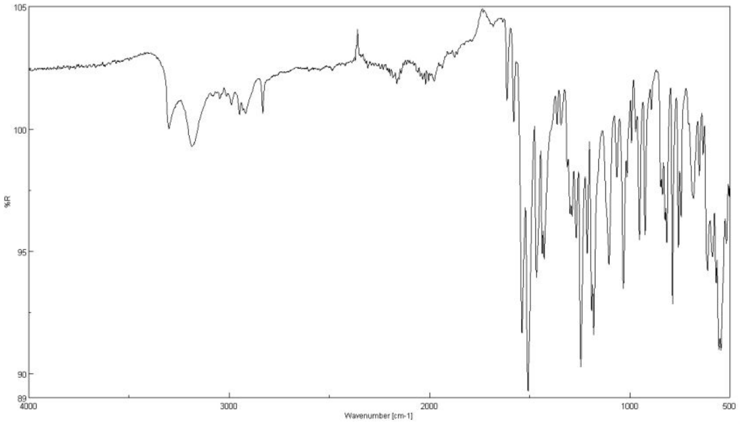

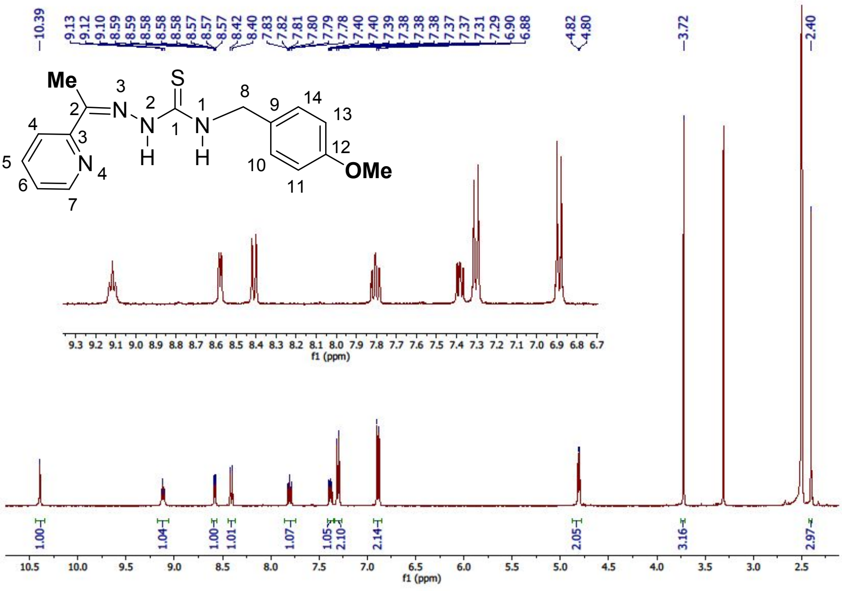

IR (top) and ^{1}H-NMR spectra in DMSO-d$_6$ (bottom).

HL^{24}

HL^{24}: Yield: 0.11 g (45 %). M.p.: 172 °C. $C_{15}H_{16}N_4OS$ (300.1): calcd. C 60.0, H 5.4, N 18.7, S 10.7; found. C 59.9, H 5.5, N 18.4, S 10.4 %. MS-ESI [m/z (%)]: 301 (100) |M+H|$^+$. IR data (ATR, ν/cm^{-1}): 3195b ν(NH, OH); 1540m, 1506s, 1470s, 1432m ν(C=N); 816m ν(C=S).

^{1}H NMR (400 MHz, DMSO-d$_6$, ppm): 10.36 (*s*, 1H, N2H), 9.25 (*s*, 1H, O2H), 9.06 (*t*, ^3J=6.0 Hz, 1H, N1H), 8.58 (*d*, ^3J=4.5 Hz, 1H, C7H), 8.40 (*d*, ^3J=8.1 Hz, 1H, C4H), 7.80 (*td*, ^3J=8.1 Hz, ^4J=1.6 Hz, 1H, C5H), 7.38 (*ddd*, ^3J=7.2 Hz, ^3J=4.5 Hz, ^4J=1.1 Hz, 1H, C6H), 7.18 (*d*, ^3J=8.4 Hz, 2H, C10H, C14H), 6.71 (*d*, ^3J=8.4 Hz, 2H, C11H, C13H), 4.76 (*d*, ^3J=6.0 Hz, 2H, C8H), 2.40 (*s*, 3H, C15H).

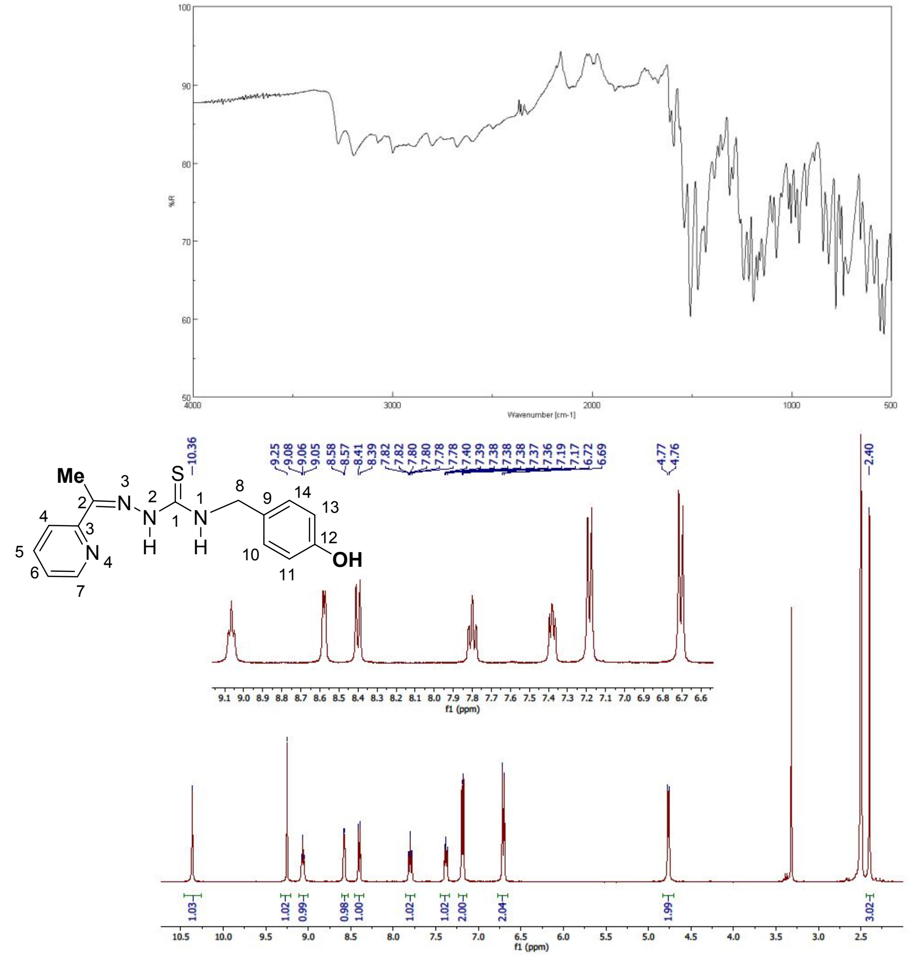

IR (top) and ^{1}H-NMR spectra in DMSO-d$_6$ (bottom).

HL^{33}

HL^{33}·1/2(H$_2$O): Yield: 0.17 g (71 %). M.p.: 168 °C. $C_{16}H_{18}N_4O_2S\cdot 1/2(H_2O)$ (339.1): calcd. C 56.6, H 5.6, N 16.5, S 9.4; found. C 56.3, H 5.2, N 16.3, S 9.0 %. MS-ESI [m/z (%)]: 331 (100) $|M+H|^+$. IR data (ATR, ν/cm^{-1}): 3184b ν(NH, OH); 1532m, 1510s, 1470s ν(C=N); 1028s ν(O-CH$_3$); 807s ν(C=S).

^{1}H NMR (400 MHz, DMSO-d$_6$, ppm): 10.26 (*s*, 1H, N2H), 10.22 (*s*, 1H, O1H), 9.03 (*t*, ^3J=6.2 Hz, 1H, N1H), 8.28 (*d*, ^3J=8.8 Hz, 1H, C4H), 8.10 (*d*, ^4J=2.6 Hz, 1H, C7H), 7.29 (*d*, ^3J=8.7 Hz, 2H, C10H, C14H), 7.16 (*dd*, ^3J=8.8 Hz, ^4J=2.6 Hz, 1H, C5H), 6.88 (*d*, ^3J=8.7 Hz, 2H, C11H, C13H), 4.79 (*d*, ^3J=6.2 Hz, 2H, C8H), 3.72 (*s*, 3H, C16H), 2.35 (*s*, 3H, C15H).

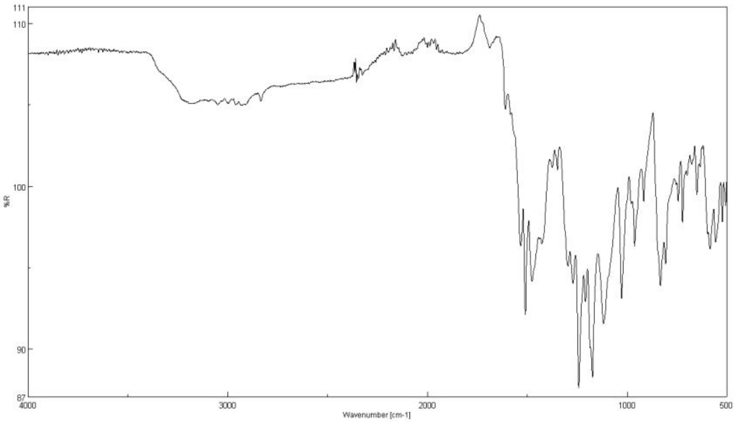

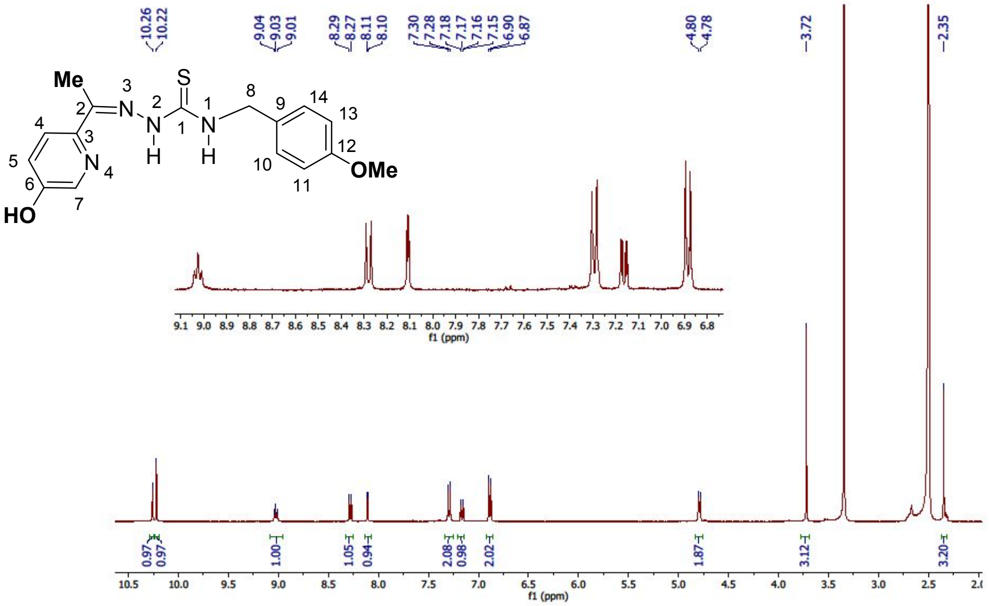

IR (top) and ^{1}H-NMR spectra in DMSO-d$_6$ (bottom).

HL^{34}

HL^{34}·1/7(C$_4$H$_{10}$O): Yield: 0.85 g (69 %). M.p.: 209 °C. $C_{15}H_{16}N_4O_2S$· 1/7($C_4H_{10}O$) (326.7): calcd. C 57.2, H 5.4, N 17.2, S 9.8; found. C 57.4, H 5.2, N 17.4, S 9.6 %. MS-ESI [m/z (%)]: 317 (100) |M+H|$^+$. IR data (ATR, ν/cm^{-1}): 3094b ν(NH, OH); 1537s, 1513s, 1478s ν(C=N); 734s ν(C=S).

^{1}H NMR (400 MHz, DMSO-d$_6$, ppm): 10.23 (*s*, 1H, N2H), 10.22 (*s,* 1H, O1H), 9.28 (*s*, 1H, O2H), 8.97 (*t*, ^3J=6.1 Hz, 1H, N1H), 8.27 (*d*, ^3J=8.8 Hz, 1H, C4H), 8.10 (*d*, ^4J=2.6 Hz, 1H, C7H), 7.17 (*d*, ^3J=8.4 Hz, 2H, C10H, C14H), 7.16 (*dd*, ^3J=8.8 Hz, ^4J=2.6 Hz, 1H, C5H), 6.70 (*d*, ^3J=8.4 Hz, 2H, C11H, C13H), 4.74 (*d*, ^3J=6.1 Hz, 2H, C8H), 2.34 (*s*, 3H, C15H).

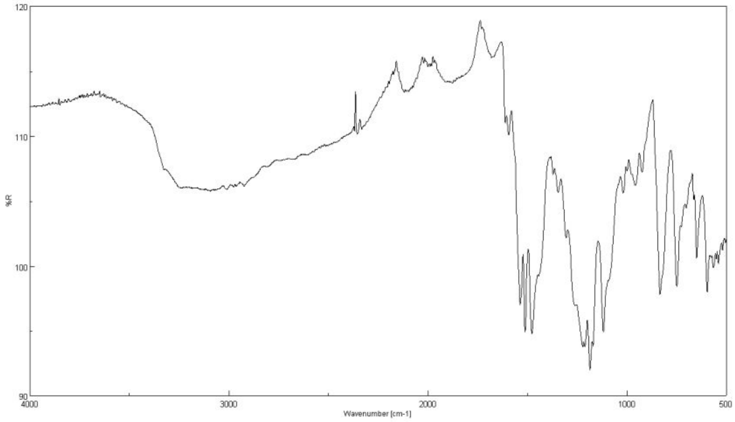

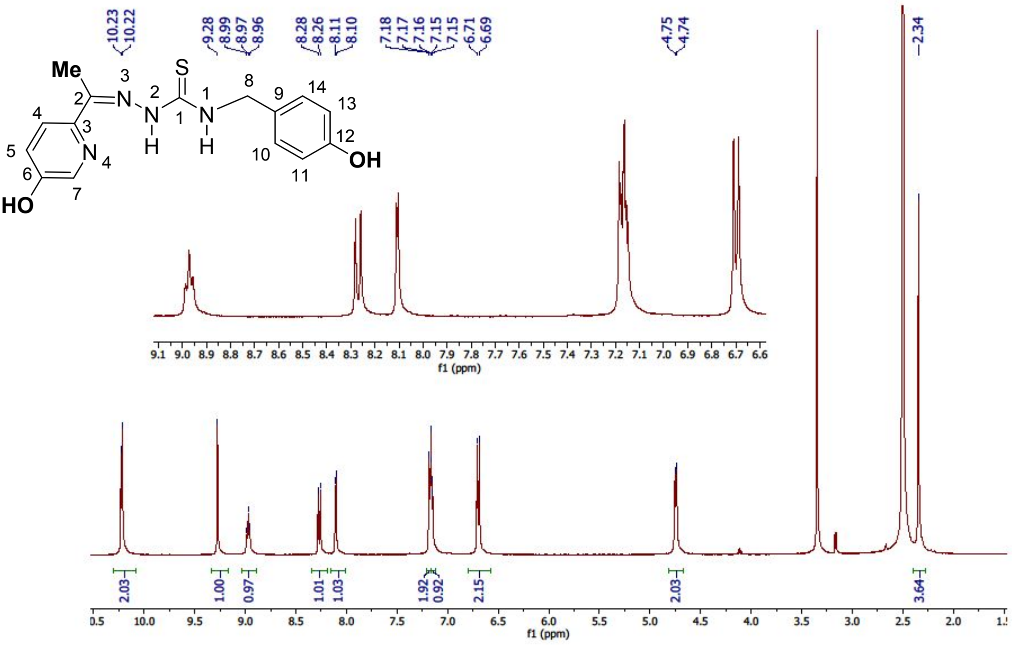

IR (top) and ^{1}H-NMR spectra in DMSO-d$_6$ (bottom).

Synthesis of [ReX(HL^n)(CO)$_3$] (X=Cl: 13a, 22a, 33a; X=Br: 13b)

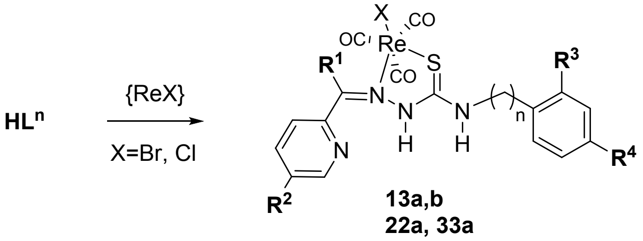

A solution of the rhenium(I) precursor (*fac*-[ReCl(CH$_3$CN)$_2$(CO)$_3$]/[ReBr(CO)$_5$]) and **HL^n** was refluxed. The resulting solution was concentrated in vacuous to half its initial volume and stored at 4 °C after adding diethyl ether. The solid formed was filtered off and vacuum dried on CaCl$_2$/KOH. Reagents amounts and synthesis conditions are collected in the following table:

Table S4. Details of synthetic conditions used for the synthesis of 13a, 22a, 33a and 13b

	mmol (mg)			***Solvent (mL)***	***Reflux***
	HL^n	ReCl(CH$_3$CN)$_2$(CO)$_3$]	[ReBr(CO)$_5$]		
**13a**	50 (0.17)	65 (0.17)		CHCl$_3$ (5)	2.5 h
**13b**	67 (0.22)		93 (0.23)	EtOH (10)	6 h
**22a**	40 (0.13)	50 (0.13)		CHCl$_3$ (5)	1 h
**33a**	49 (0.15)	59 (0.15)		CHCl$_3$ (10)	2.5 h

13a:

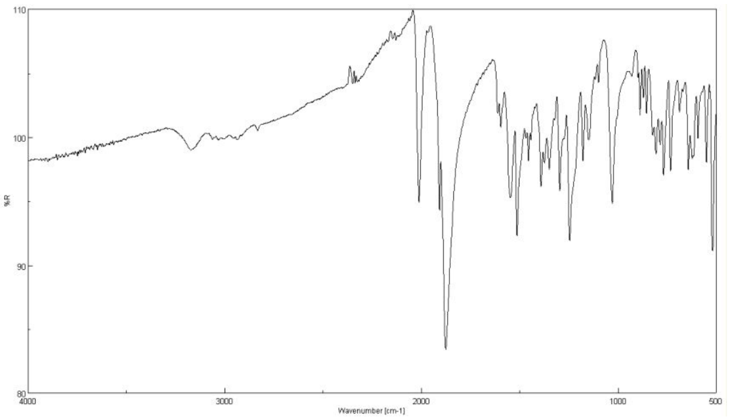

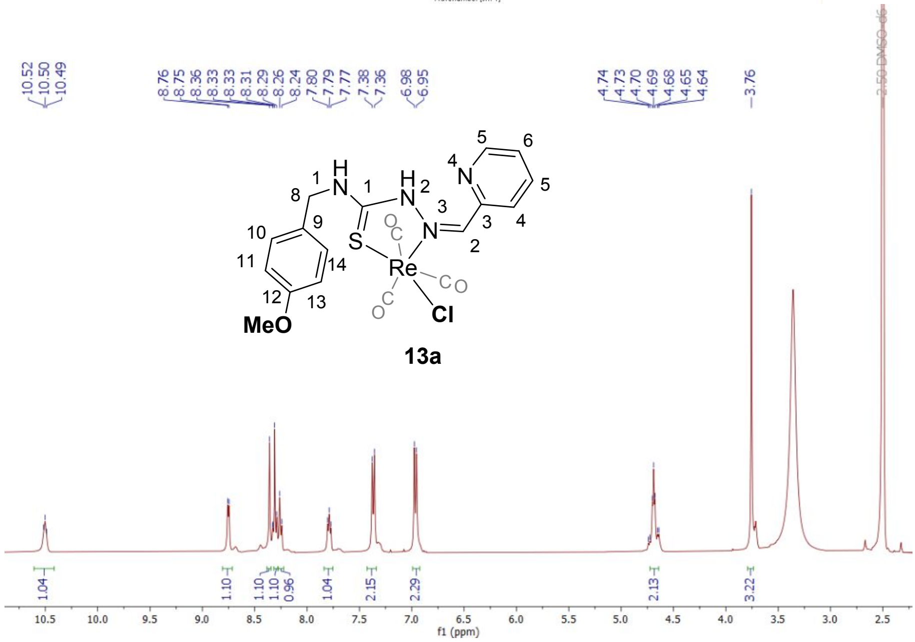

IR (top) and ^{1}H-NMR spectra in DMSO-d$_6$ (bottom).

13b·4/5(CH_3CH_2OH):

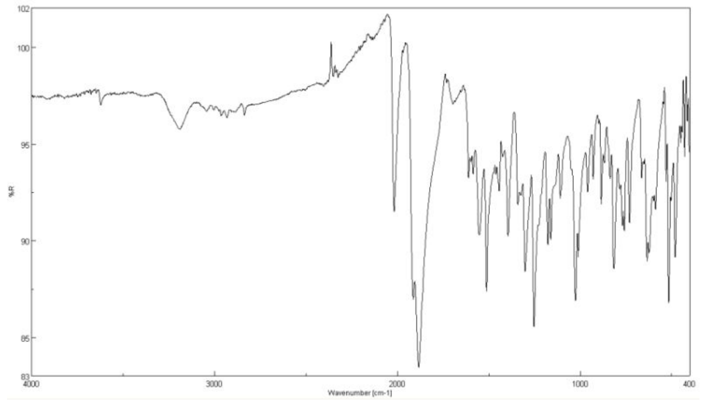

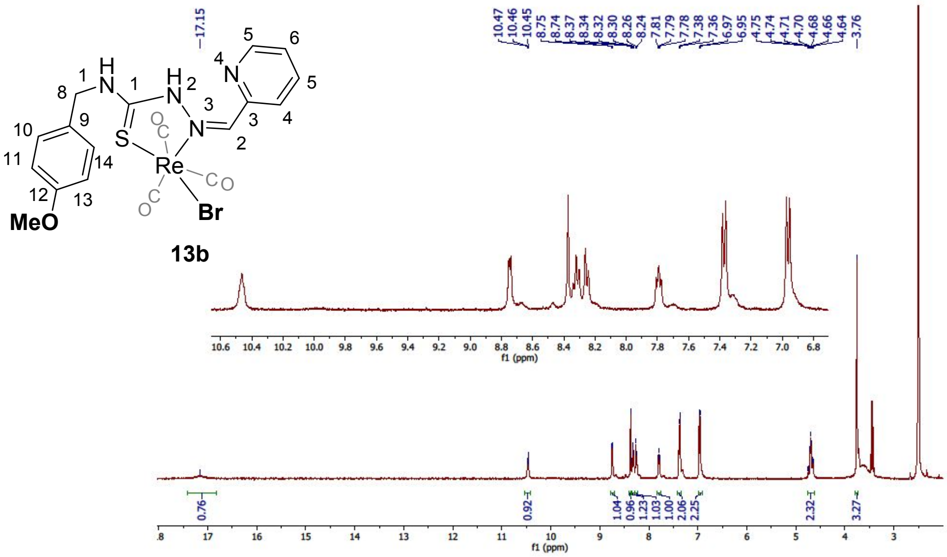

IR (top) and ^{1}H-NMR spectra in DMSO-d_6 (bottom).

22a·1/2CHCl$_3$

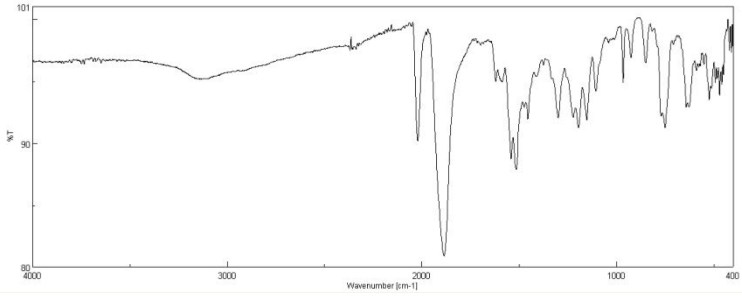

A)

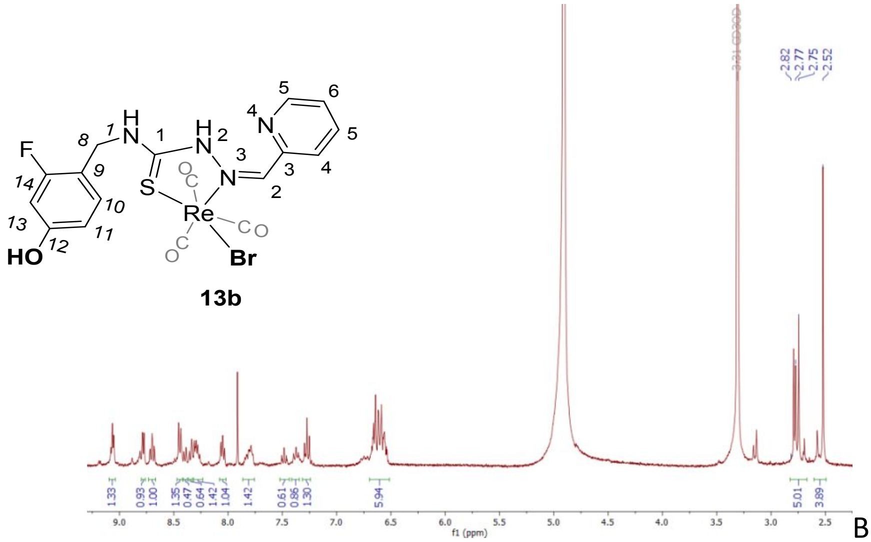

B)

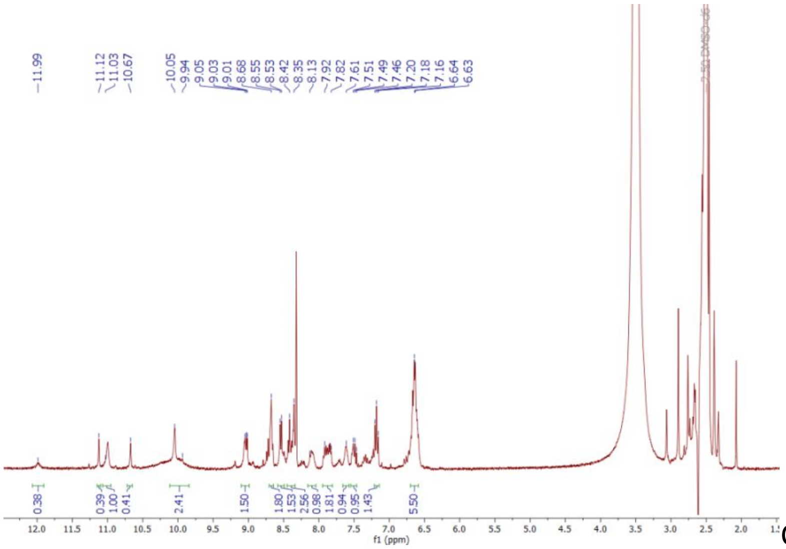

C)

IR (A) and ¹H-NMR spectra in MeOD-d$_4$ (B) and DMSO-d$_6$ (C).

33a·1/3CHCl$_3$

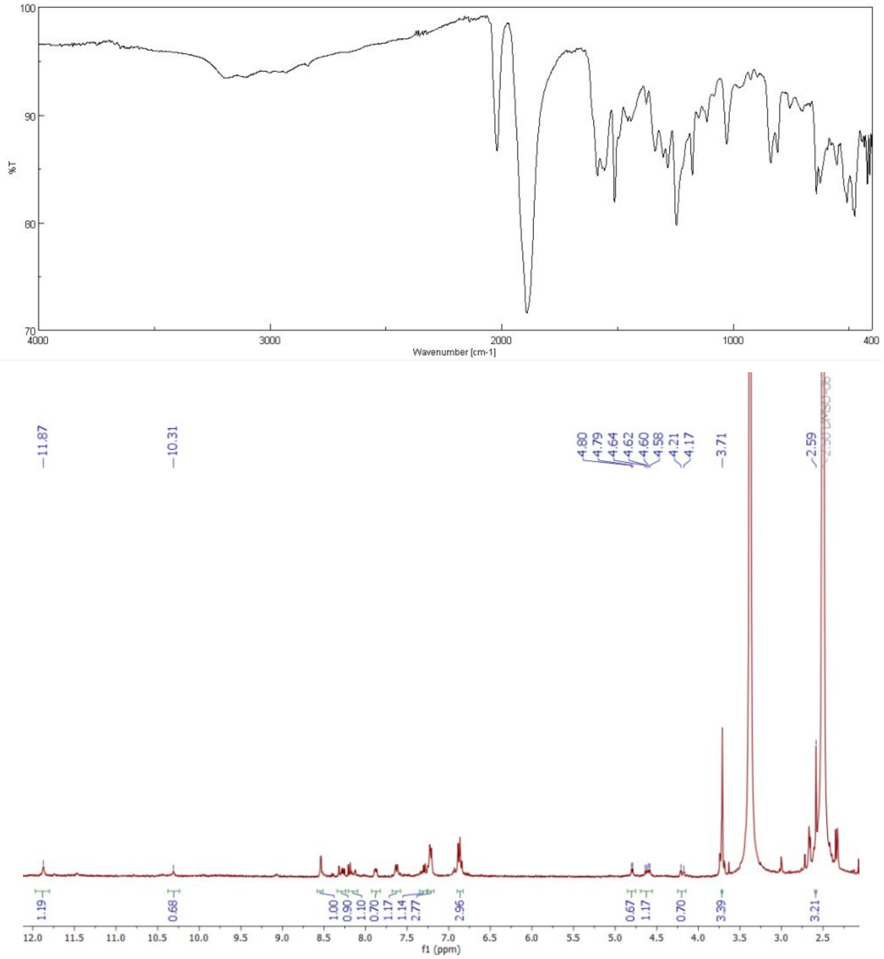

IR (top) and ^{1}H-NMR spectra in DMSO-d_6 (bottom).

Synthesis of [Re(L^{13})(CO)$_3$] (13c)

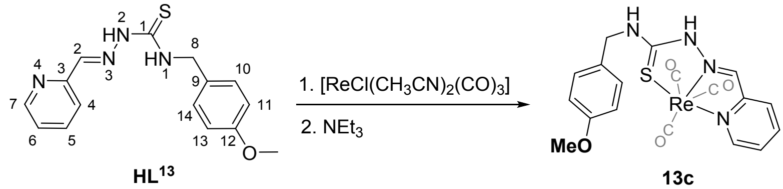

A suspension of *fac*-[ReCl(CH_3CN)$_2$(CO)$_3$] (61 mg; 0.16 mmol) and **HL^{13}** (48 mg; 0.16 mmol) in $CHCl_3$ (5 mL) was refluxed for two hours. Then it was added NEt_3 (44 µL; 0.32 mmol) and refluxed for 1 hour more. The resulting solution was concentrated under vacuum to half its initial volume and stored at 4 °C after adding diethyl ether. Solid formed was filtered off, washed with water and vacuum dried on $CaCl_2$/KOH.

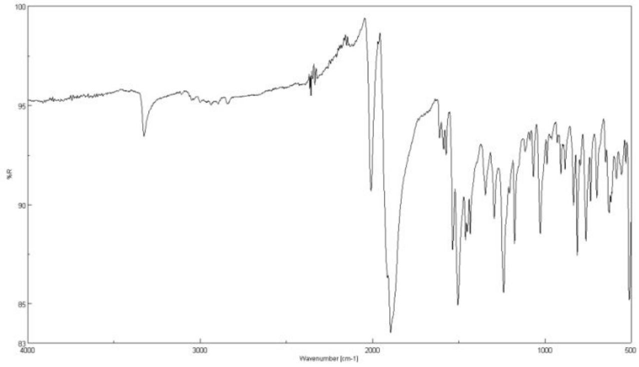

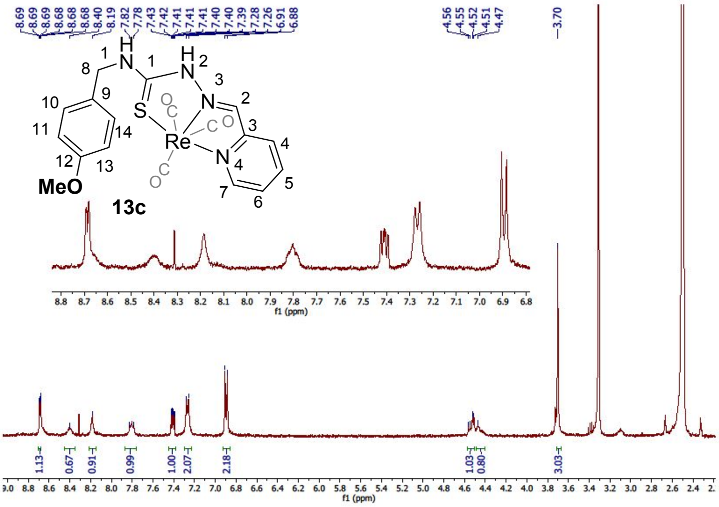

IR (top) and ^{1}H-NMR spectra in DMSO-d$_6$ (bottom).

Synthesis of the complexes [Re(L^n)(CO)$_3$] (21c-24c)

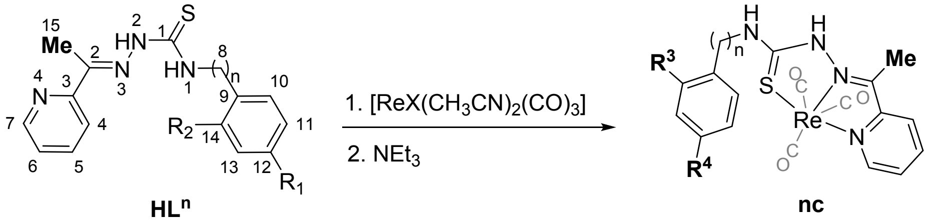

	HL^{21}	HL^{22}	HL^{23}	HL^{24}
R^4	OH 2	OH 2	OMe 2	OH 16
R^3	H	F	H	H
n	0	0	1	1

A suspension of *fac*-[ReX(CH$_3$CN)$_2$(CO)$_3$] (X=Cl for **21c**, **23c**; X=Br for **22c**, **24c**) and **HL^n** in CHCl$_3$ was refluxed for one hour. Then NEt$_3$ (0.1 mL; 0.72 mmol) was added and refluxed again. The resulting solution was concentrated under vacuum to half its initial volume and stored at 4 °C after adding diethyl ether. Solid formed was filtered off, washed with water and vacuum dried on CaCl$_2$/KOH. Reagents amounts and synthesis conditions are collected in the following table:

Table S5. Details of synthetic conditions used for the synthesis of 21c-24c

	mmol (mg)			*CHCl$_3$ (mL)*	*Reflux*
	HL^n	[ReX(CH$_3$CN)$_2$(CO)$_3$]			
		X = Cl	*X = Br*		
**21c**	55 (0.19)	76 (0.20)		10	2 h
**22c**	40 (0.13)		49 (0.13)	10	2.5 h
**23c**	63 (0.20)	77 (0.20)		7	1 h
**24c**	37 (0.12)		50 (0.12)	12	2 h

21c·2/5($C_4H_{10}O$)

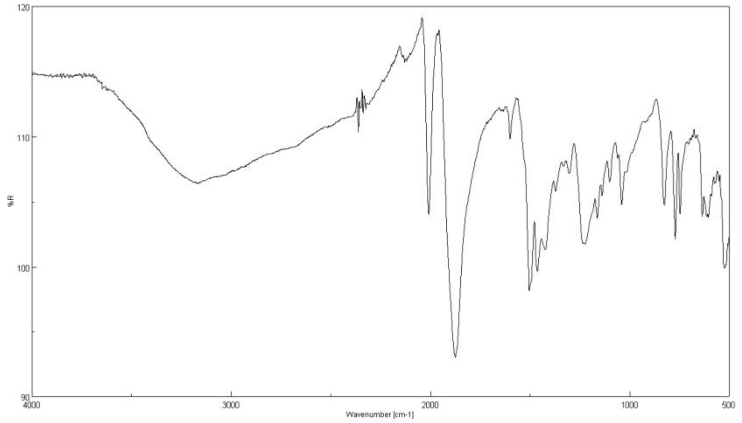

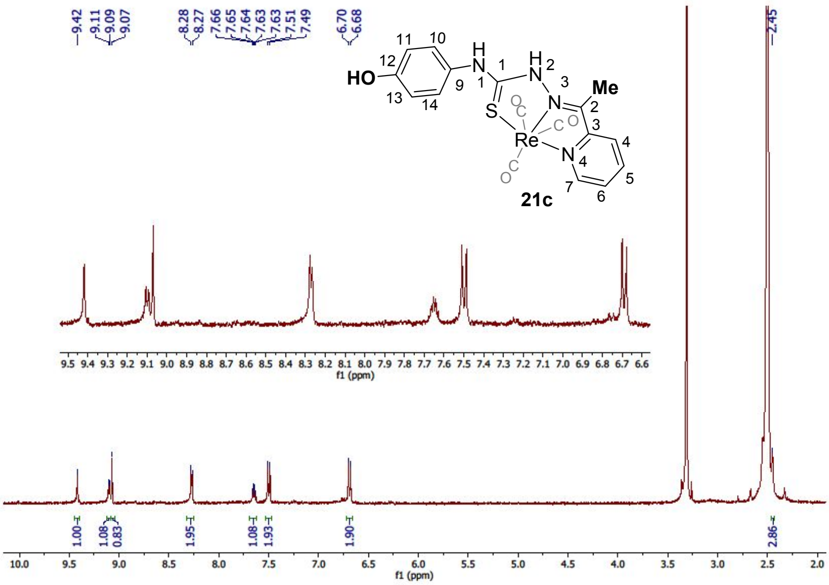

IR (top) and ^{1}H-NMR spectra in DMSO-d_6 (bottom).

22c·2/3($C_4H_{10}O$)

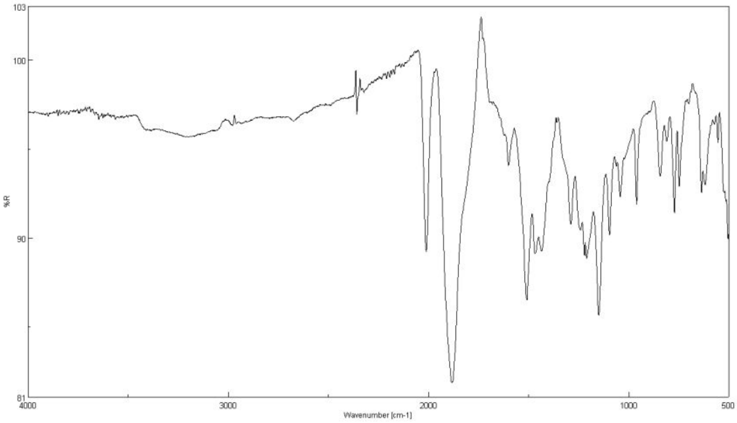

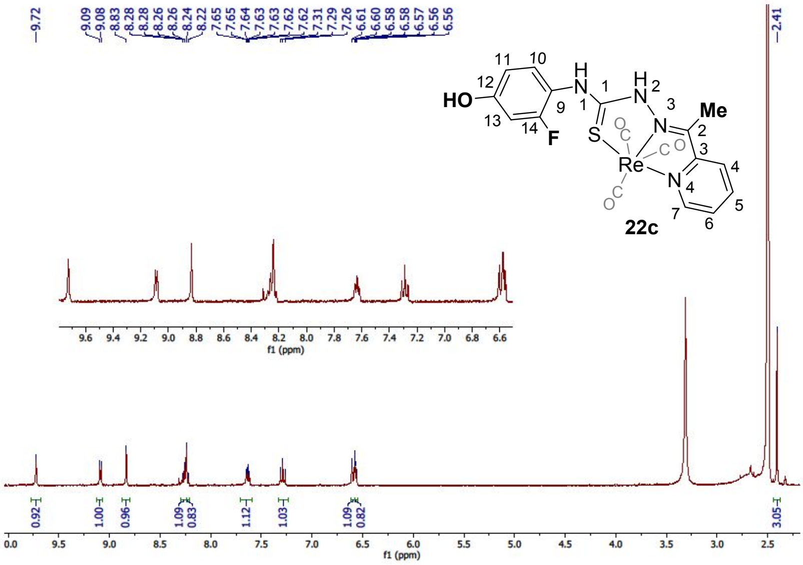

IR (top) and ^{1}H-NMR spectra in DMSO-d_6 (bottom).

23c·1/5($C_4H_{10}O$)

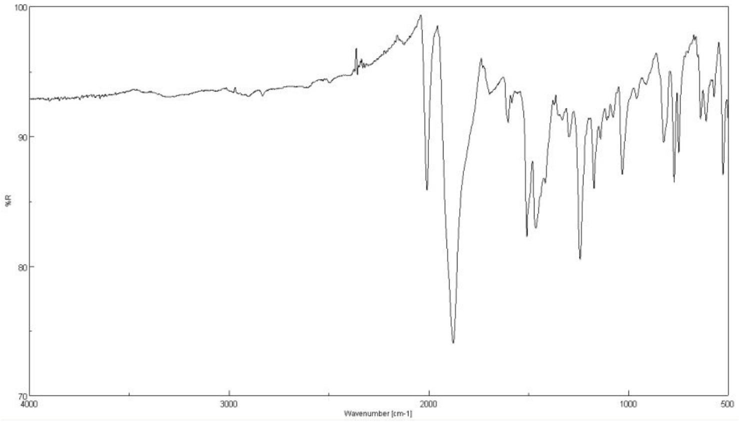

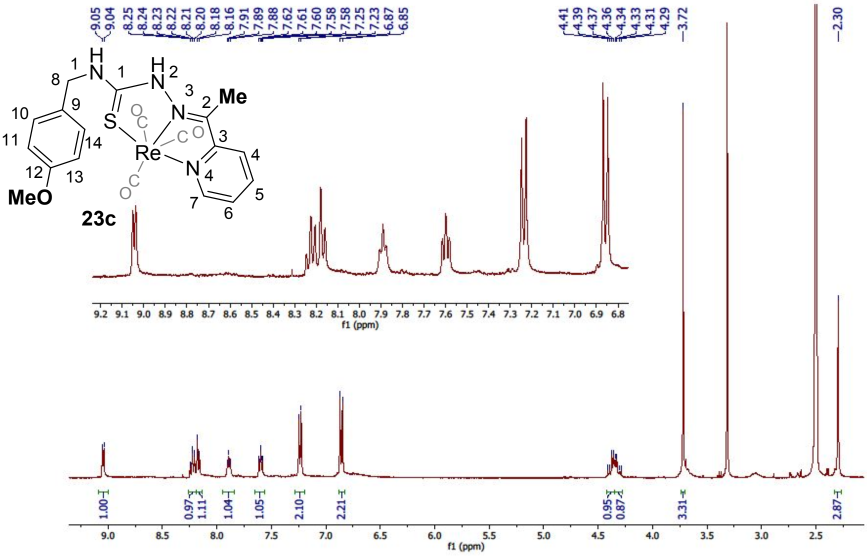

IR (top) and ^{1}H-NMR spectra in DMSO-d_6 (bottom).

24c·4/7(CHCl$_3$)

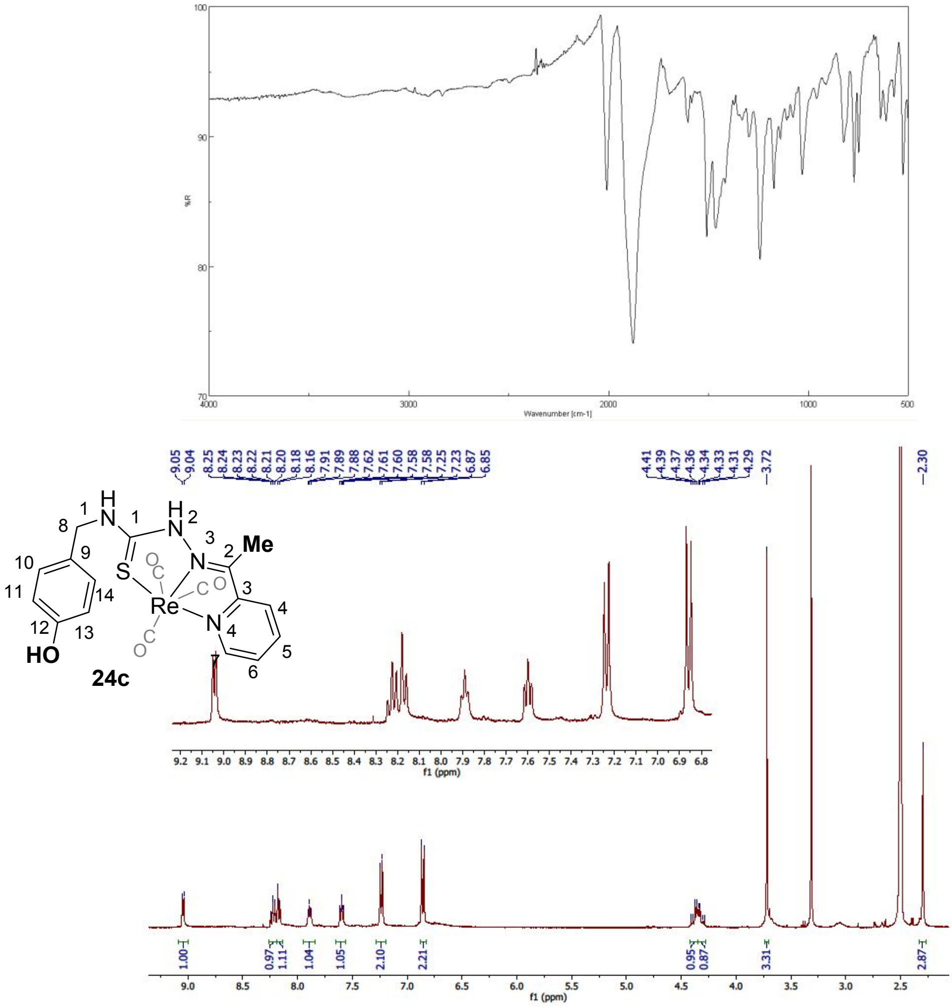

IR (top) and ^{1}H-NMR spectra in DMSO-d$_6$ (bottom).

Synthesis of [Re(L^n)(CO)$_3$] (33c and 34c)

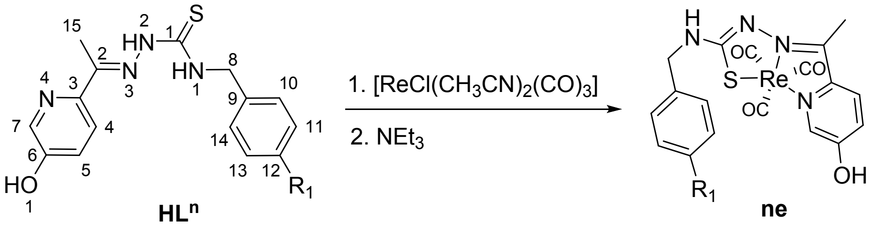

A suspension of *fac*-[ReCl(CH$_3$CN)$_2$(CO)$_3$] (60 mg; 0.15 mmol) and **HL[33]** (50 mg; 0.15 mmol) or **HL[34]** (50 mg; 0.16 mmol) in CHCl$_3$ (10 mL) was refluxed for 2 h. Then NEt$_3$ (22 µL; 0.16 mmol) was added and refluxed for 4 h more. The resulting solution was concentrated under vacuum to half its initial volume and stored at 4 °C after adding diethyl ether or tetrahydrofuran. Solid formed was filtered off, washed with water and vacuum dried on CaCl$_2$/KOH.

33c·2/5(C$_4$H$_{10}$O)

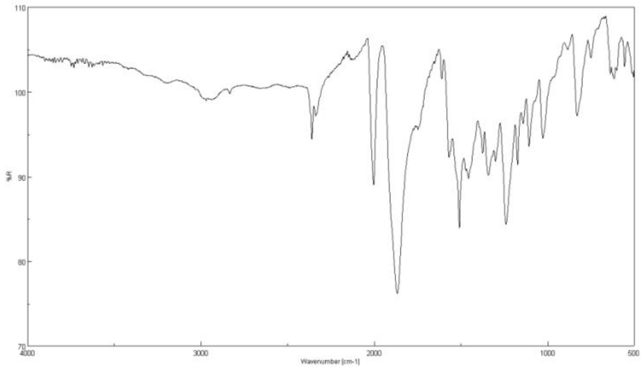

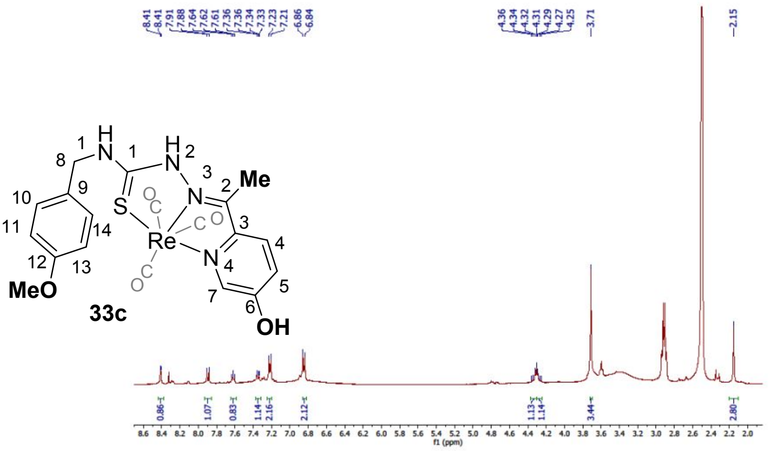

IR (top) and ^{1}H-NMR spectra in DMSO-d$_6$ (bottom).

34c·1/3(C_4H_8O)

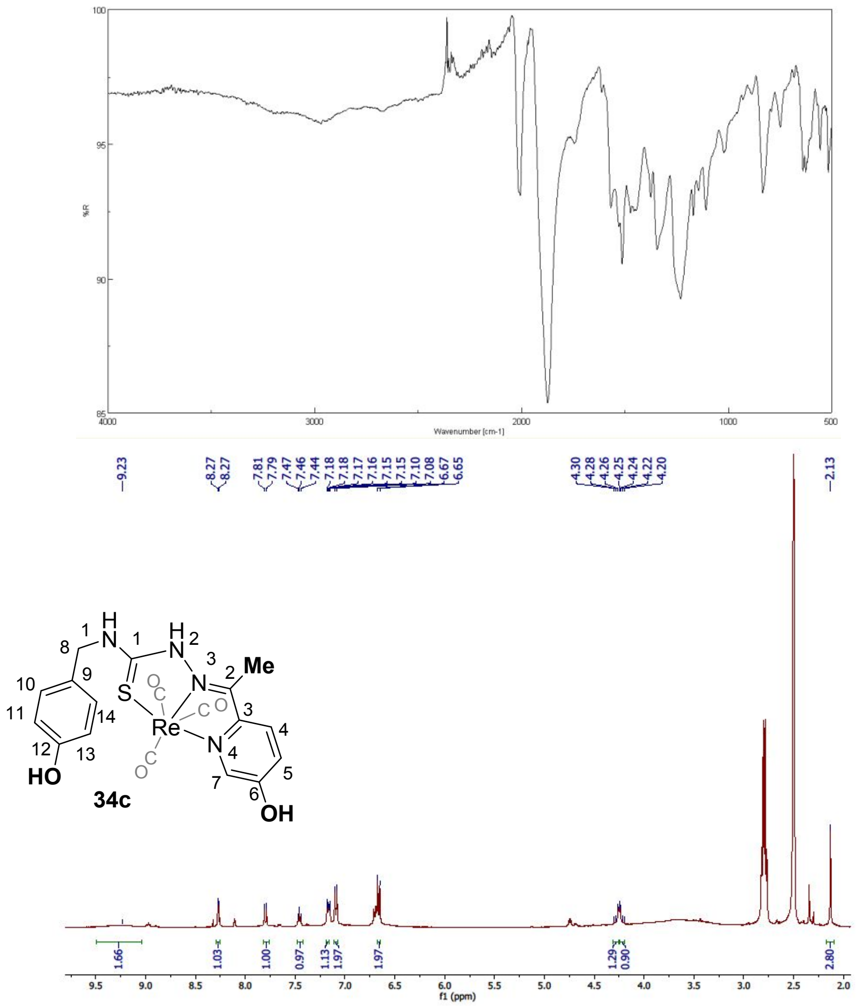

IR (top) and ^{1}H-NMR spectra in DMSO-d_6 (bottom).

Synthesis of the complexes [Re(HL$^{11\text{-}OMe}$)(CO)$_3$]Br (11d) and [Re(HL$^{13\text{-}OEt}$)(CO)$_3$]Br (13d)

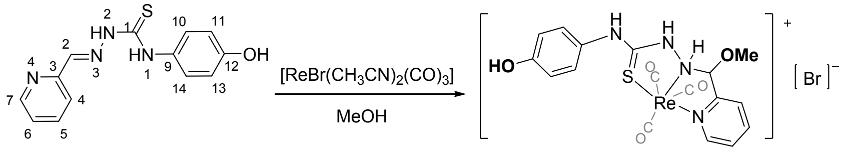

HL^{11} **11d**

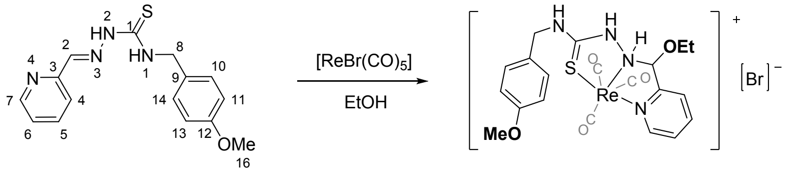

HL^{13} **13d**

A solution of the rhenium(I) precursor (*fac*-[ReBr(CH$_3$CN)$_2$(CO)$_3$]/ [ReBr(CO)$_5$]) and **HL^n** was refluxed for 6 h. The resulting solution was concentrated in vacuous to half its initial volume and stored at 4 °C after adding diethyl ether. Solid (crystalline phase in **11d**) formed was filtered off and vacuum dried on CaCl$_2$/KOH. Reagents amounts and synthesis conditions are collected in the table.

Table S6. Details of synthetic conditions used for the synthesis of 11d and 13d

	mmol (mg)		*Solvent (mL)*
	HL^n	*{ReBr}*	
**11d**	30 (0.11)	45 (0.10)	MeOH (10)
**13d**	67 (0.22)	93 (0.23)	EtOH (10)

11d:

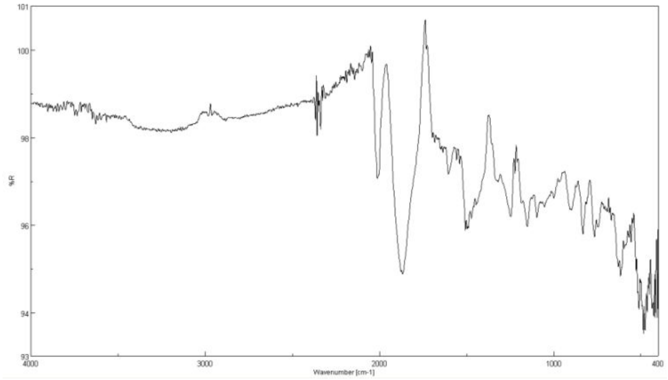

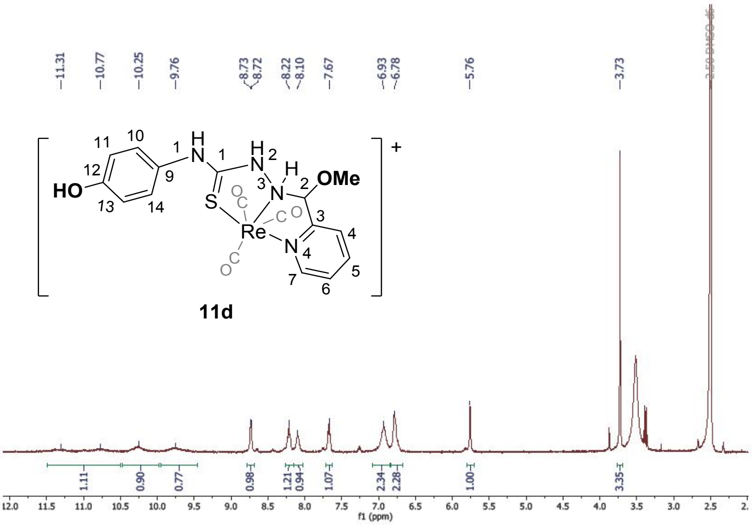

IR (top) and ^{1}H-NMR spectra in DMSO-d$_6$ (bottom).

13d 4/5(H_2O):

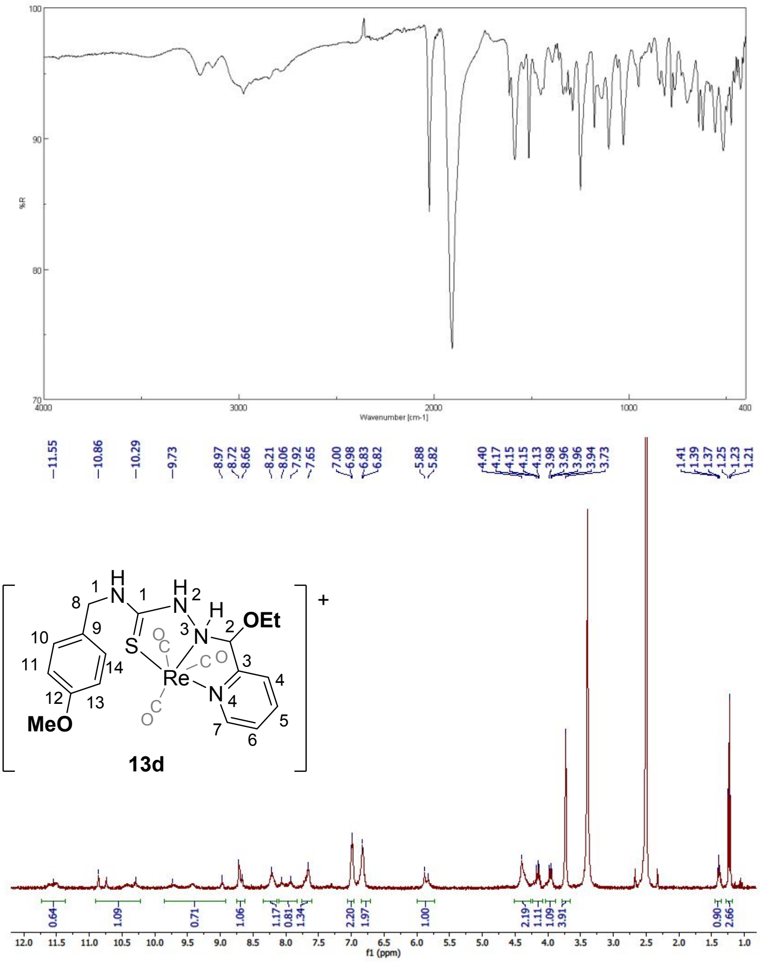

IR (top) and ^{1}H-NMR spectra in DMSO-d_6 (bottom).

Synthesis of [Re(L$^{11\text{-}OMe}$)(CO)$_3$] (11e)

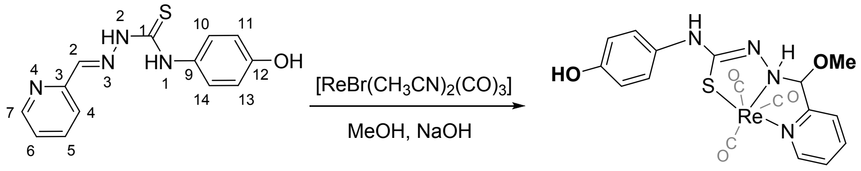

HL^{11} **11e^{Me}**

fac-[ReCl(CH$_3$CN)$_2$(CO)$_3$] (89 mg, 0.15 mmol) and **HL^{11}** (42 mg; 0.15 mmol) were mixed on MeOH (10 mL) and the solution refluxed for two hours. Then it was added a suspension of NaOH (9 mg; 0.23 mmol) in 3 mL of the same solvent and refluxed for 1 hour more. The resulting solution was concentrated to half its initial volume and stored at 4 °C after adding chloroform. Crystalline phase formed was filtered off, washed with water and vacuum dried on CaCl$_2$/KOH.

11e·3/4(CHCl$_3$):

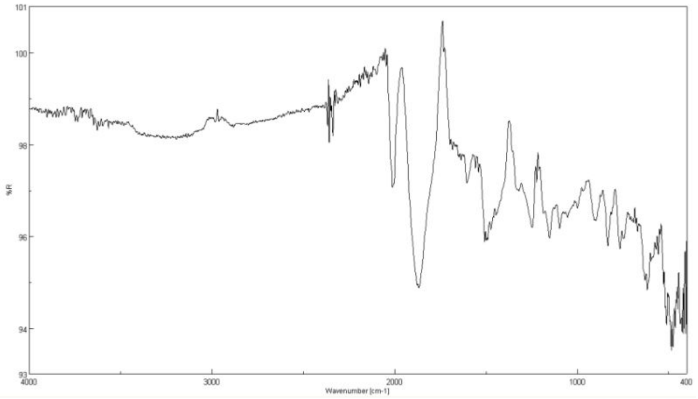

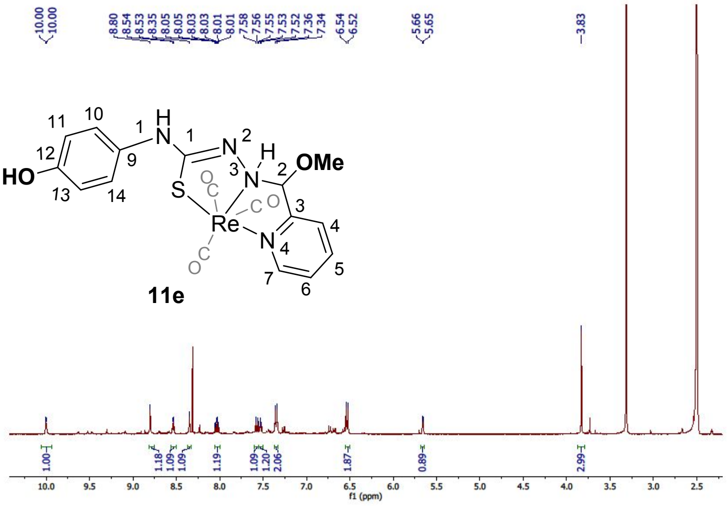

IR (top) and ^{1}H-NMR spectra in DMSO-d$_6$ (bottom).

Formation of the trinuclear complex $[Re_3Cl_2(L)(HL)(CO)_9]$

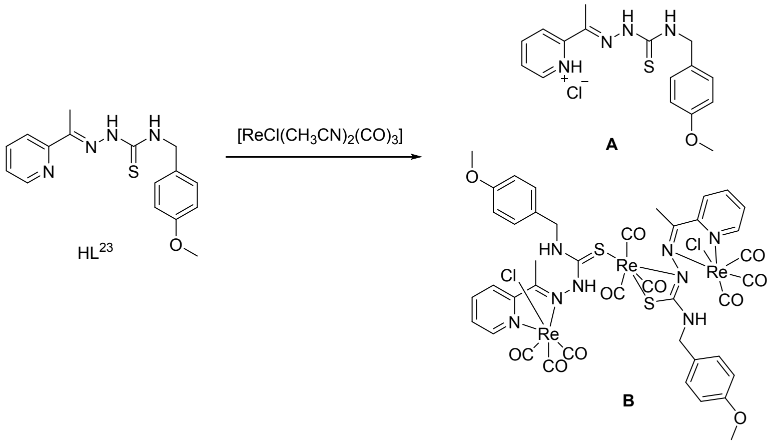

fac-$[ReCl(CH_3CN)_2(CO)_3]$ (47 mg, 0.12 mmol) and **HL[23]** (52 mg; 0.17 mmol) were dissolved on $CHCl_3$ (10 mL) and the solution refluxed for three hours. The resulting solution was concentrated to half its initial volume and stored at 4 °C. The first solid formed was the chlorohydrate salt of ligand (A). The second fraction crystallized (B) was filtered off and vacuum dried on $CaCl_2$/KOH.

$[Re_3Cl_2(L^{23})(HL)(CO)_9].3(CHCl_3)$

Yield: 35 mg (15 %). $C_{41}H_{35}Cl_2N_8O_{11}Re_3S_2 \cdot 2(C_{16}H_{18}N_4OS)$ (2138.2): calcd. C 41.0, H 3.4, N 10.5, S 6.0; found. C 41.0, H 3.5, N 10.2, S 5.8 %. MS-ESI [m/z (%)]: 315 (100) $|HL+H|^+$, 585 (90) $|Re(CO)_3(HL)|^+$, 899 (10) $|Re(CO)_3(HL)_2|^+$, 1205 (4) $|Re_2(CO)_6(HL)_2Cl|^+$, 1437 (3) $|M-Cl_2|^+$. IR data (ATR, ν/cm^{-1}): 3169b ν(NH, OH); 2011s, 1881vs ν($C=O_{fac}$); 1565m, 1509s, 1439m, ν(C=N); 1029s ν($O-CH_3$); 763s ν(C=S).

^1H NMR (400 MHz, DMSO-d_6, ppm):

HL: 10.63 (*s*, 1H, N2H), 9.50 (*s*, 1H, N1H), 8.69 (*d*, ^3J=5.2 Hz, 1H, C7H), 8.40 (*d*, ^3J=8.1 Hz, 1H, C4H), 8.10 (*m*, 1H, C5H), 7.61 (*m*, 1H, C6H), 7.32 (*d*, ^3J=8.7 Hz, 2H, C10H, C14H), 6.88 (*d*, ^3J=8.8 Hz, 2H, C11H, C13H), 4.81 (*d*, ^3J=6.2 Hz, 1H, C8H), 3.72 (*s*, 3H, C16H), 2.41 (*s*, 3H, C15H).

L: 11.59 (*s*, 1H, N1H), 9.03 (*d*, ^3J=5.4 Hz, 1H, C7H), 8.47 (*d*, ^3J=8.2 Hz, 1H, C4H), 8.35 (*m*, 1H, C5H), 7.86 (*m*, 1H, C6H), 7.26 (*d*, ^3J=8.4 Hz, 2H, C10H, C14H), 6.87 (*d*, ^3J=8.4 Hz, 2H, C11H, C13H), 4.81 (*d*, ^3J=6.2 Hz, 2H, C8H), 3.71 (*s*, 3H, C16H), 2.67/2.64 (*s*, 3H, C15H).

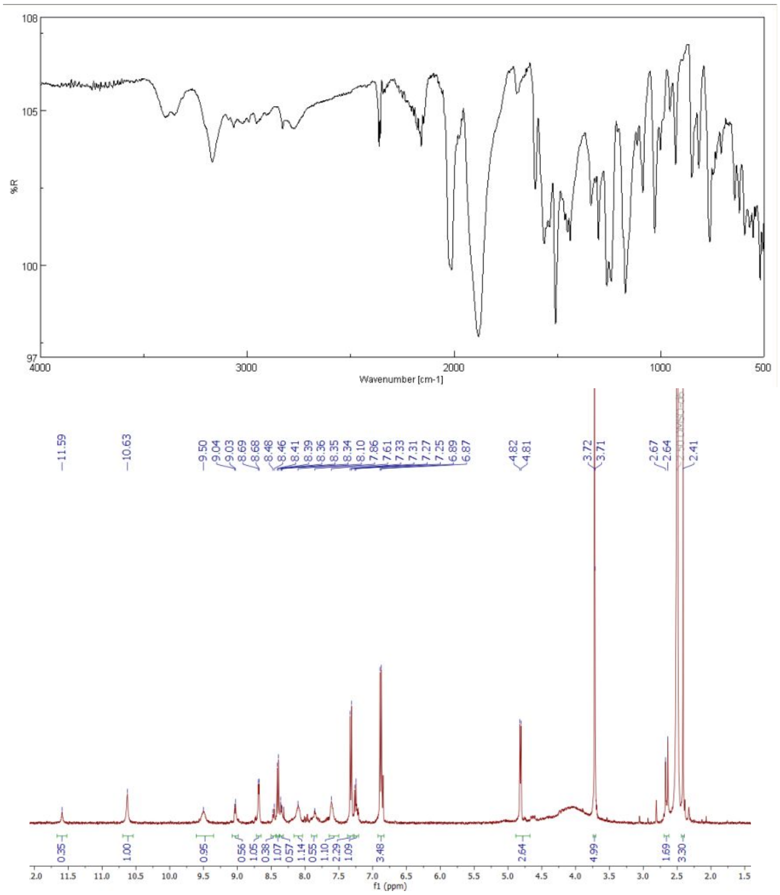

IR (top) and ^{1}H-NMR spectra in DMSO-d_6 (bottom).

Formation of the dinuclear complex [$Re_2(L^{13})_2(CO)_6$] (13f)

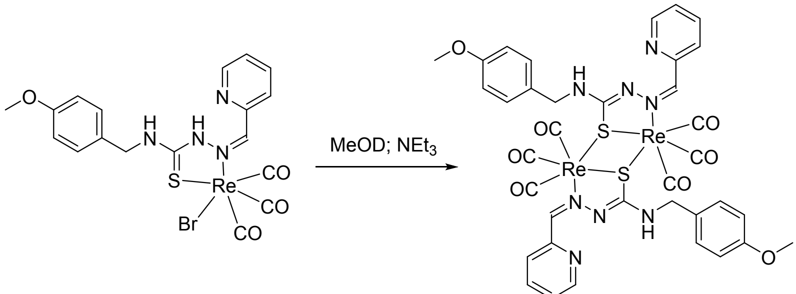

13b **13f**

In a NMR tube the **13b** complex was dissolved on MeOD, and NEt_3 was added (about 15 eq). The signals pattern of the product **13f** appeared while the 13b signals disappear of the spectrum. A small portion of crystals was isolated that allowed characterization by X-ray diffraction and infrared spectroscopy and confirmed the formation of the dimer. The ^{1}H-NMR shows two set of signals.

IR data (ATR, ν/cm^{-1}): 3308b ν(NH, OH); 2009s, 1925vs, 1892s ν(C=O$_{fac}$); 1532s, 1512vs ν(C=N); 1031s ν(O-CH$_3$); 769m ν(C=S).

^{1}H NMR (400 MHz, CD_3OD, ppm): *most abundant specie:* 8.77 (*d*, ^3J=4.5 Hz, 1H, C7H), 8.54 (*d*, ^3J=8.0 Hz, 1H, C4H), 8.40 (*s*, 1H, C2H), 7.55 (*td*, ^3J=7.9 Hz, ^4J=1.8 Hz, 1H, C5H), 7.43 (*dd*, ^3J=8.0 Hz, ^3J=4.5 Hz, 1H, C6H), 7.20 (*d*, ^3J=8.7 Hz, 2H, C10H, C14H), 6.84 (*d*, ^3J=8.7 Hz, 2H, C11H, C13H), 4.20 (*q*, ^2J=14.5 Hz, 2H, C8H), 3.75 (*s*, 3H, C16H). *Least abundant specie: b:* 8.61 (*d*, ^3J=4.9 Hz, 1H, C7H), 8.33 (*s*, 1H, C2H), 7.74 (*br*, 1H, C5H), 7.37 (*m*, 2H, C4H, C6H), 7.29 (*d*, ^3J=8.5 Hz, 2H, C10H, C14H), 6.89 (*d*, ^3J=8.6 Hz, 2H, C11H, C13H), 4.59 (*br*, 2H, C8H), 3.76 (*s*, 3H, C16H).

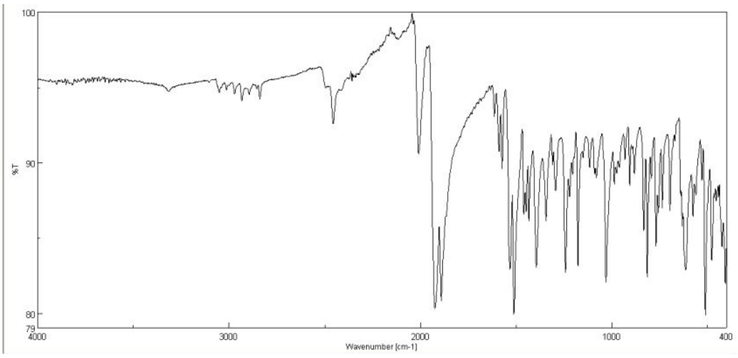

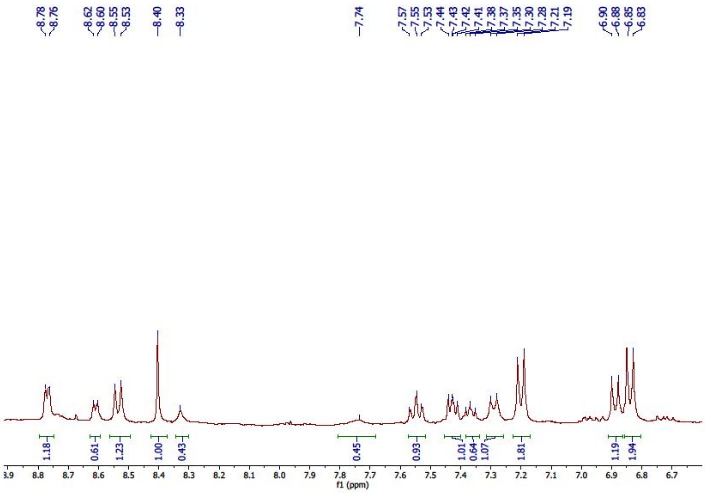

IR (top) and ^{1}H-NMR spectra in MeOH-d_4 (bottom).

References:

1 Nuñez-Montenegro, A.; Argibay-Otero, S.; Carballo, R.; Graña, A.; Vázquez-López, E.M. *Cryst. Growth & Des.* **2017**, *17*, 3338-3349. DOI: 10.1021/acs.cgd.7b00304

2 Pino-Cuevas, A.; Graña, A.; Abram, U.; Carballo, R.; Vázquez-López, E.M. *CrystEngComm* **2018**, *20*, 4781-92. DOI: 10.1039/C8CE00874D
